# Dimensionality reduction of quantitative EEG and clinical profiles uncover associations with monogenic neurodevelopmental phenotypes in SNAREopathies

**DOI:** 10.3389/fnins.2025.1725623

**Published:** 2026-01-27

**Authors:** Additya Sharma, Shilpa Anand, Cece C. Kooper, Michel J. A. M. van Putten, Arthur-Ervin Avramiea, Marina Diachenko, Arianne Bouman, Winde Mercken, Jennifer R. Ramautar, Huibert D. Mansvelder, Mathijs Verhage, Tjitske Kleefstra, Hilgo Bruining, Klaus Linkenkaer-Hansen

**Affiliations:** 1 Department of Integrative Neurophysiology, Centre for Neurogenomics and Cognitive Research (CNCR), Amsterdam Neuroscience, Vrije Universiteit Amsterdam, Amsterdam, Netherlands; 2 Department of Child and Adolescent Psychiatry and Psychosocial Care, Emma Children's Hospital, Amsterdam University Medical Centre (UMC), Amsterdam, Netherlands; 3 Emma Neuroscience Group, Department of Paediatrics, Emma Children's Hospital, Amsterdam Universtiet Medische Centrum (UMC), University of Amsterdam, Amsterdam, Netherlands; 4 Amsterdam Reproduction and Development Research Institute, Amsterdam, Netherlands; 5 Department of Clinical Neurophysiology, Medisch Spectrum Twente, Enschede, Netherlands; 6 Clinical Neurophysiology Group, University of Twente, Enschede, Netherlands; 7 Department of Human Genetics, Radboud University Medical Center, Nijmegen, Netherlands; 8 Department of Clinical Genetics, Erasmus Medische Centrum (MC), Rotterdam, Netherlands; 9 Emma Center for Personalized Medicine, Amsterdam University Medical Center, University of Amsterdam, Amsterdam, Netherlands; 10 N=You Neurodevelopmental Precision Center, Amsterdam Neuroscience, Amsterdam Reproduction and Development, Amsterdam UMC, Amsterdam, Netherlands; 11 Department of Human Genetics, Center for Neurogenomics and Cognitive Research, Amsterdam Neuroscience, Vrije Universiteit Medical Center, Amsterdam, Netherlands; 12 Center of Excellence for Neuropsychiatry, Vincent van Gogh Institute for Psychiatry, Venray, Netherlands

**Keywords:** dimensionality reduction, neurodevelopment, qualitative EEG, quantitative EEG, SNAREopathies

## Abstract

**Introduction:**

Monogenic neurodevelopmental disorders (mNDDs) such as SNAREopathies exhibit complex electrophysiological features and diversity among clinical symptoms, complicating the mapping of electro-clinical relationships, essential for improving diagnosis and treatment monitoring. Establishing robust normative electrophysiological feature distributions from typically developing populations enables precise, individualized quantification of patient-specific abnormalities. Here, we introduce a multivariate framework to reveal patient-specific electrophysiological phenotypes and clinical severity dimensions of direct relevance for individual prognosis and therapeutic tracking.

**Methods:**

We analyzed resting-state electroencephalography (EEG) data from15 SNAREopathy subjects (*STXBP1* and *SYT1*) and 96 age-matched healthy controls. EEG biomarkers, including absolute power, relative power, and long-range temporal correlations (LRTC), were estimated across frequency bands and functional networks. Normative baselines of EEG features were established using principal component analysis (PCA) on controls. We computed patient deviations from normative distributions using Mahalanobis distances. We summarized clinical severity by applying PCA to assessments of motor, manual, communication, adaptive functioning, and severity ranking of qualitative EEG.

**Results:**

The normative qEEG space identified diffuse, spectro-spatial patterns for absolute power, while relative power and LRTC displayed frequency-specific distributions. Clinical PCA identified a primary dimension of clinical impairment integrating deficits in mobility, hand function, communication, and adaptive behavior, whereas the secondary component captured the severity of qualitative EEG abnormalities. Patient deviations from normative absolute and relative power correlated with the primary, while LRTC deviations aligned with the secondary severity component.

**Discussion:**

Normative qEEG deviance metrics correspond to distinct clinical severity dimensions in SNAREopathies, making them promising for tracking disorder progression and therapeutic response.

## Introduction

Monogenic neurodevelopmental disorders arise from mutations in single genes and are characterized by a spectrum of neurological, psychiatric, and behavioral symptoms. Despite their clear genetic etiology, these conditions often exhibit significant clinical heterogeneity, complicating both diagnosis and treatment (Geertjens et al., 2022; Marotta et al., 2024; Summers et al., 2024). A thorough understanding of how these various features co-occur and their relationships to underlying neurophysiological processes is critical for enhancing prognostic accuracy, providing informed family counseling, and developing individualized treatment regimens. Quantitative electroencephalography (qEEG) serves as a vital tool in this research landscape, offering a means to assess the abnormal brain dynamics in relation to clinical symptomatology through the analysis of neuronal oscillations. However, the challenge posed by the small sample sizes inherent in studies of rare disorders, coupled with the diverse clinical presentations, limits the applicability of conventional analytical methods.

SNAREopathies exemplify the intricate relationship between qEEG patterns and clinical phenotypes. Caused by mutations in neuronal SNARE proteins and their regulators, these disorders disrupt neurotransmission, leading to a range of complex neurological symptoms (Verhage and Sørensen, 2020). Notably, two SNARE genes implicated are *STXBP1* or Syntaxin Binding Protein 1 and *SYT1* or Synaptotagmin 1 (Verhage et al., 2000; Baker et al., 2015, 2018; Kovačević et al., 2018; Chen et al., 2020; Melland et al., 2022; Riggs et al., 2022; Park et al., 2023). Clinically, individuals with pathogenic mutations in *STXBP1* and *SYT1* present with heterogeneous manifestations of epileptic encephalopathies, developmental delay and motor deficits (Deprez et al., 2010; Saitsu et al., 2010; Fitzgerald et al., 2015; Stamberger et al., 2016; Vasudevan and Suri, 2017; Baker et al., 2018; Kovačević et al., 2018; Melland et al., 2022; Riggs et al., 2022; Park et al., 2023; van Boven et al., 2024). Mechanistically, these mutations disrupt synaptic vesicle docking and neurotransmitter release, typically shifting excitation/inhibition balance and neural dynamics within cortical microcircuits. In *STXBP1*, experimental models have demonstrated that haploinsufficiency impairs GABAergic synaptic transmission, leading to circuit-level disinhibition (Kovačević et al., 2018; Chen et al., 2020). These molecular and cellular findings align with qEEG evidence showing altered delta, alpha, and beta-band oscillatory power as well as reduced long-range temporal correlations (LRTC), suggesting a shift toward inhibition-dominated dynamics in cortical networks (Houtman et al., 2021; Cossu et al., 2024). In *SYT1*, disruptions in calcium-dependent exocytosis imply likely downstream alterations in large-scale cortical dynamics, though qEEG characterization is lacking for *SYT1* cohorts (Park et al., 2023; van Boven et al., 2024). The findings suggest molecular SNARE deficits affect synaptic dysregulation, microcircuit imbalance and quantitative EEG aberrations that reflect macroscopic cortical network dysfunction. Qualitative EEG assessments, based on expert visual inspection of EEG, are routinely used in clinical neurology to identify epileptiform activity, slowing, and other diagnostic abnormalities (Niedermeyer et al., 1999). EEG abnormalities reported in patients with SNAREopathies include burst-suppression patterns and multifocal epileptiform discharges in *STXBP1*; and high-amplitude, rhythmic low-frequency patterns in *SYT1* (Deprez et al., 2010; Stamberger et al., 2016; Baker et al., 2018; Lanoue et al., 2019; Melland et al., 2022; Riggs et al., 2022).

Despite these clinical and mechanistic insights, critical gaps remain in our understanding of SNAREopathies. qEEG characterization in *SYT1* cohorts remains unexplored, precluding direct comparisons of electrophysiological signatures across SNARE gene mutations and limiting our ability to establish gene-specific neurophysiological mechanisms. Moreover, while qualitative EEG abnormalities and developmental clinical scales have been documented independently, integrated quantitative frameworks that systematically link qEEG metrics to multi-domain behavioral and developmental severity measures do not exist. An integrated severity metric is advantageous over domain-specific analyses because it addresses the profound clinical heterogeneity inherent to SNAREopathies. Relying on domain-specific analyses is challenging since a single-domain cannot capture the overall disease burden in a multi-system disorder, where caregiver priorities often reflect improvements across seizures, development, and daily functioning, and many standard clinical assessments suffer from floor effects when applied to individuals with severe-to-profound intellectual disabilities, a common feature in SNAREopathies. These tools are often not sensitive enough to detect clinically meaningful differences or therapeutic benefits in the most severely affected patients. Conventional group-level analytical approaches obscure individual variability and are often statistically underpowered in rare disease research due to inherently small and heterogeneous patient cohorts; normative modeling strategies that quantify patient-specific electrophysiological deviations from typical neurodevelopment have not yet been applied to monogenic neurodevelopmental disorders.

In the current study, we address these gaps through an integrated framework to characterize the electro-clinical signatures of SNAREopathies by linking high-density, resting-state qEEG from *STXBP1* and *SYT1* patients to a normative cohort of typically developing children. We employed a multivariate statistical approach using Mahalanobis distances to quantify the magnitude and pattern of each patient's deviation from typical neurodevelopmental trajectories (Gyebnár et al., 2019; Pressl et al., 2019). To capture clinical severity across multiple domains, we developed a novel integrated scheme that combines visually ranked qualitative EEG abnormalities with established behavioral and developmental clinical scales, thereby establishing multi-dimensional clinical severity axes that encompass neurophysiological, motor, and developmental aspects of the disorder. We further investigated how qEEG deviation scores relate to the multi-domain clinical severity measures, thereby establishing an integrated electro-clinical characterization applicable at the level of the individual subject (Lombroso, 1997; Palisano et al., 1997; Hidecker et al., 2011; Mehta et al., 2015; Eliasson et al., 2017; Fisher et al., 2017; Burger-Caplan et al., 2018; Kozhushko et al., 2018; Nicotera et al., 2019; Liang and Mody, 2022). By grounding patient assessment in objective electrophysiological biomarkers and individually referenced normative deviations, this framework enables more precise characterization of brain dysfunction while illuminating the neurophysiological mechanisms underlying clinical heterogeneity in SNAREopathies. Ultimately, the integration of quantitative EEG evidence with comprehensive clinical phenotyping provides a foundation for enhanced prognostic accuracy, informed genetic counseling for families, and the development of personalized, mechanism-informed therapeutic strategies.

## Methods

### Cohort demographics

**Figure 1:**
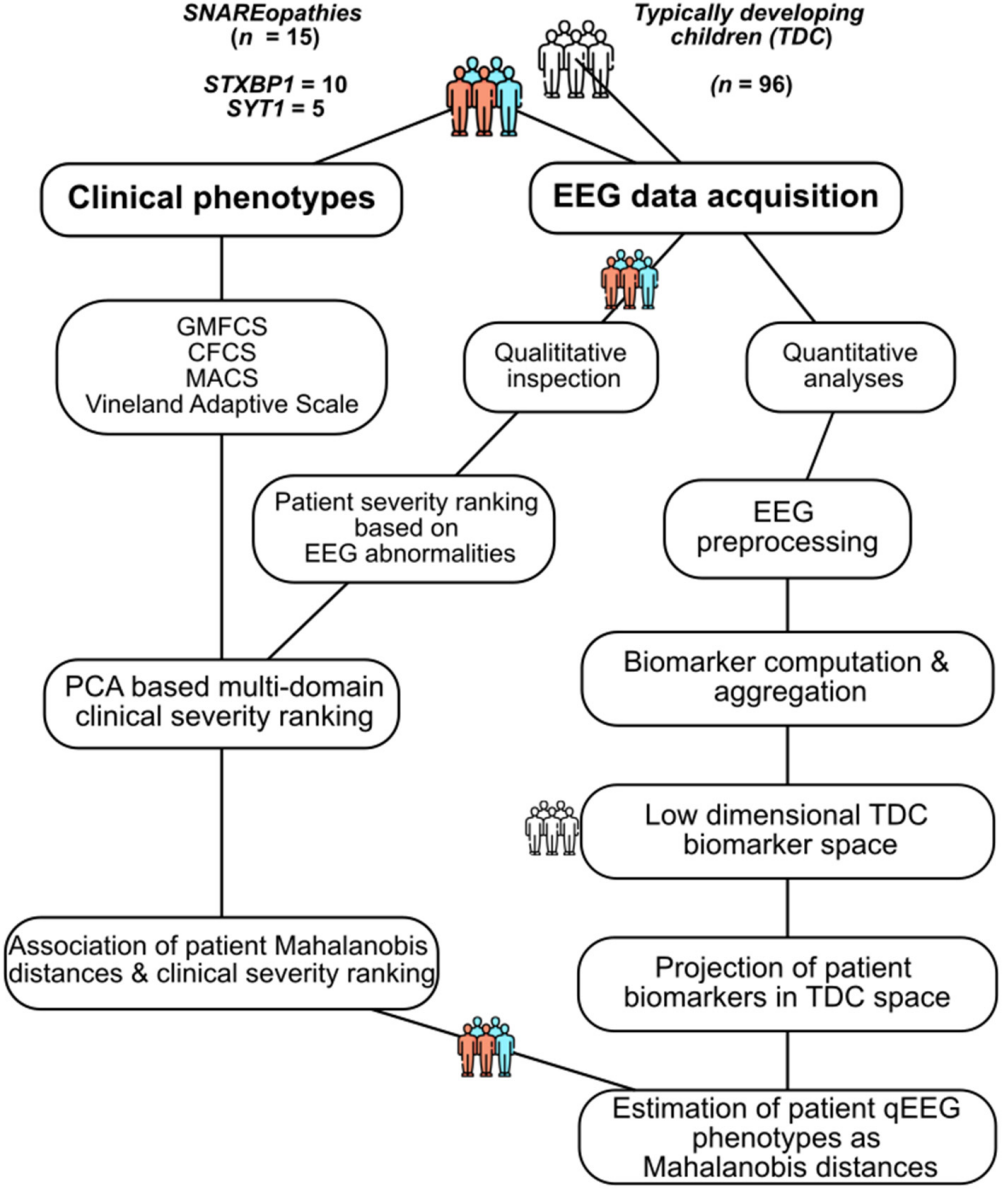
Methodological workflow. Overview of the methodological workflow of the study. Blue and orange colors represent the SNAREopathy patient cohort (*n* = 15). Clinical phenotyping and qualitative inspection of EEG abnormalities was performed only for the patient cohort. Low dimensional TDC biomarker space was based on the TDC cohort (*n* = 96) only. GMFCS, Gross Motor Functional Classification System; CFCS, Communication Function Classification System; MACS, Manual Ability Classification System.

This subsection describes the patient and typically developing children cohorts, including demographic characteristics and inclusion/exclusion criteria (Figure 1). Patients were recruited as part of a multicentre prospective observational study to develop personalized excitation-inhibition targeting treatments for genetic neurodevelopmental disorders (Geertjens et al., 2022). Data was collected from patients with *STXBP1* syndrome [*n* = 10 (8 males, 2 females); age range: 5–12.8 years; mean age (± S.D.) = 8 ± 2.2 years], and *SYT1* syndrome [*n* = 5 (4 males, 1 female); 5–18.5 years; 11 ± 4.6 years], and age- and birth sex-matched group of children with typical development [TDC; *n* = 96; age range: 4.3–18.2 years; mean age (± S.D.) = 9.6 ± 3.6 years; 59 males, 37 females]. Age is reported at the time of EEG recording.

Patient exclusion criteria included additional causative genes involved, larger genetic defects that influence the gene-of-interest related phenotype, extremely or very preterm birth (< 32 weeks of pregnancy), presence of severe psychiatric disease and/or serious, unstable illnesses. TDC were recruited in a dedicated study for controls in neuroscientific studies (Kooper et al., 2022, 2024; Ramautar et al., 2025). The exclusion criteria included a history of behavioral or learning problems, a diagnosis of any neurodevelopmental condition, or any other health issue. We did not consider the handedness of all subjects and the menstrual cycle of individual female subjects (patients and TDC) in the study (for justification, see Supplementary Methods S1, S5). Written informed consent was provided by all patients aged > 11 years and legal guardians of children aged < 16 years. The study was approved by the Ethics Review Committee and institutional review boards at the Amsterdam University Medical Centre and Radboud University Medical Center, conducted in accordance with the provisions of the declaration of Helsinki and good clinical practice. The TDC study obtained approval from the Medical Ethical Committee Amsterdam University Medical Center (location AMC, reference number NL76915.018.21).

### Clinical profiles

This subsection summarizes the clinical information collected to characterize neurodevelopmental severity across the patient cohort. Clinical severity was characterized using four standardized and validated measures. Adaptive functioning was quantified using the Vineland Adaptive Behavior Composite (ABC) score (range 22–101), with lower scores indicating greater developmental delay and scores near 100 reflecting age-appropriate abilities (Burger-Caplan et al., 2018). Gross motor function, manual dexterity, and communication ability were classified using the five-level Gross Motor Function Classification System (GMFCS), Manual Ability Classification System (MACS), and Communication Function Classification System (CFCS), respectively (Palisano et al., 1997; Hidecker et al., 2011; Eliasson et al., 2017). MACS measures hand function and object manipulation in daily activities; CFCS quantifies everyday communication effectiveness. On all three functional scales, Level I indicates minimal impairment and Level V indicates severe impairment requiring assistance. Additionally, we recorded age, sex, mutation type, inheritance pattern (de novo vs. inherited), seizure history (age of onset, current seizure status, antiepileptic drug response), current medications at time of EEG recording, and reported comorbidities.

### EEG data acquisition

This subsection describes the EEG recording system, acquisition parameters, and recording conditions. All EEG recordings used the same high-density 129-channel system and acquisition parameters. EEG recordings were acquired during awake, eyes-open and rest (EOR) with a duration between 230 and 600 s for patients. The EOR condition was selected for patients because children with SNAREopathies frequently have difficulty maintaining their eyes closed for the 5–10 min that a resting-state recording takes. The EOR condition therefore maximizes usable recording time and data reliability in this population. Patient recordings were subsequently assessed for quantitative and qualitative EEG features. Qualitative assessment was performed to capture clinically interpretable EEG abnormalities and summarize their overall burden into an ordinal severity score, complementary to (but distinct from) quantitative biomarker analyses (see next section). For TDC, both resting EOR and eyes-closed (ECR) were acquired with a duration of 300s. TDC eyes-closed resting (ECR) condition was acquired enabling validation of the normative space against known physiological state differences. TDC recordings were subsequently used for assessing quantitative EEG. For TDC, after verbal consent, either a visit to the Emma Children's Hospital, or a visit to our mobile laboratory (“Emma Brain Bus”) was planned. High-density 129 channel HydroCel Geodesic with the NetAmps400 amplifier (Magstim-Electrical Geodesics Inc.) were used. The acquisition sampling rate was 1,000 Hz with reference electrode Cz and a ground electrode “COM” between CPz and Pz. Electrode impedances were kept below 100 kΩ.

### Visual inspection and severity ranking of patients based on qualitative EEG abnormalities

This subsection details how qualitative EEG abnormalities were visually assessed and summarized into an ordinal severity ranking. Qualitative EEG abnormalities are commonly reported in SNAREopathies and are routinely used in clinical practice to support assessments of disorder severity (Deprez et al., 2010; Baker et al., 2018; Verhage and Sørensen, 2020; Riggs et al., 2022). Hence, resting-state EEG recordings of the SNAREopathy cohort were reviewed by a board-certified clinical neurophysiologist (M.v.P) using NeuroCenter^®^ EEG (Clinical Science Systems), using a subset of electrodes corresponding to the international 10–20 system. Inspection was performed using Laplacian, bipolar and common average montages, with (out) bandpass filtering between 0.5 and 35 Hz. The frontal, central, parietal, and temporal electrodes were classified as the anterior, and the occipito-parietal electrodes as the posterior regions. EEG abnormalities were identified based on the posterior dominant rhythm, anterior-posterior rhythm gradient and the spectral characteristics at each EEG electrode. Abnormalities were classified as “focal”, “global”, or “focal-global” (Lodder and van Putten, 2011, 2013).

To quantify the overall burden of neurophysiological dysfunction, we constructed a cumulative ordinal severity scale, adapted from the qualitative EEG scoring approach of Waal et al. (2011). Abnormalities were categorized and weighted based on their clinical significance. Focal abnormalities, defined as transient spectral alterations observed at one or more electrodes, graded as low severity (severity score of two). Global abnormalities, characterized by a dominant background rhythmic frequency below 8 Hz (slow posterior dominant rhythm), disrupted anterior-posterior rhythm gradients and burst suppression patterns, were graded as most severe (severity score of five). Focal-global abnormalities were defined as patterns exhibiting both localized transient spectral abnormalities and global dysregulation of background activity. These were graded as moderate severity and further differentiated into epileptiform discharges (severity scores of three) and diffuse beta activity severity scores of four; (Supplementary Methods S2, S3). We assigned ordinal severity rank to capture the co-occurrence and complexity of abnormalities within a single metric. Patients with no abnormalities were assigned a rank of 1. For patients with abnormalities, the rank was calculated as the sum of the weights of all identified distinct abnormalities. For example, a patient with both epileptiform discharges (Weight 3) and a slow PDR (Weight 5) would receive a severity rank of 8. This additive scoring rubric captures the cumulative burden of multiple neurophysiological disruptions. Scoring was performed by a single board-certified clinical neurophysiologist (M.v.P.); hence, inter-rater reliability was not calculated.

### Principal component analysis of clinical scales

This subsection describes how clinical severity was reduced to low-dimensional axes using principal component analysis applied to multi-domain clinical assessments. Principal Component Analysis (PCA) was applied to characterize the clinical severity of the patient cohort. To assess the suitability of PCA across clinical scales in a small sample sized patient cohort, we performed the Kaiser-Meyer-Olkin test (KMO score) for sampling adequacy and Bartlett's test of sphericity (χ^2^, p_Bartlett_)_._ The PCA analysis included the scores on Vineland ABC, GMFCS, MACS, CFCS, and the clinician-assessed severity ranking of qualitative EEG abnormalities. To account for the ordinal nature of the clinical scales, each scale was rank transformed across patients. Then, a Spearman correlation matrix was computed from the ranked data. An eigen decomposition of the correlation matrix was performed to identify principal components explaining the greatest variance. The first two principal components were retained (PC1_clin_, PC2_clin_). Finally, patient scores were calculated by projecting the z-scored rank data onto the corresponding eigenvectors. In addition to PC-specific scores, we summarized overall displacement of each patient in the two-dimensional clinical space using variance-standardized Mahalanobis distance. This metric quantifies overall displacement from the cohort center, while scaling deviations along each principal component axis (PC1_clin_, PC2_clin_) by the inverse of the eigenvalue. This approach prevents the systematic under-weighting of deviations along lower-variance components.

### Resting-state EEG preprocessing

Preprocessing followed an established pipeline optimized for pediatric high-density EEG and designed to preserve oscillatory dynamics relevant to qEEG biomarkers. Raw continuous EEG recordings were pre-processed using EEGLAB (v2022.0v, MATLAB R2021b). EEG signals were downsampled to 250 Hz. A 50 Hz notch filter and a 1–45 Hz band-pass filter were applied (Delorme and Makeig, 2004; Ramautar et al., 2025). Electrodes with poor signal-to-noise ratio and flat signals were automatically detected using the Random Sample Consensus algorithm (Fischler and Bolles, 1981), and transient and large-amplitude artifacts were simultaneously flagged using the Artifact Subspace Reconstruction algorithm (Kothe and Makeig, 2013). All recordings were manually inspected to decide which flagged channels and artifact-contaminated segments to reject or retain based on visual assessment. Signals from rejected noisy electrodes were then interpolated using the spherical spline method. Signals were re-referenced to the average of all electrodes using full-rank average referencing to preserve data rank and accommodate the interpolated channels. Using Independent Component Analysis (Infomax) algorithm and ICLabel, all recordings were screened for eye movements/blinks, heartbeat and muscle-related artifacts and projected out from the signals (Bell and Sejnowski, 1995; Pion-Tonachini et al., 2019). A final manual inspection was performed to identify and reject any residual artifacts not captured by automated component classification (see Supplementary Methods S4 for preprocessing pipeline parameters and artifact details).

### EEG source reconstruction

Scalp-level EEG data were projected into cortical source space to improve spatial specificity and reduce volume conduction effects. L2 minimum norm estimation (MNE-Python) was used to obtain cortical surface current estimates from the electrode level data (Ramautar et al., 2025; Li et al., 2022; Gramfort et al., 2014). The FreeSurfer average brain template from FreeSurfer 6 was used to construct the boundary element head model and forward operator for the source modeling (Fischl, 2012). The regularization parameter was set to λ^2^ = 1/9. A diagonal matrix with 0.2 variance level was used for the covariance matrix. Unconstrained dipole orientations were allowed. PCA was then applied to reduce the three-dimensional signals of source time series at each vertex to a one-dimensional time series of the dominant principal component. The time series of 20,484 source vertices were further collapsed into 100 cortical patches derived from the Schaefer atlas (Schaefer et al., 2018). Reducing the 20,484 source vertices to 100 parcels substantially decreases computational burden and feature dimensionality while preserving network-level organization relevant to qEEG biomarker characterization. All qEEG biomarkers were estimated for individual Schaefer patch waveforms obtained after source reconstruction. The individual Schaefer patches were mapped to the Yeo atlas based large-scale functional brain networks in the left and right hemispheres, resulting in 7 networks: Default (DEF), Somato-motor (SOM), Salience Ventral Attention (SVA), Dorsal Attention (DA), Visual (VIS), Limbic (LIM), and Control (CON) networks (see Supplementary Methods S6 for Schaefer-to-Yeo Network mapping) (Schaefer et al., 2018). The Schaefer 100-parcel atlas was chosen as a well-validated, publicly available parcellation that balances spatial resolution and aligns with the Yeo functional network definitions used in subsequent analyses. Organizing biomarkers by functional networks rather than arbitrary anatomical regions allows characterization of qEEG signatures within neurobiologically coherent systems implicated in motor control, attention, emotion regulation, and cognitive processing. This network-level perspective is particularly valuable for rare neurodevelopmental disorders where dysfunction likely reflects distributed circuit abnormalities rather than focal lesions.

### EEG biomarker estimation and aggregation

This subsection describes how EEG biomarkers of spectral power and long-range temporal correlations were defined, estimated, and aggregated across frequency bands and cortical networks.

#### Frequency bands

Eleven frequency bands were defined within 1–45 Hz, one band from 1 to 4 Hz and ten logarithmically spaced bands between 4 and 45 Hz, following the procedure described in Diachenko et al. (2024). The resulting frequency bands were: 1–4 Hz, 4–5.1 Hz, 5.1–6.5 Hz, 6.5–8.3 Hz, 8.3–10.5 Hz, 10.5–13.4 Hz, 13.4–17 Hz, 17–21.7 Hz, 21.7–27.6 Hz, 27.6–35.2 Hz, and 35.2–44.8 Hz.

#### Absolute and relative spectral power

The power spectral density (PSD) was computed by filtering the individual Schaefer patch time-series in the eleven log-spaced frequency bands from 1 to 45 Hz (Diachenko et al., 2024). For each frequency band, PSD was estimated using Welch's method with a Hamming window, implemented in the MNE software (Gramfort et al., 2014). The length of the Fast Fourier Transform (FFT) was set to ten times the sampling frequency, resulting in a frequency resolution of 0.1 Hz. Each segment used a Hamming window equal in length to the FFT length, and window overlap was set to 50% of the FFT length, rounded down to the nearest integer. Absolute power for each of the eleven frequency bands was calculated by integrating the PSD across the respective frequency band and then converting the result to a decibel (dB) scale. Relative power was computed as the ratio of each band's integrated PSD to the total integrated PSD across the full 1–45 Hz frequency range and expressed in percent (%).

#### Detrended fluctuation analysis

DFA quantifies long-range temporal dependencies in neural oscillations, which reflect excitation–inhibition dynamics at the network level (Linkenkaer-Hansen et al., 2005; Hardstone et al., 2012; Houtman et al., 2021; Diachenko et al., 2024). Autocorrelation in the amplitude envelope of each frequency band was quantified by the DFA exponent, which relates the mean fluctuation in the amplitude to increasing time-window sizes within a chosen time-scale range (Linkenkaer-Hansen et al., 2001; Hardstone et al., 2012). A DFA exponent of 0.5 indicates an uncorrelated random signal, whereas values >0.5 denote positive autocorrelation whose strength grows with the exponent. Before computing DFA, each signal was bandpass filtered into 11 frequency bands, and the amplitude envelope for each band was extracted using a Hilbert transform. Bandpass filtering and Hilbert transformation were implemented in the MNE software (Gramfort et al., 2014). For each frequency band, the DFA exponent was obtained as the slope of the line relating the log-fluctuation to the log-window size, fitted using the time-scale ranges (Diachenko et al., 2024).

#### EEG biomarker aggregation

Absolute power, relative power, and DFA exponents were computed across 11 logarithmically spaced frequency bins and grouped into five canonical bands: delta (δ: 1–4 Hz), theta (θ: 4–8.2 Hz), alpha (α: 8.2–13.3 Hz), beta (β: 13.3–21.7 Hz), and high beta/gamma (β-γ: 21.7–44.8 Hz). The canonical bands are neurophysiologically meaningful and reduce the dimensionality to deal with multiple comparison burden. For each of the 100 Schaefer cortical parcels, power biomarkers were sum-aggregated and DFA exponents were median-aggregated within each canonical frequency band. Median aggregation was preferred due to small number of samples and heterogeneity within the patient cohort. Leveraging the established mapping of Schaefer parcels to Yeo functional networks, the network-level estimates for each EEG biomarker were computed as the median across parcels associated with each Yeo network, separately for the left and right hemispheres (Schaefer et al., 2018). Global network-level estimates were derived by computing an average across hemispheres (see Supplementary Methods S7). This resulted in a final feature matrix of 35 EEG features per biomarker per subject, across five canonical frequency bands and seven Yeo networks. The 35 features (5 bands × 7 networks) provided a compact yet comprehensive representation of spectro-spatial qEEG organization while ensuring the feature count remained below the TDC sample size (*n* = 96), a prerequisite for stable covariance estimation and PCA.

### Low-dimensional TDC biomarker space

We constructed a low-dimensional normative EEG feature space from TDC cohort using sparse principal component analysis. Characterizing typical patterns of qEEG organization during healthy development is essential for identifying meaningful deviations in patient populations. The reference space captured the spectral and spatial distribution of absolute power, relative power, and LRTC across canonical cortical networks and frequency bands. The original feature space consisted of 100 cortical parcellations across 11 frequency bins, yielding 1,100 features for each of the three biomarkers. To reduce dimensionality and enhance interpretability, we aggregated biomarkers into 35 features per TDC, across five frequency bands and seven Yeo functional networks. Next, we constructed a normative spatio-spectral reference space using eyes-open resting-state (EOR) qEEG from TDC. We applied principal component analysis (PCA) with a sparsity constraint on the loading vectors. This transformed the 35-feature matrix into a set of orthogonal low-dimensional representations of the salient spectro-spatial features in TDC. Sparse PCA was chosen over standard PCA because the sparsity constraint forces many feature loadings to zero, yielding components dominated by a subset of features rather than dense linear combinations of all 35 features. This enhances neurophysiological interpretability and facilitates identification of which frequency bands and networks drive each component (Zou et al., 2006; Jolliffe and Cadima, 2016). Median and interquartile range scaling parameters were estimated on the TDC and applied to both TDC and patient datasets. Sparse PCA was subsequently applied separately for each TDC biomarker feature matrix. This approach ensured that the total number of features per model (*n* = 35) remained below the TDC sample size (*n* = 96), which is necessary for stable covariance estimation in subsequent multivariate Mahalanobis distance estimations. The normative subspace was derived without patient data, mitigating potential bias arising from the typically heterogeneous patient cohort. For each biomarker, we set the sparsity parameter to 1.5 and found that the top 5 principal components captured more than 70% of the variance, establishing the reference structure used for patient-specific comparisons (see Supplementary Methods S9 and Supplementary Figure S1).

### Quantification of TDC and patient Mahalanobis distances

This subsection explains how Mahalanobis distances (MD) were computed to quantify individual deviations from the normative EEG feature space. For each biomarker of the TDC EOR, we examined pairs of principal components, to represent qEEG features in two-dimensional spaces reflecting healthy brain activity patterns. The Minimum Covariance Determinant estimator was applied to compute robust center points and covariance matrices that remain unaffected by potential outliers (Rousseeuw and Driessen, 1999). Two types of normative centers were defined: a global center based on the entire TDC EOR cohort (*n* = 96), and age-matched centers based on TDC EOR within ±12 months of each patient's age. The ±12-month window was selected to capture sufficient TDC for covariance estimation (minimum *n* ~ 10–15 per patient) while restricting the comparison to a developmentally similar reference group, given the rapid EEG maturation occurring during childhood and adolescence (Clarke et al., 2001; Whitford et al., 2007). For each TDC EOR, a leave-one-out jackknife approach was used to estimate their distance from the global normative structure (Efron and Stein, 1981). For each TDC EOR, we temporarily removed the subject from the reference set, fitted the robust covariance estimator to the remaining points, and calculated the Mahalanobis distance between the removed point and the center of the remaining points (Mahalanobis, 2018; Gyebnár et al., 2019; Pressl et al., 2019). MD measures how far a subject's multivariate qEEG point lies from the normative center after taking the normative covariance structure into account: deviations along directions that are naturally variable in the normative cohort contribute less to the distance, whereas deviations along directions that are typically stable contribute more. In addition, when two (or more) qEEG features tend to move together in the normative data, MD treats a joint shift in those correlated features as largely one underlying deviation (rather than multiple independent deviations), because the covariance encodes the redundancy. A higher MD indicates a profile that is statistically more distant from the normative center, reflecting greater overall neurophysiological abnormality.

To test sensitivity to known neurophysiological variation, we projected TDC eyes-closed resting (ECR) data onto the same TDC EOR reference space. We then computed their MD to the global TDC EOR centre (MD_global_) (Barry et al., 2007; Wei et al., 2018; Wang et al., 2022). SNAREopathy patient data were similarly projected into the normative space. MD was calculated relative to both the global TDC EOR centre (MD_global_) and the age-matched center (MD_age_) yielding two complementary metrics of patient-specific deviation from typical EEG organization. In all cases, MD was computed using:


$$\begin{array}{l} MD=\sqrt{{\left(x-u\right)}^{T}\Sigma^{-1}\left(x-\mu \right)} \end{array}$$

where *x* is the PCA score vector of the subject, *u* is the center of the normative EOR space (global or age-matched), and Σ is the robust covariance matrix estimated from the normative EOR data. By capturing the multivariate distance between an individual (TDC ECR/patient cohort) qEEG biomarker profile and the normative distribution, MD serves as a quantitative measure of deviation or atypicality. These distances were used both to validate the normative model across physiological states and to evaluate individual patient deviations in subsequent analyses. MD values were utilized in the raw form for statistical analysis.

### Statistical analysis

Statistical analysis was performed using custom scripts in Python (version 3.11.11).

#### Comparison of TDC distances between eyes closed and eyes open rest condition

We validated the EOR-derived normative space against the physiological contrast of ECR. ECR is a physiological manipulation known to enhance alpha power and modulate low-frequency activity in typically developing children and adults (Barry et al., 2007; Wang et al., 2022; Krukow et al., 2024; Clarke et al., 2001; Barry and De Blasio, 2017). We first tested whether the TDC EOR-based normative space was sensitive to physiological state differences by comparing Mahalanobis distances of the same TDC across EOR and ECR conditions (*n* = 96, repeated measurements available for all subjects). For each biomarker and PC pair (3 biomarkers x 10 PC pairs, e.g., for absolute power, combination of PC1–PC2, PC1–PC3…PC4–PC5), we compared EOR and ECR MD using paired statistical tests (number of paired tests = 30). Normality was assessed using the Shapiro-Wilk test. Wilcoxon signed-rank tests were used to compare the distances between the paired conditions. Multiple comparisons were corrected using Benjamini-Hochberg False Discovery Rate (FDR) within each biomarker block to preserve interpretability of biomarker clusters and avoid overconservative corrections. To assess the directionality and magnitude of change, we also tested whether the distribution of distance differences (ECR – EOR) differed significantly from zero.

#### Correlation between patient EEG deviations and clinical severity axes

This subsection details the statistical approach used to test associations between patient-specific EEG Mahalanobis distances and multidimensional clinical severity axes. The association was analyzed using non-parametric Spearman rank correlations (asymptotic Spearman's ρ). Within each biomarker, distance type, and clinical severity axis, we tested 10 correlations (one per PC pair), yielding in total 120 correlations (3 biomarkers × 10 PC pairs × 2 distance types × 2 clinical axes), with Benjamini-Hochberg FDR applied separately to each set of 10 tests at α = 0.05. Spearman rank correlation was employed to account for the ordinal nature of clinical scales. Further, MD are bounded below by zero and characteristically right skewed, violating normality assumptions that are difficult to establish reliably with the present sample size (*n* = 15). Spearman correlation captures monotonic relationships without imposing linearity assumptions, which is advantageous when investigating associations involving bounded ordinal scales that may exhibit ceiling or floor effects. Finally, the rank-based approach provides robustness against outlying observations that could disproportionately influence parametric estimates in small samples. To quantify the stability of tail estimates, observed correlations and minimize Monte Carlo error, we applied unstratified bootstrap resampling (15000 bootstraps) to generate 95% percentile confidence intervals for each ρ estimate. Statistical significance of observed ρ was tested using between-sample permutation tests (15,000 permutations), generating empirical null distributions. Two-tailed *p*-values were derived based on the proportion of permutations where the absolute correlation exceeded the observed ρ.

## Results

### SNAREopathies exhibit moderate to severe developmental delay and domain-specific functional impairments

We characterized four clinical phenotypes across the SNAREopathy cohort. Vineland ABC scores ranged from 22 to 101 across the cohort, with 13 out of 15 patients (10 *STXBP1*, 3 *SYT1*) scoring below 80, indicative of moderate to severe neurodevelopmental delay (Tables 1, 2). Seven patients (5 *STXBP1*, 2 *SYT1*) scored greater than level III on GMFCS, indicating marked limitations in mobility). The threshold distinguishes children who can walk with assistive devices (Level III) from those with severe mobility limitations requiring wheeled mobility (Levels IV-V) (Palisano et al., 1997). MACS scores exceeded level III in nine patients (7 *STXBP1*, 2 *SYT1*); scores in this range (>III) indicate a need for continuous assistance or adapted situations to handle objects, as opposed to the independent albeit difficult handling seen at Level III (Eliasson et al., 2017). Finally, nine patients (7 *STXBP1*, 2 *SYT1*) scored above level III on the CFCS, denoting inconsistent sender/receiver roles even with familiar partners, a marked reduction in functional communication compared to Level III (Hidecker et al., 2011). No significant differences between *STXBP1* and *SYT1* sub-groups across the clinical scales were observed (see Supplementary Methods S8, Supplementary Figure S2). In summary, functional classification systems revealed widespread impairments across motor and communication domains in the SNAREopathy cohort.

**Table 1:** *STXBP1* and *SYT1* cohort demographics and clinical features.

**#**	**Sex**	**Mutation site**	**Gene**	**Age (years)**	**Epilepsy age and history**	**Medications**	**Neurological features**	**Neuropsychiatric features**	**EEG abnormalities**
1	Male	C.227T>C	*STXBP1*	7.3	Focal onset, impaired awareness, motor—tonic, generalized onset non-motor (absence)—typical	Topiramate, Levetiracetam, Clobazam	Abnormality of extra-pyramidal motor function	Anxiety	Right fronto-temporal beta activity (80–90 uv)
2	Male	C.271delt	*STXBP1*	8.3	Focal onset, impaired awareness, motor automatisms, tonic, generalized onset non-motor (absence)—typical	Levetiracetam	Babinski sign, hypotonia, ataxia	Autism spectrum disorder, sensory sensitivity	Slow PDR (6–7 Hz), abnormal AP gradient
3	Male	C.721T>C	*STXBP1*	12.8	Generalized onset motor—tonic clonic	None	Generalized hypotonia, joint laxity	Not known	Global slowing delta (4 Hz) rhythm, abnormal AP gradient
4	Male	C.1111-1G>A	*STXBP1*	7.2	Generalized onset non-motor (absence)—typical, focal onset, aware, non-motor autonomic	Lamotrigine	Hypotonia	Not examined	Left fronto-temporal epileptiform discharges, spike-wave complex (3–4 Hz)
5	Male	C.1216C>T	*STXBP1*	7.6	No history of seizures	Gastro-intestinal, asthma	No	Autism spectrum disorder	Right hemispheric epileptiform discharges, frontal slow-wave activity, abnormal AP gradient
6	Female	C.578+1G>A	*STXBP1*	8.7	Focal onset, aware, motor automatisms, focal onset, aware, non-motor autonomic	Lamotrigine	No	Hyperactive/attention problems	Normal
7	Male	C.620A>G	*STXBP1*	10.8	No history	Methylphenidate (not taken on the day of EEG)	Intention tremor	Autism spectrum disorder	Diffuse high amplitude beta activity
8	Male	C.1500_1513del	*STXBP1*	5.0	No history	None	Hypotonia	Not present	Normal
9	Male	c.[1030-2A>G]	*STXBP1*	5.4	No history of seizures	Vitamin D	No	Sensory sensitivity	Slow PDR (5 Hz), abnormal AP gradient
10	Female	C.734A>G	*STXBP1*	7.0	Generalized onset, motor—tonic,	Levetiracetam	Generalized joint hypermobility, hypotonia, Babinski sign; broad-based gait	Examined, not present	Slow PDR (5 Hz) with intermittent abnormal high frequency oscillations
11	Female	C.647C>G	*SYT1*	18.5	No history	None	No	Not known	Normal
12	Female	C.587C>A	*SYT1*	7.4	No history	None	No	Not known	Diffuse frontal slow-wave theta (6–7Hz), delta activity (3 Hz)
13	Male	C.1202C>T	*SYT1*	11.6	No history	Bumetanide	No	Not known	Normal
14	Female	C.[925T>C]-[=]	*SYT1*	5.0	Focal onset, aware, motor—myoclonic	Levetiracetam	No	No verbal communication	Paroxysmal suppression of delta activity, epileptiform and slow-wave discharges
15	Female	C.1113C>G	*SYT1*	12.5	Generalized onset non-motor (absence)—typical, focal onset, aware, non-motor emotional, generalized onset motor—tonic clonic	Levetiracetam	No	No verbal communication, not examined	Left fronto-temporal slow-wave activity, fronto temporal epileptiform discharges, frontal slow-wave (2–3 Hz) activity

PDR, Posterior Dominant Rhythm; AP, Anterior-Posterior.

**Table 2:** *STXBP1, SYT1* clinical severity scales.

**#**	**Sex**	**Vineland AGI**	**GMFCS**	**MACS**	**CFCS**	**EEG abnormality rank**
1	Male	46	5	4	5	2
2	Male	44	5	5	5	7
3	Male	27	5	5	5	8
4	Male	35	5	5	5	3
5	Male	38	5	4	5	6
6	Female	58	2	3	4	1
7	Male	60	1	2	1	4
8	Male	56	2	4	3	1
9	Male	53	2	4	4	7
10	Female	77	1	1	1	5
11	Female	96	1	1	2	1
12	Female	60	1	1	2	5
13	Male	101	1	1	2	1
14	Female	46	5	5	4	8
15	Female	22	5	5	5	6

### High prevalence and heterogeneity of qualitative EEG abnormalities in SNAREopathies

**Figure 2:**
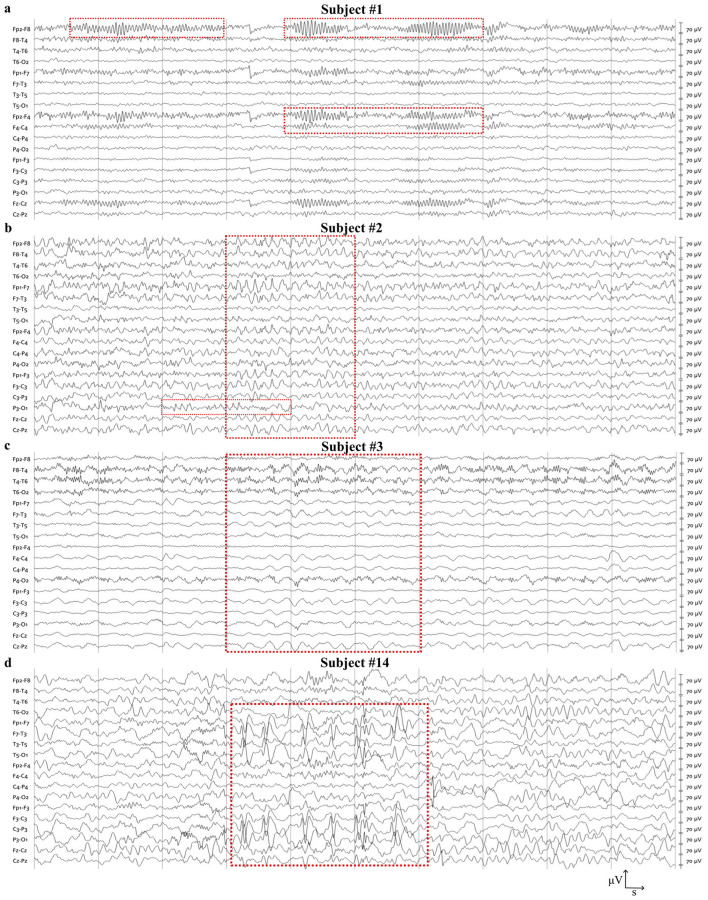
*STXBP1* and *SYT1* patients exhibit diverse types of qualitative EEG abnormalities. **(a)** Subject #1: age 7.3 years, condition *STXBP1*, mutation site c.227T>C with abnormal fronto-temporal beta activity in the right hemisphere. **(b)** Subject #2: age 5.4 years, condition *STXBP1*, mutation site c.[1030-2A>G] with slow Posterior Dominant Rhythm and abnormal Anterior-Posterior gradient. **(c)** Subject #3, age 7.2 years, condition, *STXBP1*, mutation site c.1111-1G>A with abnormal Anterior-Posterior gradient and global delta activity. **(d)** Subject #14, age 5 years mutation *SYT1*, mutation site c.[925T>C] with fronto-temporal epileptiform discharges and spike-wave activity in the left hemisphere. Red boxes highlight the location and extent of abnormalities. Gray lines indicate 1s resolution, Scale: x = 10 s, y = 70 μV.

SNAREopathies are known to exhibit different forms of qualitative EEG abnormalities. We characterized the presence and nature of abnormalities through visual inspection of EEG recordings. Our findings revealed that four of the 15 patients (27%; 2/10 STXBP1, 2/5 SYT1) displayed normal EEGs (Rank 1). The remaining 11 patients (73%) presented with moderate-to-severe focal and diffuse abnormalities. Excess beta activity was identified in two STXBP1 patients (20%; Figure 2a). The most prevalent abnormality was a disrupted anterior-posterior (AP) rhythm gradient, observed in 47% of the cohort (7/15; 4/10 STXBP1, 3/5 SYT1) (Figures 2b, c). Absence of a posterior dominant rhythm (PDR) was observed exclusively in the STXBP1 group (4/10; 40%) (Figure 2c). Epileptiform discharges and spike-slow wave complexes were present in four patients (27%; 3/10 STXBP1, 1/5 SYT1) (Figure 2d). A burst-suppression pattern was observed in one SYT1 patient (20%). To summarize the overall extent and complexity of observed qualitative abnormalities, a ranked EEG abnormality severity score was constructed using an ordinal scale from 1 (normal) to 8 (most severe; scale 1–8; see Methods). Among *STXBP1* patients, severity ranks spanned the full range (1–8). Two patients had normal EEGs (Rank 1), four fell within the mild-to-moderate range (Ranks 2–5), and four displayed severe abnormalities (Ranks 6–8) driven by the co-occurrence of multiple high-weight abnormalities (e.g., slow PDR combined with epileptiform discharges). In contrast, the *SYT1* group showed a polarized profile: two patients had normal EEGs (Rank 1), while three scored in the moderate-to-severe range (Ranks 5–8), including one patient with burst suppression (Table 2). Our results indicate the presence of qualitative EEG abnormalities as well as provide a quantitative criterion for severity ranking across the cohort.

### Multidomain clinical PCA identifies components for functional deficits and qualitative EEG abnormalities

**Figure 3:**
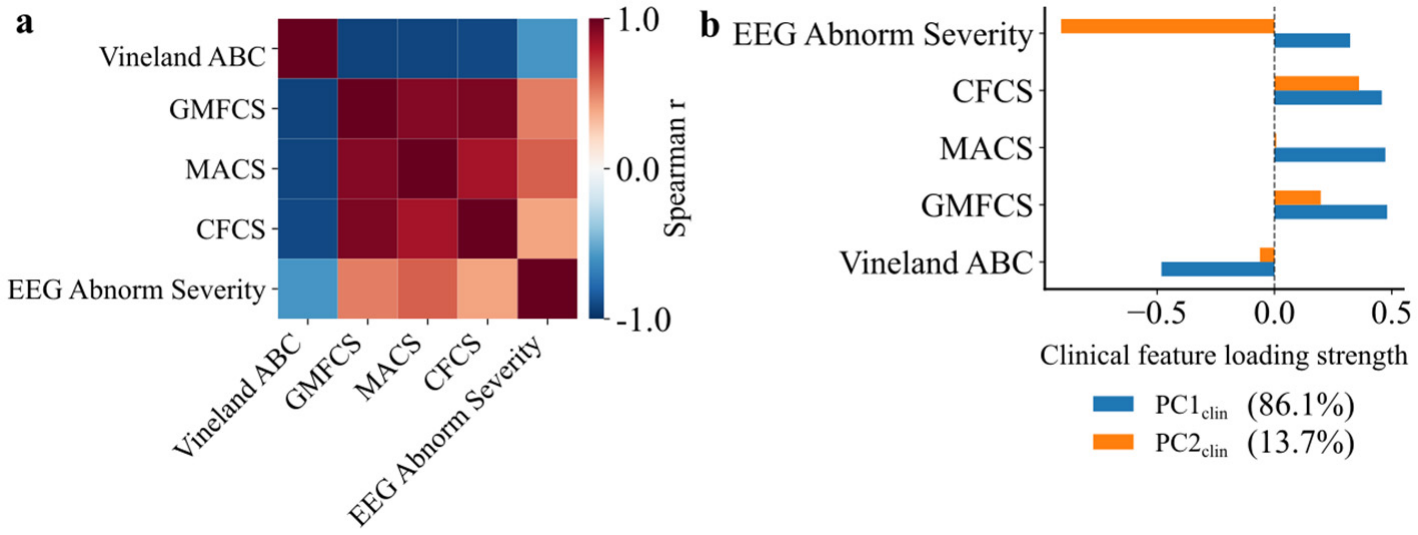
Clinical severity components reveal dissociation between functional impairment and EEG abnormalities. **(a)** Spearman correlation matrix showing the moderate to strong associations between different clinical severity indices across all patients (*n* = 15). The clinical indices include Vineland Adaptive Behavioral Composite score (Vineland ABC), Gross Motor Functional Classification System (GMFCS), Manual Ability Classification System (MACS), Communication Function Classification System (CFCS), and EEG abnormality-based severity ranking (EEG Abnorm Severity). **(b)** Loadings of each clinical index on the first two principal components (PC1_clin_, PC2_clin_) derived from PCA, along with percentage of variance explained. PC1_clin_ reflects shared variance across adaptive and functional impairments, PC2_clin_ captures variance dominated by EEG abnormality severity.

To systematically investigate whether clinical indices align along common or dissociable components of severity, we applied PCA to the ordinal EEG abnormality severity scores, Vineland ABC, GMFCS, MACS, and CFCS of the patient cohort. We observed an overall KMO score of 0.83 and χ^2^ = 62.35 (p_Bartlett_ < 0.05) indicating suitability for downstream PCA analysis. The first two principal components accounted for 95.3% of the total variance across patients (Figure 3). The first principal component (81.6%, PC1_clin_) emerged as a global severity component, with positive loadings from GMFCS, MACS, CFCS, EEG abnormality severity and a negative loading from Vineland ABC. Consistent with its scoring direction: higher Vineland scores indicate better adaptive functioning, so patients with higher PC1_clin_ scores showed more severe motor, manual, and communication impairments, more severe EEG abnormalities, and lower adaptive function (Figures 3a, b). The second principal component (13.7%, PC2_clin_) was dominated by a negative contribution from EEG abnormality scores and a moderate positive loading from CFCS, with minimal contributions from other indices (Figure 3b).

PC2_clin_ axis was dominated by an opposite-signed contribution from qualitative EEG abnormality severity and CFCS, with minimal contributions from the remaining clinical indices. Accordingly, patients with lower PC2_clin_ scores tended to coincide with higher qualitative EEG abnormality ranks alongside comparatively better functional communication. Together, PCA revealed two dimensions of clinical severity in SNAREopathies; one driven by global functional and adaptive deficits, and the other by the relative imbalance between electrophysiological abnormalities and communication impairments. This structure emphasizes the multidimensional clinical architecture of SNAREopathies and provides a compact framework to interpret patient variability across domains.

### Normative spectro-spatial EEG biomarker space derived from typically developing children cohort

We determined the normative patterns of variance across qEEG features in the TDC cohort. For absolute power, the first five principal components (PC) cumulatively accounted for 74.4% of the variance across the TDC under EOR. PC1 (24.7% variance) reflected delta (δ) and theta (θ) power across the default mode, control, dorsal attention and limbic networks. PC2 (13.5%) captured variance in alpha (α), beta (β), and beta-gamma (β-γ) frequencies across the somatomotor and dorsal attention networks. PC3 (12.7%) highlighted contributions across all frequencies in the salience ventral attention, and across α and β bands in limbic networks. PC4 (11.9%) represented power variation across all frequencies in the visual network, and PC5 (11.62%) encompassed activity in δ, α, β, as well as β-γ across the default mode and control networks (see Supplementary Figure S3a for absolute power loadings for each PC). For relative power, the first PC cumulatively accounted for 86.5% variance. PC1 captured (28.6% variance) α relative power across all seven functional networks. PC2 accounted (20.1%) for β-γ activity across all networks. PC3 (15.9%) was dominated by θ activity. PC4 (15.8%) reflected β relative power, and PC5 (6%) was associated with the β-γ relative power in the dorsal attention and visual networks (see Supplementary Figure S3b for relative power loadings for each PC). TDC cohort showed global patterns of variation for absolute power while frequency specific patterns emerged for relative power. To establish normative patterns of temporal structure of qEEG signals (LRTC), we applied PCA on the DFA exponents of the TDC cohort. The first five principal components cumulatively accounted for 78.5% variance. PC1 (16.4%) represented β-γ LRTC across all seven functional networks. PC2 (16.2%) was associated with α LRTC, PC3 (18.9%) with θ, PC4 (16.3%) with β, and PC5 (10.8%) represented δ LRTC across all networks (see Supplementary Figure S3c for LRTC loadings for each PC). Overall, the normative qEEG biomarkers from typically developing children showed distinct organizational patterns: relative power and LRTC primarily structure along frequency-specific axes, while absolute power exhibits more diffuse spectro-spatial patterns involving combinations of frequency bands and functional networks. The normative space can therefore serve as a reference framework for identifying physiological deviations in subsequent analyses.

### Normative qEEG reference space captures changes in neurophysiological states

**Figure 4:**
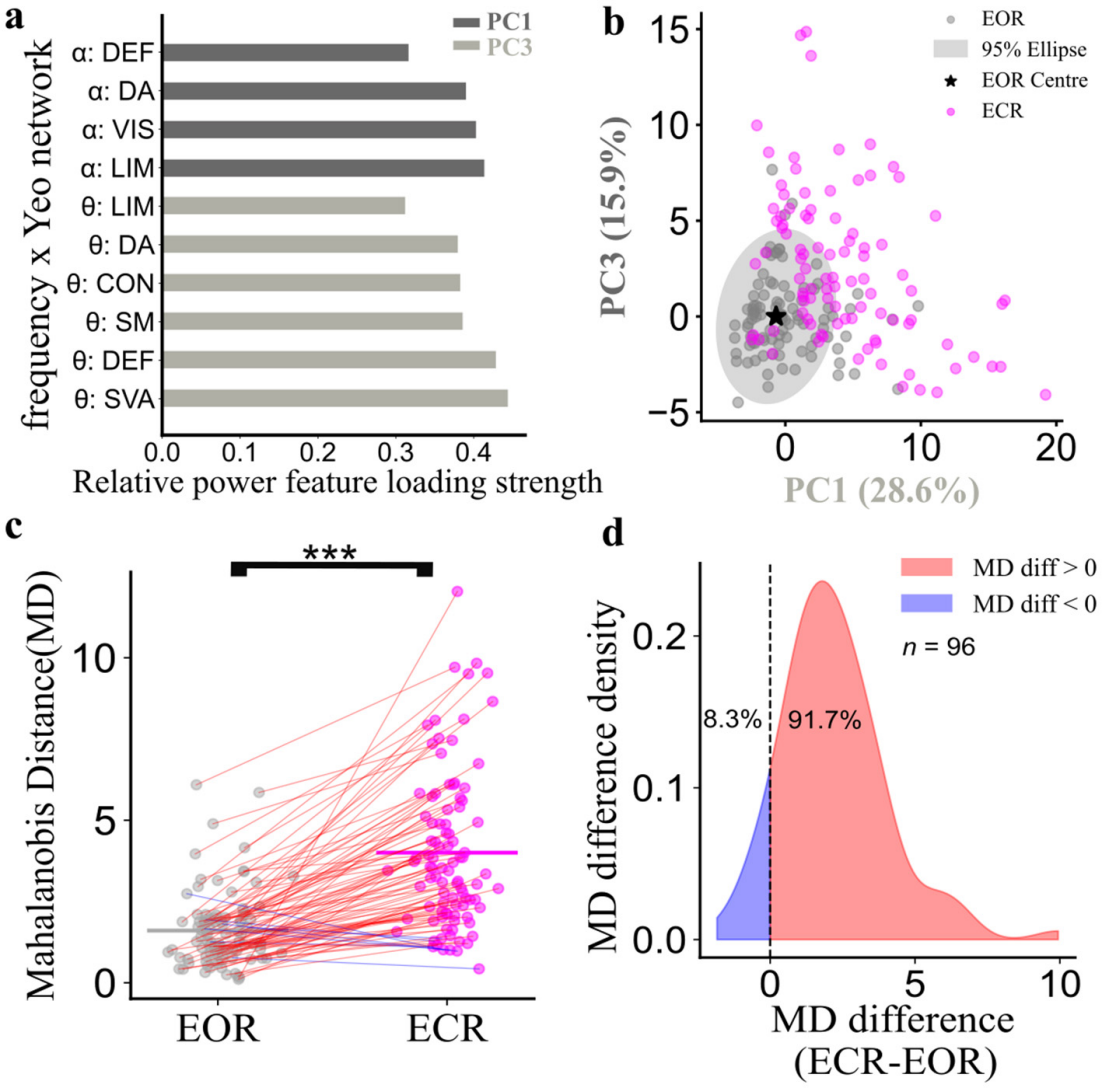
Mahalanobis distances capture spectro-spatial power differences between eyes-open and -closed states. **(a)** Relative power features across principal component 1 (PC1) and 3 (PC3) for the reference cohort [typically developing children (TDC) in eyes-open rest (EOR), *n* = 96]. Each feature represents the loading strength of relative power for a combination of frequency bands (α = alpha band ranging from 8.2 to 13.3 Hz, θ = theta band ranging from 4 to 8.2 Hz) and functional Yeo networks (DEF, Default Mode; DA, Dorsal Attention; VIS, Visual; LIM, Limbic; CON, Control; SM, Somatomotor; SVA, Salience Ventral Attention). **(b)** Projection space of PC1 and PC3 (percentage variance explained). Gray dots represent the TDC under EOR (*n* = 96), magenta dots represent the identical TDC under eyes-closed rest (ECR) state, black star represents the center of the TDC EOR relative power activity. Mahalanobis distances were computed for each TDC, under both EOR and ECR conditions as the distance between the reference EOR centre and individual position in the PC1–PC3 projection space. **(c)** TDC Mahalanobis distances (MD) under EOR and ECR conditions. *p*-values are reported after FDR adjustment (***p < 0.0001). **(d)** Differences between MD ECR and MD EOR for TDC. *p*-values are reported after FDR adjustment.

Next, we wanted to evaluate the sensitivity of the normative EEG space constructed from TDC under eyes open rest condition to changes in physiological states. qEEG activity under eyes-closed resting-state (ECR) is associated with characteristic increases in alpha and theta activity, providing a physiologically grounded contrast to assess the robustness of the reference space (Barry et al., 2007; Wei et al., 2018; Krukow et al., 2024). Principal components derived from the EOR TDC cohort were used as a fixed basis for projection. Both EOR and ECR data were projected into EOR reference space, and Mahalanobis distances from the normative EOR centre were computed for each condition and biomarker (TDC, *n* = 96 observations for ECR and EOR). For relative power, the loadings of key components (Figure 4a) revealed the spectral organization: PC1 captured alpha-band activity, while PC3 reflected theta-band contributions across cortical networks. The two-dimensional projection space defined by PC1 and PC3 (Figure 4b) showed that TDC EOR points were tightly clustered, whereas ECR points shifted toward higher positive values on both axes. This pattern indicates elevated alpha and theta power under eyes-closed conditions. Importantly, MD proved to be a sensitive measure with 92.7% of TDC participants increasing the distance to the reference EOR centre when resting with eyes closed (Wilcoxon *r* = 0.82, 95% C.I. = 0.77–0.83, p_FDR_ < 0.001, Figures 4c, d; see Supplementary Figure S4 for extended results). For absolute power, we observed weakly significant differences in the PC1–PC4 projection space (Wilcoxon r = 0.3, 95% C.I. = 0.11–0.48, p_FDR_ < 0.05; see Supplementary Figure S5). No significant differences were observed for MD in the LRTC projection space, under the two different states for TDC.

### SNAREopathy deviations from normative spectral power links to neurodevelopmental delay and functional impairment

To identify deviations from normative brain dynamics, we projected patient absolute and relative power features into the principal component combinations derived from the same set of features for TDC EOR (e.g., PC1–PC2, PC1–PC3; see Supplementary Tables S1, S2 for extended results and statistics).

#### Absolute power deviations from the norm

**Figure 5:**
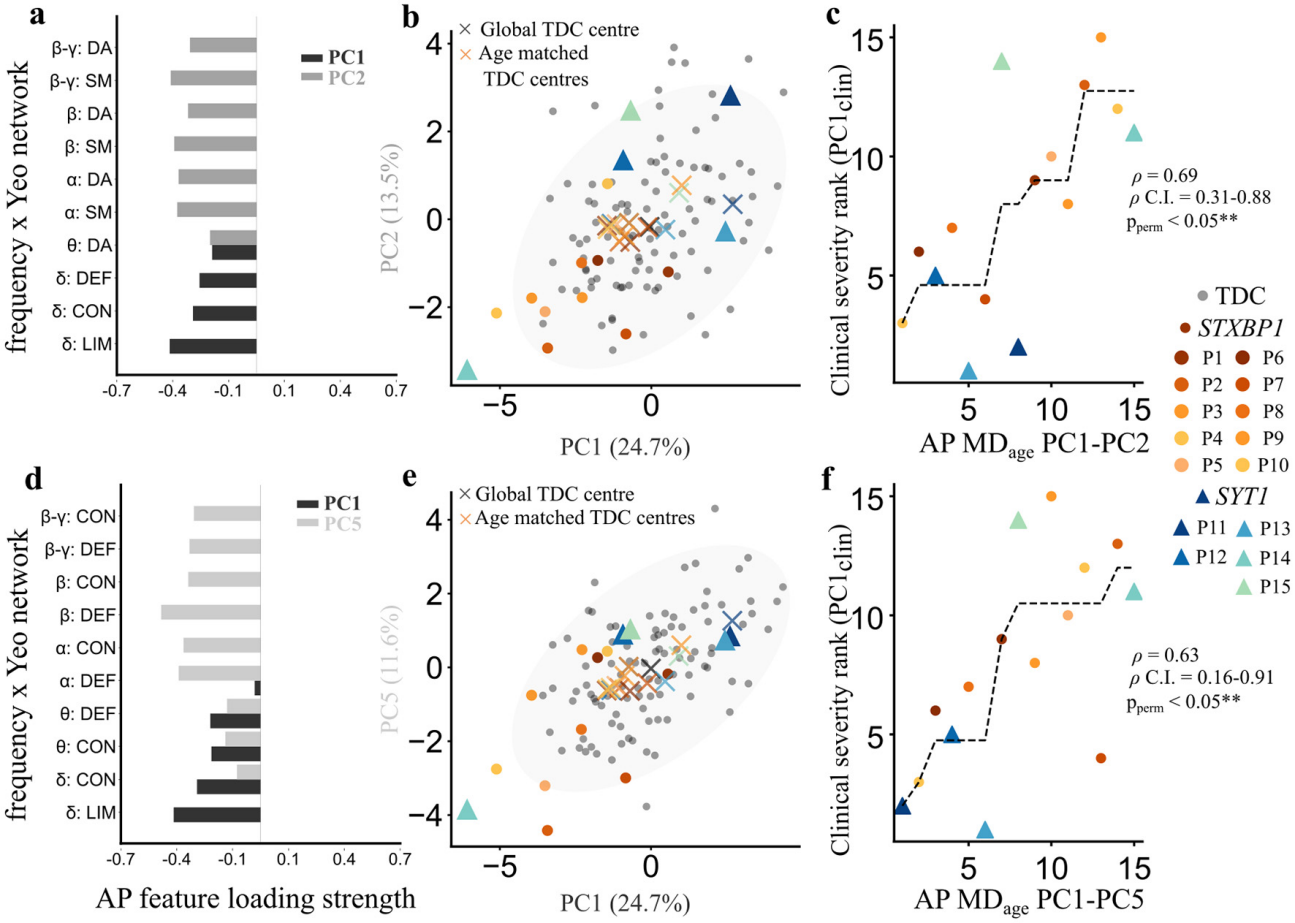
SNAREopathy deviations from the age-matched normative absolute-power patterns relate to the primary clinical severity axis. **(a)** Absolute power (AP) features across principal component 1 (PC1) and 2 (PC2) for the reference TDC cohort in eyes-open rest (EOR). Each feature represents the loading strength of absolute power for a combination of frequency band (see Figure 4 legend for feature details). **(b)** Projection space of TDC absolute power PC1 and PC2 components (percentage variance explained by each component). Gray points indicate position of individual TDC in EOR state. Colored points (*STXBP1, n* = 10) and triangles (*SYT1, n* = 5) indicate position of patients in the projection space. Gray cross indicates robust center of the projection space based on entire TDC cohort (Global TDC centre). Colored crosses indicate patient-specific age-matched TDC centres (Age-matched TDC centres). **(c)** Spearman rank correlation between clinical ranks for patients (*n* = 15) along clinical PC1 (y axis: PC1_clin_) and ranked patient age-matched distances (MD_age_) from PC1–PC2 (x axis: AP MD_age_ PC1–PC2). ρ denotes Spearman correlation coefficient, and ρ_perm_ denotes the *p*-value obtained after 15,000 permutation tests. Black dotted line indicates an isotonic regression curve. **(d)** AP features across principal component 1 (PC1) and 5 (PC5) for the TDC in EOR state (*n* = 96). Each feature represents the loading strength of absolute power as in **(a)**. **(e)** Projection space of TDC absolute power PC1 and PC5 components. **(f)** Spearman rank correlation between clinical ranks for patients along clinical PC1 (y axis: PC1_clin_) and ranked patient age-matched distances (MD_age_) from PC1–PC5 (x axis: AP MD_age_ PC1–PC5).

Among all the tested combinations, patient deviations (MD_age_) from the normative absolute power PC1–PC2 (Figure 5a) and PC1–PC5 combinations (Figure 5d) showed significant associations with PC1_clin_. Patient projections into these combinations (Figures 5b, e) revealed a predominance of negative scores along PC1, indicative of elevated absolute power in delta (δ) and theta (θ) bands across the default mode, control, dorsal attention and limbic networks, in patients relative to the normative distribution. We quantified the MD_age_ of each patient from their age-matched TDC. These distances were positively correlated with the principal clinical severity component spanning motor, communication, adaptive impairments, and EEG abnormalities [ρ = 0.69, 95% C.I. = 0.31–0.88, *p*_*perm*_ < 0.05 (PC1–PC2); ρ = 0.63, 95% C.I. = 0.16–0.91, *p*_*perm*_ < 0.05 for PC1–PC5; Figures 5c, f]. In contrast, patient deviations from the entire TDC cohort (MD_global_) were not significantly associated with clinical severity. PC1 of absolute power was a common component in both combinations, underscoring the role of low-frequency power across cortical networks as a unifying electrophysiological correlate of clinical severity. Age-adjusted deviations in absolute power may serve as a sensitive marker of multidimensional clinical burden in SNAREopathies.

#### Relative power deviations from the norm

**Figure 6:**
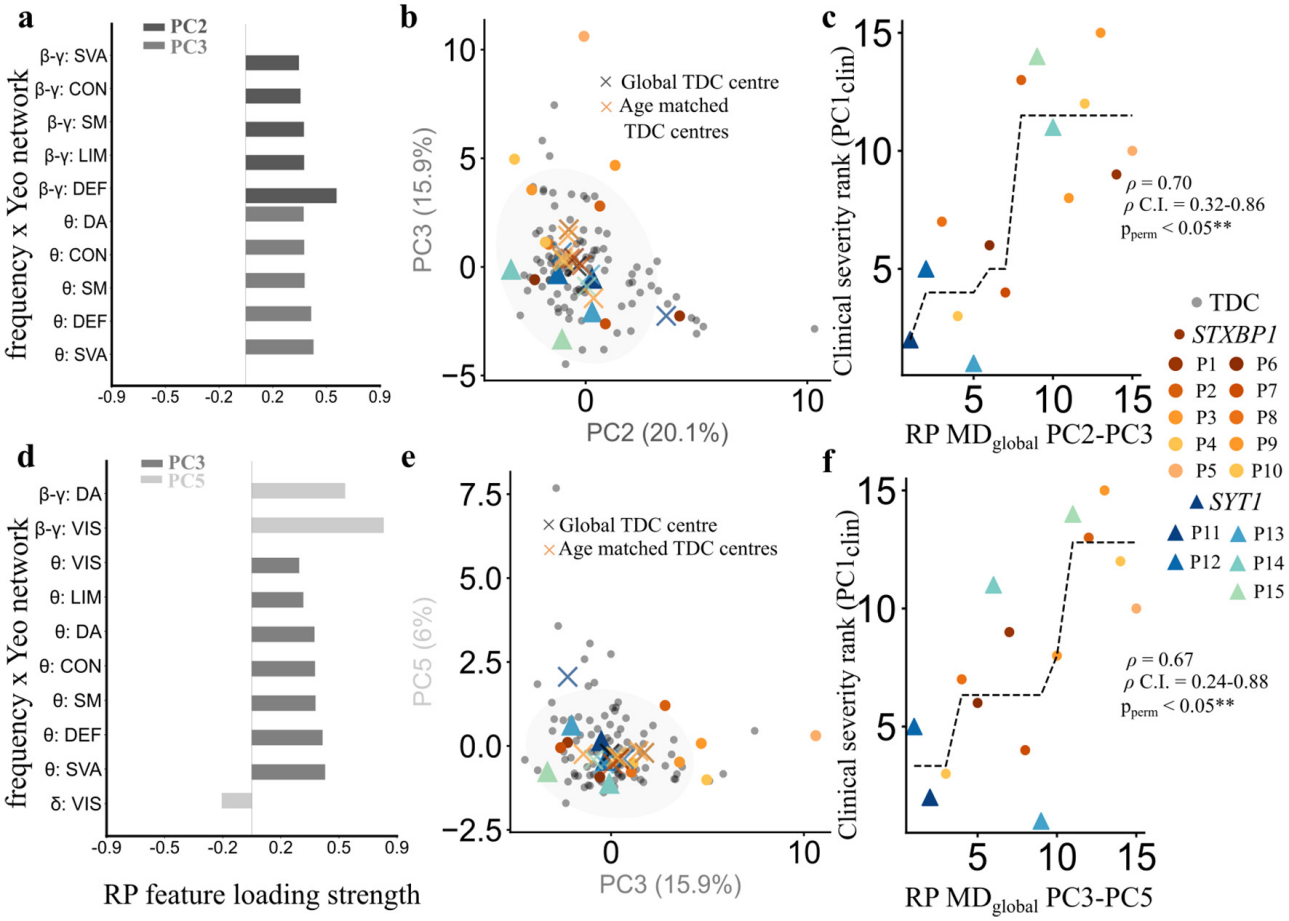
SNAREopathy deviations from the normative relative power patterns relates to the primary clinical severity axis. **(a)** Relative power (RP) features across principal component 2 (PC2) and 3 (PC3) for the reference TDC cohort in eyes-open rest. Each feature represents the loading strength of relative power for a combination of frequency band (as in Figure 4). **(b)** Projection space of TDC relative power PC1 and PC2 components (percentage variance explained by each component; see Figure 4b legend). **(c)** Spearman rank correlation between clinical ranks for patients along clinical PC1 (y axis: PC1_clin_) and ranked patient global distances (MD_global_) from PC2–PC3 (x axis: RP MD_global_ PC2–PC3). ρ denotes Spearman correlation coefficient, and ρ_perm_ denotes the *p*-value obtained after 15,000 permutation tests. Black dotted line indicates an isotonic regression curve. **(d)** RP features across principal component 3 (PC3) and 5 (PC5) for the TDC in EOR state. **(e)** Projection space of TDC relative power PC3 and PC5 components **(f)** Spearman rank correlation between clinical ranks for patients along clinical PC1 (y axis: PC1_clin_) and ranked patient global distances (MD_global_) from PC3–PC5 (x axis: RP MD_global_ PC3–PC5).

Among all the test combinations, patient deviations (MD_global_) from normative relative power PC2–PC3 (Figure 6a) and PC3-PC5 (Figure 6d) combinations showed significant associations with principal clinical severity axis (PC1_clin_). Patient projections into these combinations revealed a predominance of positive scores along PC3, indicative of elevated θ relative power in certain patients relative to the normative distribution (Figures 6b, e). Patient distances from the global TDC centre were positively correlated with the PC1_clin_ spanning motor, communication, adaptive impairments, and EEG abnormalities (ρ = 0.70, 95% C.I. = 0.32–0.86, *p*_*perm*_ < 0.05 for PC2–PC3; ρ = 0.67, 95% C.I. = 0.24–0.88, *p*_*perm*_ < 0.05 for PC3–PC5; Figures 6c, f). In contrast, MD_age_ computed from the full TDC cohort were not significantly associated with clinical severity. PC3 of relative power was common in both combinations underscoring the central role of low frequency θ power across cortical networks as a unifying electrophysiological correlate of global clinical severity.

### LRTC links to communication deficits and qualitative EEG abnormalities

**Figure 7:**
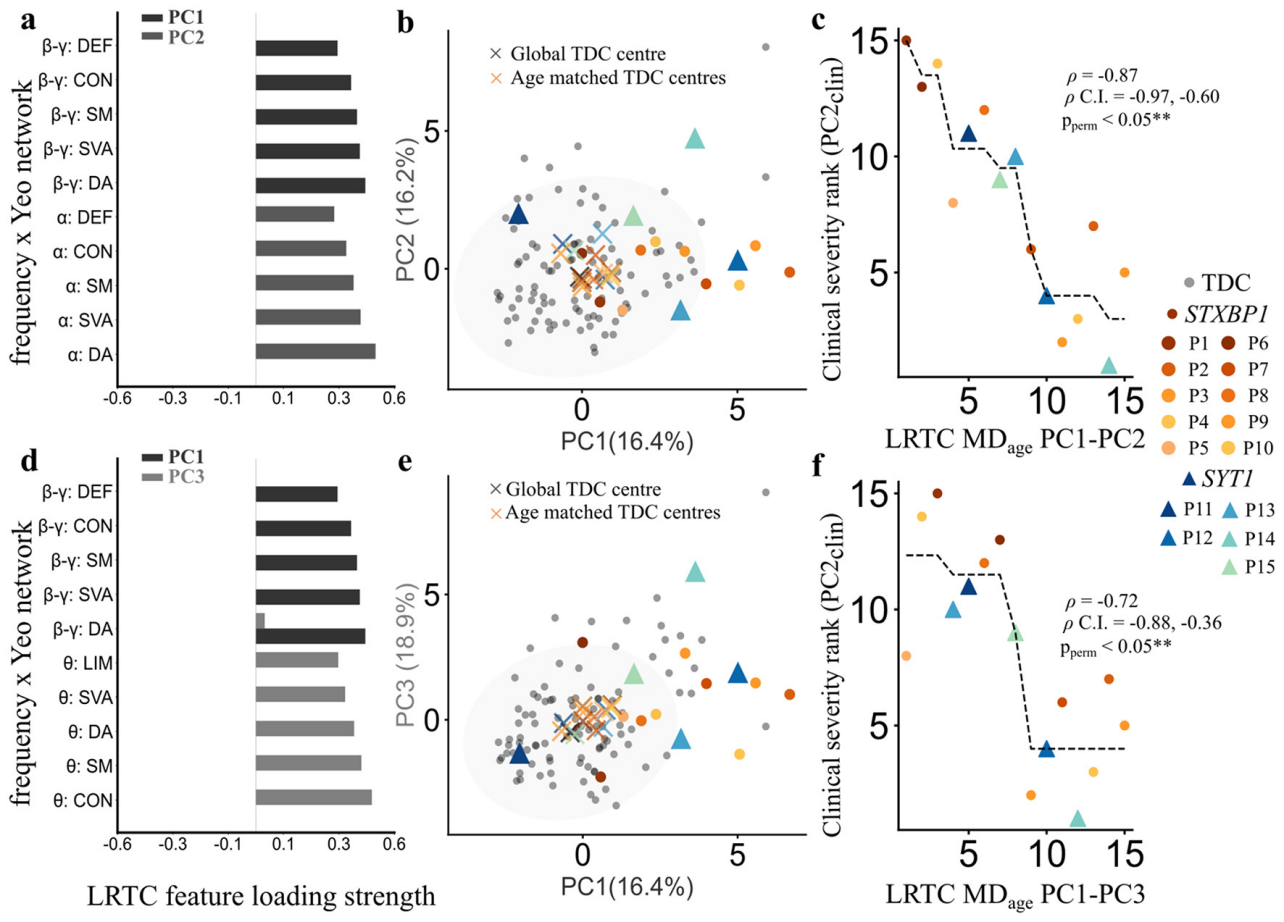
SNAREopathy deviations from the normative LRTC patterns relates to the secondary clinical severity component. **(a)** Long**-**range temporal correlation (LRTC) features across principal component 1 (PC1) and 2 (PC2) for the reference typically developing children (TDC) in eyes-open rest (EOR) (*n* = 96). **(b)** Projection space of TDC LRTC PC1 and PC2 components (percentage variance explained by each component). **(c)** Spearman rank correlation between clinical ranks for patients (*n* = 15) along clinical PC2 (y axis: PC2_clin_) and ranked patient age-matched distances [(MD_age_) from PC1–PC2 (x axis: LRTC MD_age_ PC1–PC2)]. ρ denotes Spearman correlation coefficient, ρ C.I. denotes the estimated confidence interval of ρ obtained after 15,000 bootstraps, and ρ_perm_ denotes the *p* value obtained after 15,000 permutation tests. Black dotted line indicates an isotonic regression curve. **(d)** LRTC features across principal component 1 (PC1) and 3 (PC3) for the TDC in EOR state (*n* = 96). Each feature represents the positive loading strength of absolute power for a combination of frequency band and functional Yeo network. **(e)** Projection space of TDC LRTC PC1 and PC3 components (percentage variance explained by each component). **(f)** Spearman rank correlation between clinical ranks for patients (*n* = 15) along clinical PC2 (y axis: PC2_clin_) and ranked patient age-matched distances (MD_age_) from PC1–PC3 (x axis: LRTC MD_age_ PC1–PC3).

To identify deviations from normative brain dynamics, we projected patient LRTC features into the principal component combinations derived from the LRTC features for TDC EOR (e.g., PC1–PC2, PC1–PC3). Among all the tested combinations, patient deviations (MD_age_) from normative LRTC PC1–PC2 (Figure 7a) and PC1–PC3 (Figure 7d) combinations showed significant associations with secondary clinical severity axis (PC2_clin_). Patient projections into these combinations (Figures 7b, e) revealed a predominance of positive scores, indicative of elevated LRTC in θ,α, and β-γ bands relative to the normative distribution. MD_age_ were negatively correlated with the secondary clinical severity component spanning communication, and EEG abnormalities (ρ = −0.87, 95% C.I. = −0.97,−0.60, *p*_*perm*_ < 0.05 for PC1–PC2; ρ = −0.72, 95% C.I. = −0.88, −0.36, *p*_*perm*_ < 0.05 for PC1–PC3; Figures 7c, f). In contrast, global distances computed from the entire TDC cohort (MD_global_) were not significantly associated with clinical severity. Together, these findings show that patient deviations in LRTC are specifically captured by age-referenced normative distances and align with clinical variation in communication and EEG abnormalities, highlighting the sensitivity of this measure to patient-relevant dysfunction.

## Discussion

In this study, we analyzed the complex organization of pediatric qEEG patterns through multivariate approaches integrating qualitative and quantitative analyses, revealing critical insights into monogenic neurodevelopmental phenotypes of SNAREopathies. This integration not only highlights the coordinated interplay between different frequency bands and brain networks but also underscores the importance of integrating these elements to understand the normative developmental EEG landscape. These findings contribute to a deeper understanding of how brain maturation influences cognitive and behavioral outcomes, paving the way for more effective assessments in clinical settings.

We established a normative spectro-spatial qEEG reference space using EOR from TDC cohort, providing a baseline model of EEG organization. This low-dimensional space derived via sparse PCA providing comprehensive yet compact representations of multivariate EEG organization. The slow-wave dominance (δ and θ power across all cortical networks) in absolute power PC1 (24.7%) is consistent with childhood excess of slow rhythms due to synaptic overabundance and protracted maturation of executive function (Whitford et al., 2007; Kang et al., 2024; Ünsal et al., 2024). PC2 (13.5%), characterized by α, β and β-γ power, highlights the early development of sensorimotor systems and attentional processing, with increased alpha synchrony as children grow (Ünsal et al., 2024; Wilkinson et al., 2024). PC3 (12.7%) integrated broad power in salience attention and α-β in limbic networks, indicating flexible engagement of circuits during emotional and cognitive control development (Menon and Uddin, 2010). PC4 (11.9%), a visual network component spanning δ through γ, underscores early functional maturity of occipital rhythms and the tendency for visual region power to covary across bands in childhood (Fair et al., 2007; Ünsal et al., 2024). PC5 (11.6%), mixing δ/α/β/β-γ in default mode and control networks, a transitional state of network integration, suggesting less segregation between systems typical of adult modularity (Fair et al., 2007; Ünsal et al., 2024).

Relative power components were predominantly global frequency specific modes rather than spatially localized network patterns. PC1 (28.6%) indicated a shift toward alpha dominance, while PC2 (20.1%) captured increases in beta-gamma power linked to cortical excitation and arousal regulation (Başar, 2012; Orekhova et al., 2018; Candelaria-Cook et al., 2022). PC3 (15.9%) captured global theta power, which in resting-state EEG is consistently associated with reduced vigilance/sleepiness and drowsiness-related state fluctuations, and can also covary with mind-wandering dynamics depending on context (Snipes et al., 2022; Strijbis et al., 2022; Tan et al., 2024). PC4 (15.8%) represented beta rhythms, plausibly reflecting sensorimotor idling and early frontal attentional readiness (Trevarrow et al., 2019; Barone and Rossiter, 2021; Candelaria-Cook et al., 2022; Amande et al., 2024). PC5 (6.0%) highlighted elevated beta–gamma power specifically in the dorsal attention and visual networks, suggesting advanced network specialization in older or more engaged children. The LRTC components capture 78.5% of variance (PC1: 16.4%, PC2: 16.2%, PC3: 18.9%, PC4: 16.3%, PC5: 10.8%), emphasizing the persistence of oscillatory amplitude fluctuations in normative development. Beta-gamma LRTC reflecting stable excitatory–inhibitory tuning and critical-state neural dynamics associated with cognitive readiness and robust cortical processing, while alpha-band LRTC reflects individual differences in persistence of alpha oscillations (Linkenkaer-Hansen et al., 2001; Berthouze et al., 2010; Hardstone et al., 2012). The theta and delta components further highlight developmental variability in cognitive engagement and drowsiness, indicating that distinct oscillatory circuits evolve across different timescales (Linkenkaer-Hansen et al., 2005; Herzog et al., 2021; Tan et al., 2024).

We validated the EOR-derived normative space against the physiological contrast of eyes closed resting state (ECR). ECR is a physiological state known to enhance alpha power and modulate low-frequency activity in typically developing children and adults (Barry et al., 2007; Wang et al., 2022; Krukow et al., 2024; Clarke et al., 2001; Barry and De Blasio, 2017). Projecting EOR and ECR recordings from the same cohort into the fixed EOR basis revealed state-dependent shifts, with ECR showing increased distances along alpha- and theta-dominated relative power components and a significant rise in MD for the majority of TDC. The observed shift indicates that the components capture canonical state-dependent deviations in neural dynamics. The normative relative power PC1 reflects strongly alpha-loaded component; hence a part of the increase in MD under ECR is expected to reflect deviations in alpha organization. However, MD is computed over the joint multivariate distribution of all components, and in our data the ECR–EOR contrast was not confined to a single alpha component. Significant state-related shifts were observed across several PC pairs (Supplementary Figure S4). The increased distances in the relative power space therefore reflect a genuine multivariate physiological separation between conditions rather than a narrow sensitivity to alpha dominance alone. The absence of significant differences in MD for LRTC between EOR and ECR does not imply that temporal scaling of EEG activity is invariant across states or MD lacks sensitivity. Prior work indicates that LRTC differ between eyes-open and eyes-closed conditions but vary across bands and regions (Linkenkaer-Hansen et al., 2001; Nikulin and Brismar, 2005; Gao et al., 2008). In our normative space, LRTC components were frequency-specific, whereas the physiological contrast between EOR and ECR increase in alpha (and to a lesser extent theta) power rather than a consistent, large-magnitude shift in temporal correlations. Consequently, the state-related changes in LRTC were distributed across several components and too small, relative to within-state variability, to produce robust increases in MD. Collectively, these findings emphasize that pediatric qEEG organization is defined by coordinated patterns across brain networks and frequencies, evolving alongside structural and functional maturation. This framework enables patient features to be quantified as multivariate deviations from typical development.

Having established this normative qEEG space from TDC, we next characterized the clinical and electrophysiological profiles of the SNAREopathy cohort (Tables 1, 2). Visual inspection of the qualitative EEG abnormalities revealed that nearly 75% of patients had EEG abnormalities, highlighting significant neurophysiological disruption in SNAREopathies. Previous studies found that about 89% of *STXBP1* patients experience seizures or abnormal EEG patterns (Xian et al., 2022). Baker et al. noted certain *SYT1* patients had epileptiform discharges without seizures, suggesting underlying epileptic activity (Baker et al., 2018). In our findings, two of the five *SYT1* patients had normal EEGs, while the others exhibited moderate to severe abnormalities, confirming that *SYT1* mutations could lead to notable electrographic disturbances, though epilepsy is less common compared to *STXBP1*-related disorders (Cesaroni et al., 2023). Both genotypes showcased qualitative EEG abnormalities consistent with diffuse encephalopathy, particularly a disrupted anterior–posterior rhythm gradient in seven patients. Normally, pediatric EEG should display a dominant alpha rhythm in the posterior region, which diminishes toward the front; disruption in this pattern indicates cortical disorganization (Kurth et al., 2010; Chu et al., 2014; Louis et al., 2016; Amin et al., 2023; Emmady et al., 2025). Notably, four *STXBP1* patients displayed slow or absent posterior rhythm, correlating with severe cognitive impairment (Deprez et al., 2010; Valdés-Hernández et al., 2010; Lanoue et al., 2019; Xian et al., 2022). We ranked EEG abnormality severity from 1 (normal) to 8 (most severe). *STXBP1* patients covered the entire range, while *SYT1* patients were mostly in the lower half, suggesting that *STXBP1* generally worsens EEG abnormalities, whereas *SYT1* often shows milder issues or normal activity (Baker et al., 2018). Importantly, a normal EEG does not imply normal neurological function; two *SYT1* patients with normal EEGs faced developmental delays (Xian et al., 2022). Our findings reinforce that developmental delay and multidomain functional impairments are the norm in SNAREopathies, underscoring the need for comprehensive functional assessments rather than single-domain evaluations (Deprez et al., 2010; Baker et al., 2018; Verhage and Sørensen, 2020; Riggs et al., 2022; Xian et al., 2022; Cesaroni et al., 2023; Park et al., 2023).

We applied PCA to complex phenotypic variability into a compact framework, revealing two orthogonal axes of clinical severity that captured ~95% of the variance. The first principal component (PC1_clin_) represented a global severity index with high patient scores corresponding to more severe motor disability, poorer hand function, impaired communication, EEG abnormalities, and lower adaptive ability. The finding aligns with the qualitative clinical description of most severe patient cases. Such a unidimensional severity axis can be supported by prior studies: for instance, Xian et al. found that earlier and more intractable epilepsy (a surrogate of high neurological severity) was associated with worse gross motor and language outcomes in *STXBP1* (Xian et al., 2022). Park et al. (2023) demonstrated a genotype–phenotype correlation in *SYT1* disorder, where variants causing greater dysfunction in synaptic release yielded patients with more severe motor and communication deficits. In the context of SNAREopathies, PC1_clin_ may serve as an integrated severity metric, summarizing the patient's global functional level in one dimension. This is an important methodological strength since many monogenic neurodevelopmental disorders lack a single definitive clinical endpoint. An integrated measure could be useful in capturing the complex clinical architecture and burden of the disorder. The second principal component (PC2_clin_) defined a distinct severity axis that separated patients-based EEG abnormalities and communication function. Patients with high PC2_clin_ scores demonstrated poor communication but milder EEG abnormalities, and vice-versa. Prior studies have reported on the dissociation between electrophysiological severity and clinical function. In Dravet syndrome, children experience very frequent or severe seizures with a relatively normal background interictal EEG, while in *STXBP1* syndrome, epileptic activity and cognitive outcome represent at least partly independent dimensions of the phenotype (Stamberger et al., 2016; Palmer et al., 2021). This pattern should not be interpreted as a causal inverse relationship; rather, it indicates that electrophysiological abnormality burden and functional communication impairments did not uniformly co-vary across individuals in this small, heterogeneous sample. This motivates mechanistic hypotheses (e.g., differing developmental trajectories, compensatory network dynamics, or measurement-timescale differences) that require validation in larger and longitudinal datasets. Overall, the findings emphasize that the clinical profile of SNAREopathies is more than a simple sum of deficits, offering a tool to deconstruct the complex phenotypic presentations (Palmer et al., 2021).

Our results demonstrate that patients with SNAREopathies show pronounced low-frequency absolute power that tracks overall clinical impairment. In the normative PCA space, patients showed robust deviations along a delta–theta dominated absolute power component, and greater age-adjusted deviation within this component (higher MD_age_ on the corresponding principal component) was positively associated with the PC1_clin_. This aligns with observations in *STXBP1*-related encephalopathy, where a predominant slow-wave (delta and theta) activity is a feature that correlates with more severe motor and cognitive deficits. Similarly, *SYT1* patients exhibit bursts of high-amplitude, synchronous slow oscillations, reflecting a fundamental disruption of normal cortical rhythmogenesis. This converges with studies in genetic epilepsies where spectral power is markedly shifted toward delta and spectral slowing serves as a biomarker of disease burden (Frohlich et al., 2019; Houtman et al., 2021; Cossu et al., 2024; Galer et al., 2025). Slow-wave absolute power is strongly age-dependent, with high delta and theta power in early childhood that declines steeply across later childhood and adolescence alongside a redistribution of power toward faster rhythms (Chu et al., 2014; Candelaria-Cook et al., 2022; Hill et al., 2022). MD computed relative to a single global normative center (MD_global_) pools across the developmental gradient. Patients with elevated slow-wave power may appear relatively close to younger, physiologically typical children and thus not stand out as markedly abnormal in the global space. In contrast, MD_age_ quantifies how far an individual's absolute spectral profile deviates from the age-appropriate normative pattern, accounting for maturational changes and isolating disease-related excess slow-wave activity. We observed that the degree of deviation in relative power, particularly along the component loaded on increased theta power, correlated strongly with PC1_clin_. This mirrors findings in other severe neurodevelopmental conditions, often interpreted as a sign of diffuse cortical under arousal or immature network activity (Frohlich et al., 2019; Goodspeed et al., 2023; Ramautar et al., 2025). Taken together, excessive absolute delta–theta power and a relative shift toward theta-dominated activity within the normative space converge on low-frequency network hyperactivity as a qEEG signature of high clinical burden in SNAREopathies indexed by PC1_clin_. Our analysis also uncovered a relationship between deviations from LRTC and clinical severity along PC2_clin_. MD_age_ from normative LRTC PC1-PC2 and PC1-PC3 combinations were negatively correlated with PC2_clin_. This indicates that patients with the most pronounced LRTC deviations exhibited marked EEG abnormalities but relatively preserved communication abilities. Increased LRTC implies that neuronal oscillations have stronger autocorrelations, less random and more temporally structured over extended periods (Linkenkaer-Hansen et al., 2001, 2005; Hardstone et al., 2012). Mechanistically, these findings are consistent with the model in which synaptic dysfunction perturbs excitation-inhibition regulation and elicits homeostatic responses that reshape large-scale network dynamics (Chen et al., 2022). In this framework, compensatory regulation of excitatory and inhibitory gain can tune cortical activity toward a near-critical regime, which has been proposed to support wide dynamic range, efficient information processing, and flexible integration of inputs (Rocha et al., 2018; Ma et al., 2019; Zimmern, 2020; Fusca et al., 2022). Such homeostatically maintained near-critical dynamics provide a plausible explanation for how some patients may retain relatively preserved functional capacities, such as communication, even when visual EEG assessment indicates marked background abnormalities. At the same time, theoretical and experimental work shows that homeostatic plasticity can overshoot or become maladaptive such that attempts to stabilize firing rates or network activity lead to excessively persistent, rigid dynamics and secondary functional impairment (Fröhlich et al., 2008; Wu et al., 2020; Chen et al., 2022). Elevated LRTC likely reflects the net outcome of homeostatic regulation acting on already perturbed synaptic and circuit substrates, with effects that depend on developmental timing, circuit specificity, and external factors such as medication. The direction of the association, together with prior work in *STXBP1* showing that stronger beta-band LRTC coincide with relatively better communication, supports the interpretation that, at for a subset of patients, elevated LRTC reflects compensatory stabilization that co-occurs with qualitative EEG abnormalities rather than simply indexing more severe functional impairment (Houtman et al., 2021; Ramautar et al., 2025).

Seizure history did not significantly correlate with any qEEG biomarkers since a large majority of the patients did not have a history of seizures or are currently on antiepileptic medication (Supplementary Methods S1b). An important caveat is that anti-seizure medications and other psychoactive drugs can themselves modulate EEG rhythms, for example by enhancing beta activity, broadening background slowing, or altering LRTC properties. In small, heterogeneous cohorts, variability in medication type, dose, and duration likely introduces additional variance that may affect associations between intrinsic network abnormalities and clinical measures. Future studies with larger samples should stratify analyses by medication class better isolate disorder-specific qEEG signatures. Overall, the results reinforce the value of coupling multidomain clinical indices with qEEG biomarkers to better characterize the heterogeneous phenotypes within these rare neurodevelopmental syndromes.

A critical challenge in rare disease research is the statistical limitation imposed by small sample sizes, which often renders standard group-level comparisons underpowered and prone to Type II errors. A key methodological strength of the study is the use of multivariate normative comparisons to overcome this constraint. By establishing a robust reference space from an age-matched TDC cohort, we transformed the analysis from a comparison of two small group means to a quantification of individual patient deviations from a stable normative baseline. The resulting Mahalanobis distance serves as a composite metric of statistical abnormality that aggregates subtle deviations across multiple features, deviations that might be individually insignificant but collectively reflect a profound disruption of network physiology. While the normative PCA framework offers a powerful way to quantify EEG abnormalities, the quality of the normative model depends on the representative TDC cohort. Further validation is required in larger healthy EEG cohorts as well as modeling age trends continuously using age-regressed normative model and developmental EEG growth charts (Marquand et al., 2016; Candelaria-Cook et al., 2022; Rutherford et al., 2023; Zamanzadeh et al., 2025). Another consideration is that a large Mahalanobis distance, can result from one dramatically abnormal feature or a combination of mild abnormalities across many features. Future work should focus on the top contributing features to each patient's distance, reconstructing the EEG features from the PCA with largest errors.

The PCA across clinical scales serves as a descriptive and hypothesis-generating purpose rather than predictive modeling. We sought to summarize the covariance structure of clinical severity within the patient cohort to guide interpretation of qEEG deviations, not to derive generalizable factor loadings. Replication in larger patient cohorts is essential to confirm the stability of the clinical severity dimensions. Children with developmental and behavioral disabilities are hard to assess as adherence to instructions is difficult, resulting in suboptimal data recording and quality with excessive artifacts in EEG signals. Spectral power can reliably be estimated from recordings with length of > 20 s (Gasser et al., 1985; Salinsky et al., 1991), whereas minimal signal length for reliable estimation for DFA is around 100 s (Bruining et al., 2020; Houtman et al., 2021; Diachenko et al., 2024; Ramautar et al., 2025). Despite the efforts to match age and gender, the developmental changes occurring in the brain require realistic age specific head models for accurate source reconstruction (Céspedes-Villar et al., 2020). Individual head models can be derived from the subject's MRI, but obtaining MRI data from children with NDD is challenging. Advances in creating age-appropriate developmental MRI atlases specifically tailored for EEG source reconstruction should be explored (Richards et al., 2016).

In conclusion, the normative TDC EEG space forms a foundation for integrated electro-clinical characterization, providing an age-appropriate baseline against which the heterogeneous effects of rare genetic disorders can be quantified. By summarizing multivariate deviations in absolute and relative power, LRTC with MD, the framework yields qEEG biomarkers of global disease burden and of specific electro-clinical dissociations. Such metrics are well suited to serve as objective endpoints in early-phase trials for SNAREopathies, enabling stratification by baseline severity, detection of treatment-related normalization of network dynamics, and tracking of longitudinal response. More broadly, normative qEEG growth charts may facilitate personalized monitoring of brain health and treatment response across pediatric neurodevelopmental conditions, complementing traditional clinical scales that often lack sensitivity to subtle but clinically meaningful changes.

## Data Availability

Data will be accessible after completion of the BRAINMODEL study, which will publish the raw, processed, and analyzed data in long-term data archives. Datasets will be uploaded to the certified DANS EASY archive. The DANS EASY archive stores data in accordance with applicable Dutch law and uses clear copyright procedures: the depositor holds the right to the data. This archive is certified with the Data Seal of Approval and uses persistent identifiers, such as DOIs, to ensure data findability. The metadata fields in DANS EASY comply with the guidelines of the Dublin Core.
