# Supplementary material for: Dimensionality reduction of quantitative EEG and clinical profiles uncover associations with monogenic neurodevelopmental phenotypes in SNAREopathies
Source: Front Neurosci. 2026 Jan 27;19:1725623. doi: 10.3389/fnins.2025.1725623 (PMC12886349; doi:10.3389/fnins.2025.1725623)
Supplement: Supplementary file 1 [file Data_Sheet_1.docx]

# Supplementary Methods

1. *Exclusion of menstrual cycle as a confounder in EEG analysis*

The SNAREopathy patient cohort comprises six females, of which four are younger than 10 years of age. Menarche typically occurs between ages 10-16, with an average onset at approximately 12 years (Lacroix et al., 2025). By age 15, approximately 98% of females have undergone menarche. Therefore, pre-pubertal females in our cohort (under 10-11 years) may not even experience menstrual cycles. Before menarche, ovarian hormone fluctuations are minimal, making menstrual cycle effects on EEG irrelevant in this subgroup. For the remaining females in our cohort who may be post-menarchal, methodological challenges preclude reliable menstrual cycle phase determination (Mansfield and Emans, 1984; Klusmann et al., 2023). While studies conducted in women aged 18-40 years demonstrate that ovarian hormone fluctuations modulate resting-state EEG parameters (Becker et al., 1982; Brötzner et al., 2014), these findings have not been systematically replicated in pediatric or adolescent populations. This represents a recognized gap in the developmental EEG literature that would require novel methodological approaches specifically designed for irregular adolescent cycles to address in future research. Given that the majority of our female participants are pre-pubertal and the remaining post-menarchal adolescents would exhibit highly irregular and predominantly anovulatory cycles that do not permit reliable cycle phase determination, we determined that controlling for menstrual cycle phase was neither feasible nor necessary in our study population.

## *EEG Abnormalities Severity Grading System*

| **EEG Abnormalities** | **Severity Scale** | **Severity Grade** |
| --- | --- | --- |
| Normal EEG | No abnormalities | 1 |
| Focal abnormal beta activity | Low local | 2 |
| Epileptiform discharges | Moderate local | 3 |
| Diffuse abnormal beta activity | Moderate global | 4 |
| Slow PDR (<8Hz) | High global | 5 |
| Abnormal ADR-PDR gradient, frontal slowing | High global | 5 |
| Burst suppression patterns | High global | 5 |

## *Severity ranking of SNAREopathies: seizure history and severity ranking of SNAREopathies based on seizure profiles*

Patients with no history of seizures were ranked with the lowest severity score. Generalized onset seizure types were regarded as more severe than focal onset seizures. Within generalized seizures, seizures with motor symptoms were assigned a higher severity score compared to non-motor absence seizures. Among focal seizures, those with impaired awareness and motor symptoms were rated as more severe than focal aware non-motor seizures (Fisher et al., 2017). Identification and classification of patient seizure history was performed by a trained neurophysiologist based on the International League Against Epilepsy seizure classification framework (Fisher et al., 2017). Each patient’s epilepsy severity score was calculated as the cumulative total of all seizure types documented in their clinical history. Higher cumulative scores reflected greater epilepsy severity. A score of one indicated least severe, and a score of 20 indicated highest severity based on seizure history). Seizure history and profiles revealed developmental and epileptic encephalopathy in 8/15 patients with SNAREoapthies. Across the 8 patients, history of one or more seizure-types were classified based on the International League Against Epilepsy criteria for seizure onset type. Generalized tonic-clonic seizures with motor automatisms and generalized typical absence seizures were observed in 4/8 patients each (n*_STXBP1_* =3, *n_SYT1_* =1). Focal seizures with impaired awareness and motor automatisms were observed in 2/8 patients (n*_STXBP1_* =2, *n_SYT1_* =0) and focal seizures with intact awareness and motor automatisms were observed in 2/8 patients (n*_STXBP1_* =1, *n_SYT1_* =1). Focal autonomic with intact awareness was observed in 1/8 (n*_STXBP1_* =1, *n_SYT1_* =0). Focal emotional seizures with intact awareness were observed in 1/8 (n*_STXBP1_* =0, *n_SYT1_* =1) patients. Epilepsy history was absent in 7/15 (n*_STXBP1_* = 4, *n_SYT1_* =3 ) patients.

1. *EEG preprocessing pipeline*

Recordings were imported into EEGLAB format and bandpass-filtered between 1–45 Hz using a FIR-filter with a Hamming window. Bad channels were defined as electrodes with a minimum channel correlation of <0.85, flat in a time-window of >5s and high frequency noise greater than 4 standard deviations relative to its signal based on total channel population. Bad channels were interpolated using a spherical spline. Segments of data whose variances were larger than the threshold burst criterion 20 were removed using Artifact Subspace Reconstruction burst correction (Kothe and Makeig, 2013). The data was then manually inspected for movement related artifacts. Independent component analysis (ICA), using Infomax algorithm (Pion-Tonachini et al., 2019), in combination with *ICLabel* was used to remove components with eye and movement artifacts. Subsequently, a visual inspection of the data was performed to determine the presence of residual artifacts. For *STXBP1* syndrome, on average, 3.8 (± 2.7; range: 1-10) independent components were removed, 21 (±12.7; range = 5-40) bad electrodes were identified and interpolated. After preprocessing, the average signal duration was 263.9 s (± 97 s; range: 147.9- 442.6 sec). For *SYT1* syndrome, on average, 7 (± 4.1; range: 4-14) independent components were removed, 22.4 (±14.7; range = 10-47) bad electrodes were identified and interpolated. After preprocessing, the average signal duration was 195.8 sec (± 133.4 s; range: 114.7- 432.1 s). For the TDC cohort, on average, 4.4 (± 2.1; range: 2-12) independent components were removed, 21.8 (±11; range = 4-65) bad electrodes were identified and interpolated. After preprocessing, the average signal duration was 205.7 s (± 42.9 s; range: 94- 285.4 s).

## *Handedness as a confounder in EEG analysis*

Handedness is unlikely to influence our primary findings The analytical framework minimizes the influence of hemispheric asymmetries. All qEEG biomarkers (absolute power, relative power, and LRTC exponents) were computed at the level of bilateral functional networks by averaging estimates across left and right hemisphere parcels within each of the seven Yeo networks (see Supplementary method 7). Handedness effects on resting-state EEG are most pronounced for hemispheric alpha power asymmetry indices (e.g., left minus right frontal alpha), particularly at fronto-central electrode sites overlying premotor and motor cortex (Propper et al., 2012; Packheiser et al., 2020). Such lateralization effects would be diluted in our bilateral network-level estimates, which integrate activity across distributed cortical regions spanning both hemispheres within each functional network. Further, the Mahalanobis distance quantifies each patient's deviation from the multivariate normative distribution, which inherently captures the covariance structure of the TDC cohort; hence any systematic handedness-related variance would be represented within the normative covariance matrix against which patient deviations are computed. Finally, a recent large-scale study of children aged 9-10 years found that while handedness was associated with functional connectivity laterality in sensorimotor regions, no differences were observed in global network organization or brain morphometrics between left-, right-, and mixed-handed children (Tomasi and Volkow, 2024). This could suggest that handedness effects are predominantly local and lateralized rather than affecting the global, bilateral network-level measures employed in our study. We acknowledge that future studies would benefit from systematic handedness assessment to enable direct evaluation of its potential influence on qEEG biomarkers in SNAREopathies.

## *Schaefer atlas: Yeo network to patches mapping (Schaefer et al., 2018)*

| Parcel Name | Long Parcel Name | Yeo Network | Full Parcel Name |
| --- | --- | --- | --- |
| C_Cing1-LH | 7Networks_LH_Cont_Cing_1-lh | C | cingulate subdivision 1 |
| C_PFCl1-LH | 7Networks_LH_Cont_PFCl_1-lh | C | lateral prefrontal cortex subdivision 1 |
| C_Par1-LH | 7Networks_LH_Cont_Par_1-lh | C | parietal subdivision 1 |
| C_pCun1-LH | 7Networks_LH_Cont_pCun_1-lh | C | precuneus subdivision 1 |
| D_PFC1-LH | 7Networks_LH_Default_PFC_1-lh | D | prefrontal cortex subdivision 1 |
| D_PFC2-LH | 7Networks_LH_Default_PFC_2-lh | D | prefrontal cortex subdivision 2 |
| D_PFC3-LH | 7Networks_LH_Default_PFC_3-lh | D | prefrontal cortex subdivision 3 |
| D_PFC4-LH | 7Networks_LH_Default_PFC_4-lh | D | prefrontal cortex subdivision 4 |
| D_PFC5-LH | 7Networks_LH_Default_PFC_5-lh | D | prefrontal cortex subdivision 5 |
| D_PFC6-LH | 7Networks_LH_Default_PFC_6-lh | D | prefrontal cortex subdivision 6 |
| D_PFC7-LH | 7Networks_LH_Default_PFC_7-lh | D | prefrontal cortex subdivision 7 |
| D_Par1-LH | 7Networks_LH_Default_Par_1-lh | D | parietal subdivision 1 |
| D_Par2-LH | 7Networks_LH_Default_Par_2-lh | D | parietal subdivision 2 |
| D_Temp1-LH | 7Networks_LH_Default_Temp_1-lh | D | temporal subdivision 1 |
| D_Temp2-LH | 7Networks_LH_Default_Temp_2-lh | D | temporal subdivision 2 |
| D_pCunPCC1-LH | 7Networks_LH_Default_pCunPCC_1-lh | D | precuneus posterior cingulate cortex subdivision 1 |
| D_pCunPCC2-LH | 7Networks_LH_Default_pCunPCC_2-lh | D | precuneus posterior cingulate cortex subdivision 2 |
| DA_FEF1-LH | 7Networks_LH_DorsAttn_FEF_1-lh | DA | frontal eye fields subdivision 1 |
| DA_Post1-LH | 7Networks_LH_DorsAttn_Post_1-lh | DA | posterior subdivision 1 |
| DA_Post2-LH | 7Networks_LH_DorsAttn_Post_2-lh | DA | posterior subdivision 2 |
| DA_Post3-LH | 7Networks_LH_DorsAttn_Post_3-lh | DA | posterior subdivision 3 |
| DA_Post4-LH | 7Networks_LH_DorsAttn_Post_4-lh | DA | posterior subdivision 4 |
| DA_Post5-LH | 7Networks_LH_DorsAttn_Post_5-lh | DA | posterior subdivision 5 |
| DA_Post6-LH | 7Networks_LH_DorsAttn_Post_6-lh | DA | posterior subdivision 6 |
| DA_PrCv1-LH | 7Networks_LH_DorsAttn_PrCv_1-lh | DA | precentral ventral subdivision 1 |
| L_OFC1-LH | 7Networks_LH_Limbic_OFC_1-lh | L | orbital frontal cortex subdivision 1 |
| L_TempPole1-LH | 7Networks_LH_Limbic_TempPole_1-lh | L | temporal pole subdivision 1 |
| L_TempPole2-LH | 7Networks_LH_Limbic_TempPole_2-lh | L | temporal pole subdivision 2 |
| SVA_FOI1-LH | 7Networks_LH_SalVentAttn_FrOperIns_1-lh | SVA | frontal operculum and insula subdivision 1 |
| SVA_FOI2-LH | 7Networks_LH_SalVentAttn_FrOperIns_2-lh | SVA | frontal operculum and insula subdivision 2 |
| SVA_Med1-LH | 7Networks_LH_SalVentAttn_Med_1-lh | SVA | medial prefrontal cortex subdivision 1 |
| SVA_Med2-LH | 7Networks_LH_SalVentAttn_Med_2-lh | SVA | medial prefrontal cortex subdivision 2 |
| SVA_Med3-LH | 7Networks_LH_SalVentAttn_Med_3-lh | SVA | medial prefrontal cortex subdivision 3 |
| SVA_PFCl1-LH | 7Networks_LH_SalVentAttn_PFCl_1-lh | SVA | lateral prefrontal cortex subdivision 1 |
| SVA_PO1-LH | 7Networks_LH_SalVentAttn_ParOper_1-lh | SVA | parietal operculum subdivision 1 |
| SM1-LH | 7Networks_LH_SomMot_1-lh | SM | somatomotor subdivision 1 |
| SM2-LH | 7Networks_LH_SomMot_2-lh | SM | somatomotor subdivision 2 |
| SM3-LH | 7Networks_LH_SomMot_3-lh | SM | somatomotor subdivision 3 |
| SM4-LH | 7Networks_LH_SomMot_4-lh | SM | somatomotor subdivision 4 |
| SM5-LH | 7Networks_LH_SomMot_5-lh | SM | somatomotor subdivision 5 |
| SM6-LH | 7Networks_LH_SomMot_6-lh | SM | somatomotor subdivision 6 |
| V1-LH | 7Networks_LH_Vis_1-lh | V | visual subdivision 1 |
| V2-LH | 7Networks_LH_Vis_2-lh | V | visual subdivision 2 |
| V3-LH | 7Networks_LH_Vis_3-lh | V | visual subdivision 3 |
| V4-LH | 7Networks_LH_Vis_4-lh | V | visual subdivision 4 |
| V5-LH | 7Networks_LH_Vis_5-lh | V | visual subdivision 5 |
| V6-LH | 7Networks_LH_Vis_6-lh | V | visual subdivision 6 |
| V7-LH | 7Networks_LH_Vis_7-lh | V | visual subdivision 7 |
| V8-LH | 7Networks_LH_Vis_8-lh | V | visual subdivision 8 |
| V9-LH | 7Networks_LH_Vis_9-lh | V | visual subdivision 9 |
| C_Cing1-RH | 7Networks_RH_Cont_Cing_1-rh | C | cingulate subdivision 1 |
| C_PFCl1-RH | 7Networks_RH_Cont_PFCl_1-rh | C | lateral prefrontal cortex subdivision 1 |
| C_PFCl2-RH | 7Networks_RH_Cont_PFCl_2-rh | C | lateral prefrontal cortex subdivision 2 |
| C_PFCl3-RH | 7Networks_RH_Cont_PFCl_3-rh | C | lateral prefrontal cortex subdivision 3 |
| C_PFCl4-RH | 7Networks_RH_Cont_PFCl_4-rh | C | lateral prefrontal cortex subdivision 4 |
| C_PFCmp1-RH | 7Networks_RH_Cont_PFCmp_1-rh | C | medial posterior prefrontal cortex subdivision 1 |
| C_Par1-RH | 7Networks_RH_Cont_Par_1-rh | C | parietal subdivision 1 |
| C_Par2-RH | 7Networks_RH_Cont_Par_2-rh | C | parietal subdivision 2 |
| C_pCun1-RH | 7Networks_RH_Cont_pCun_1-rh | C | precuneus subdivision 1 |
| D_PFCdm1-RH | 7Networks_RH_Default_PFCdPFCm_1-rh | D | dorsomedial prefrontal cortex subdivision 1 |
| D_PFCdm2-RH | 7Networks_RH_Default_PFCdPFCm_2-rh | D | dorsomedial prefrontal cortex subdivision 2 |
| D_PFCdm3-RH | 7Networks_RH_Default_PFCdPFCm_3-rh | D | dorsomedial prefrontal cortex subdivision 3 |
| D_PFCv1-RH | 7Networks_RH_Default_PFCv_1-rh | D | ventral prefrontal cortex subdivision 1 |
| D_PFCv2-RH | 7Networks_RH_Default_PFCv_2-rh | D | ventral prefrontal cortex subdivision 2 |
| D_Par1-RH | 7Networks_RH_Default_Par_1-rh | D | parietal subdivision 1 |
| D_Temp1-RH | 7Networks_RH_Default_Temp_1-rh | D | temporal subdivision 1 |
| D_Temp2-RH | 7Networks_RH_Default_Temp_2-rh | D | temporal subdivision 2 |
| D_Temp3-RH | 7Networks_RH_Default_Temp_3-rh | D | temporal subdivision 3 |
| D_pCunPCC1-RH | 7Networks_RH_Default_pCunPCC_1-rh | D | precuneus posterior cingulate cortex subdivision 1 |
| D_pCunPCC2-RH | 7Networks_RH_Default_pCunPCC_2-rh | D | precuneus posterior cingulate cortex subdivision 2 |
| DA_FEF1-RH | 7Networks_RH_DorsAttn_FEF_1-rh | DA | frontal eye fields subdivision 1 |
| DA_Post1-RH | 7Networks_RH_DorsAttn_Post_1-rh | DA | posterior subdivision 1 |
| DA_Post2-RH | 7Networks_RH_DorsAttn_Post_2-rh | DA | posterior subdivision 2 |
| DA_Post3-RH | 7Networks_RH_DorsAttn_Post_3-rh | DA | posterior subdivision 3 |
| DA_Post4-RH | 7Networks_RH_DorsAttn_Post_4-rh | DA | posterior subdivision 4 |
| DA_Post5-RH | 7Networks_RH_DorsAttn_Post_5-rh | DA | posterior subdivision 5 |
| DA_PrCv1-RH | 7Networks_RH_DorsAttn_PrCv_1-rh | DA | precentral ventral subdivision 1 |
| L_OFC1-RH | 7Networks_RH_Limbic_OFC_1-rh | L | orbital frontal cortex subdivision 1 |
| L_TempPole1-RH | 7Networks_RH_Limbic_TempPole_1-rh | L | temporal pole subdivision 1 |
| SVA_FOI1-RH | 7Networks_RH_SalVentAttn_FrOperIns_1-rh | SVA | frontal operculum and insula subdivision 1 |
| SVA_Med1-RH | 7Networks_RH_SalVentAttn_Med_1-rh | SVA | medial prefrontal cortex subdivision 1 |
| SVA_Med2-RH | 7Networks_RH_SalVentAttn_Med_2-rh | SVA | medial prefrontal cortex subdivision 2 |
| SVA_TOP1-RH | 7Networks_RH_SalVentAttn_TempOccPar_1-rh | SVA | temporal parietal subdivision 1 |
| SVA_TOP2-RH | 7Networks_RH_SalVentAttn_TempOccPar_2-rh | SVA | temporal parietal subdivision 2 |
| SM1-RH | 7Networks_RH_SomMot_1-rh | SM | somatomotor subdivision 1 |
| SM2-RH | 7Networks_RH_SomMot_2-rh | SM | somatomotor subdivision 2 |
| SM3-RH | 7Networks_RH_SomMot_3-rh | SM | somatomotor subdivision 3 |
| SM4-RH | 7Networks_RH_SomMot_4-rh | SM | somatomotor subdivision 4 |
| SM5-RH | 7Networks_RH_SomMot_5-rh | SM | somatomotor subdivision 5 |
| SM6-RH | 7Networks_RH_SomMot_6-rh | SM | somatomotor subdivision 6 |
| SM7-RH | 7Networks_RH_SomMot_7-rh | SM | somatomotor subdivision 7 |
| SM8-RH | 7Networks_RH_SomMot_8-rh | SM | somatomotor subdivision 8 |
| V1-RH | 7Networks_RH_Vis_1-rh | V | visual subdivision 1 |
| V2-RH | 7Networks_RH_Vis_2-rh | V | visual subdivision 2 |
| V3-RH | 7Networks_RH_Vis_3-rh | V | visual subdivision 3 |
| V4-RH | 7Networks_RH_Vis_4-rh | V | visual subdivision 4 |
| V5-RH | 7Networks_RH_Vis_5-rh | V | visual subdivision 5 |
| V6-RH | 7Networks_RH_Vis_6-rh | V | visual subdivision 6 |
| V7-RH | 7Networks_RH_Vis_7-rh | V | visual subdivision 7 |
| V8-RH | 7Networks_RH_Vis_8-rh | V | visual subdivision 8 |

## *Global network qEEG biomarker aggregation across hemispheres*

To determine whether a significant difference existed between the left and right hemisphere signals and account for effects of handedness, we tested for the homogeneity of variances using Levene’s test (Levene, Howard, 1960). When the variances were found to be significantly different (Levene’s p<0.5), the Brunner-Munzel test was employed for the paired comparison (Brunner and Munzel, 2000). In cases where variances were comparable, the Wilcoxon signed-rank test was applied. p values were corrected using the Benjamini-Hochberg False Discovery Rate (FDR) method. For each region, the decision to reject the null hypothesis (i.e., no significant hemispheric difference) was made based on the corrected *p* value (*p_corr_*<0.05). Based on the statistical outcome, the method for combining hemispheric values was determined as follows:

1. *Significant Hemispheric Differences*: If the FDR-corrected test indicated a significant difference between LH and RH, we employed one of two approaches:
   1. *Unequal variances* (Levene’s p < 0.05): weighted combination was calculated using weights proportional to the inverse of the sample variances for LH and RH.

$$w_{LH}= \frac{1/{\sigma^{2}}_{LH}}{\frac{1}{{\sigma^{2}}_{LH}}+\frac{1}{{\sigma^{2}}_{RH}}}$$

$$w_{RH}= \frac{1/{\sigma^{2}}_{RH}}{\frac{1}{{\sigma^{2}}_{LH}}+\frac{1}{{\sigma^{2}}_{RH}}}$$

$$X_{combined}= w_{LH}.X_{LH}+ w_{RH}. X_{RH}$$

- 1. *Equal variances*: the hemisphere with the smaller variance was chosen as the representative value.

$$X_{combined}=\left\{ \begin{aligned} X_{LH}, if &{\sigma^{2}}_{LH}<&{\sigma^{2}}_{RH} \\ X_{RH}, otherwise \end{aligned} \right.$$

1. *No Significant Hemispheric Differences*: values from LH and RH were averaged.

$$X_{combined}= \frac{X_{LH}+ X_{RH}}{2}$$

## *Statistical comparison of clinical scales between STXBP1 and SYT1 patient sub-groups*

Clinical scales (Vineland AGI, GMFCS, MACS, CFCS, EEG rank) were compared between individuals with *STXBP1* and *SYT1* using Mann–Whitney U tests. Given the small sample sizes (*STXBP1*: *n* = 10; *SYT1*: *n* = 5), these analyses were considered exploratory, and results were interpreted descriptively. We report the U statistics, p-values, rank biserial correlation r (effect size) and confidence intervals for r estimated using 10000 bootstraps.

## *Principal component selection for TDC normative biomarker space*

To determine the optimal number of principal components (PCs) to retain across biomarker categories, we employed a systematic approach combining scree plot analysis with variance-based decision criteria. For each biomarker, we examined the eigenvalue decay patterns and calculated the incremental (unit) gain in cumulative variance explained by each successive component. Our selection criteria were based on identifying the elbow point in scree plots where additional components provided diminishing returns in variance explanation. Specifically, we retained components where the unit gain in variance remained substantial (typically >5% per component) and capped extraction at 5 PCs when subsequent components contributed marginal additional variance (<3-5% individually). This threshold was consistently applied across all biomarker types to ensure methodological uniformity and to balance dimensionality reduction with information preservation. As illustrated in Supplementary Figure S2 , the first 5 PCs consistently captured the majority of meaningful variance across biomarker categories.

References

Becker, D., Creutzfeldt, O. D., Schwibbe, M., and Wuttke, W. (1982). Changes in physiological, eeg and psychological parameters in women during the spontaneous menstrual cycle and following oral contraceptives. *Psychoneuroendocrinology* 7, 75–90. doi: 10.1016/0306-4530(82)90057-9

Brötzner, C. P., Klimesch, W., Doppelmayr, M., Zauner, A., and Kerschbaum, H. H. (2014). Resting state alpha frequency is associated with menstrual cycle phase, estradiol and use of oral contraceptives. *Brain Res* 1577, 36–44. doi: 10.1016/j.brainres.2014.06.034

Brunner, E., and Munzel, U. (2000). The Nonparametric Behrens-Fisher Problem: Asymptotic Theory and a Small-Sample Approximation. *Biometrical Journal* 42, 17–25. doi: 10.1002/(SICI)1521-4036(200001)42:1%3C17::AID-BIMJ17%3E3.0.CO;2-U

Fisher, R. S., Cross, J. H., French, J. A., Higurashi, N., Hirsch, E., Jansen, F. E., et al. (2017). Operational classification of seizure types by the International League Against Epilepsy: Position Paper of the ILAE Commission for Classification and Terminology. *Epilepsia* 58, 522–530. doi: 10.1111/epi.13670

Klusmann, H., Eisenlohr-Moul, T., Baresich, K., Schmalenberger, K. M., Girdler, S., and Andersen, E. (2023). Analyzing the atypical – Methods for studying the menstrual cycle in adolescents. *Psychoneuroendocrinology* 158, 106389. doi: 10.1016/j.psyneuen.2023.106389

Kothe, C. A., and Makeig, S. (2013). BCILAB: a platform for brain-computer interface development. *J Neural Eng* 10, 056014. doi: 10.1088/1741-2560/10/5/056014

Lacroix, A. E., Gondal, H., Shumway, K. R., and Langaker, M. D. (2025). “Physiology, Menarche,” in *StatPearls*, (Treasure Island (FL): StatPearls Publishing). Available at: http://www.ncbi.nlm.nih.gov/books/NBK470216/ (Accessed December 3, 2025).

Levene, Howard (1960). “Robust Tests for Equality of Variances,” in *Contributions to Probability and Statistics: Essays in Honor of Harold Hotelling Editor: Olkin, Ingram; Ghurye, S. G.; Hoeffding, Wassily; Madow, William G.; Mann, H. B.*, (Stanford, CA: Stanford University Press), 278–292.

Mansfield, M. J., and Emans, S. J. (1984). Adolescent menstrual irregularity. *J Reprod Med* 29, 399–410.

Packheiser, J., Schmitz, J., Pan, Y., El Basbasse, Y., Friedrich, P., Güntürkün, O., et al. (2020). Using Mobile EEG to Investigate Alpha and Beta Asymmetries During Hand and Foot Use. *Front. Neurosci.* 14. doi: 10.3389/fnins.2020.00109

Pion-Tonachini, L., Kreutz-Delgado, K., and Makeig, S. (2019). ICLabel: An automated electroencephalographic independent component classifier, dataset, and website. *Neuroimage* 198, 181–197. doi: 10.1016/j.neuroimage.2019.05.026

Propper, R., Pierce, J., Geisler, M., Christman, S., and Bellorado, N. (2012). Asymmetry in Resting Alpha Activity: Effects of Handedness. *Open Journal of Medical Psychology*. doi: 10.4236/ojmp.2012.14014

Schaefer, A., Kong, R., Gordon, E. M., Laumann, T. O., Zuo, X.-N., Holmes, A. J., et al. (2018). Local-Global Parcellation of the Human Cerebral Cortex from Intrinsic Functional Connectivity MRI. *Cerebral Cortex* 28, 3095–3114. doi: 10.1093/cercor/bhx179

Tomasi, D., and Volkow, N. D. (2024). Associations between handedness and brain functional connectivity patterns in children. *Nat Commun* 15, 2355. doi: 10.1038/s41467-024-46690-1
