# Supplementary material for: Dimensionality reduction of quantitative EEG and clinical profiles uncover associations with monogenic neurodevelopmental phenotypes in SNAREopathies
Source: Front Neurosci. 2026 Jan 27;19:1725623. doi: 10.3389/fnins.2025.1725623 (PMC12886349; doi:10.3389/fnins.2025.1725623)
Supplement: Supplementary file 2 [file Data_Sheet_2.docx]

# **Supplementary results**

## ***Figure S1: Selection of principal components for TDC biomarker normative space***

**a)** absolute power, **c)** relative power, and **e)** long-range temporal correlation (LRTC) exponent scree plots showing explained variance ratio as a function of number of principal components. Red dotted line shows the elbow at the 5^th^ principal component. Solid red line indicates the explained variance ratio across components for the selected alpha sparsity parameter (1.5), grey lines for other tested alpha values. **b)** absolute power, **d)** relative power, **f)** LRTC exponent marginal variance gain as a function of number of principal components. Green bars indicate PCs selected; red bars indicate PCs not included in analysis. Note, the low gain in unit variance after 5^th^ PC.

## ***S2: Small sample sizes restrict comparison of clinical scales between STXBP1 and SYT1 sub-groups***

Clinical scales were compared between individuals with *STXBP1* (*n* = 10) and *SYT1* (*n* = 5) using Mann–Whitney U tests. On the Vineland Adaptive Behavior Composite (Fig. S1 a, f), *STXBP1* showed lower scores than *SYT1* (median = 60, IQR = 50), with a positive rank-biserial effect size, indicating that higher Vineland scores tend to occur more often in *SYT1* than *STXBP1*. For GMFCS, MACS, and CFCS (where higher levels indicate greater impairment), median levels were higher in *STXBP1* than *SYT1* (Fig. S1). Correspondingly, rank-biserial correlations were negative for all three. EEG severity ranks were similar between groups (with an effect size close to zero, providing no evidence of a directional difference in EEG rank distributions. All confidence intervals showed unreliability of the estimated effect size with interval bounds crossing zero for all the tests reflecting the small sample sizes. Taken together, these exploratory analyses suggest possible directional differences in adaptive behaviour and motor/communication scales.

## ***Figure S2: Comparison of clinical scales between STXBP1 and SYT1 patient sub-groups***

**a)-e)** Comparison of the five clinical scales between *STXBP1* and *SYT1* sub-groups. U = Mann-Whitney test statistic, p = p value(significant set at 0.05), bi-serial r = rank biserial correlation coefficient as the effect size, r C.I. = lower and upper bounds of Confidence Interval for r (10000 bootstraps). **f)-g)** Proportion of patients for *STXBP1* and *SYT1* subgroups, across different levels of severity for each clinical scale. Light to dark blue gradient indicates levels of severity.

## ***Figure S3: qEEG biomarker loadings across all principal components and spatio-spectral dimension***

**a)** absolute power, **b)** relative power, and **c)** long-range temporal correlation (LRTC) exponent loadings. Each feature represents the loading strength of absolute power for a combination of frequency band (δ: delta, 1–4 Hz; θ: theta, 4–8.2 Hz; α: alpha, 8.2–13.3 Hz; β: lower beta, 13.3 –21.7 Hz ; β-γ: beta-gamma, 21.7 –44.8 Hz ) and Yeo network (DEF = Default Mode, DA = Dorsal Attention, VIS = Visual, LIM = Limbic, CON = Control, SM = Somatomotor, SVA = Salience Ventral Attention). Blue coloured bars indicate negative while red indicate positive loading directions.

## ***Figure S3: TDC EOR normative absolute power captures changes in physiology during eyes closed rest state***

**a),d)** Absolute power projection space for the reference cohort (typically developing children (TDC) in eyes-open rest (EOR), *n* = 96. Gray dots represent the TDC under EOR (*n* = 96), magenta dots represent the identical TDC under eyes-closed rest (ECR) condition, *black star* represents the centre of the TDC EOR absolute power. Mahalanobis distances were computed for each TDC, under both EOR and ECR conditions as the distance between the reference EOR centre and individual position in the projection space. **b),e)** TDC Mahalanobis distances (MD) under EOR and ECR conditions. c,f) Differences between MD ECR and MD EOR for TDC. *p*-values are reported after FDR adjustment

See Figure S1 legend for feature details.

## ***Figure S4: TDC EOR normative relative power captures changes in physiology during eyes closed rest state***

See Figure S1 legend for feature details.

## ***Supplementary Table 1,2***

| **Biomarker** | **MD type** | **PC subspace** | **Clinical PCA** | **ρ (spearman)** | **p** | **Lower C.I.** | **Upper C.I.** | **p (bootstrap)** |
| --- | --- | --- | --- | --- | --- | --- | --- | --- |
| Absolute Power | Global MD | PC1_PC2 | PC1_clin_ | 0.30 | 0.27 | -0.27 | 0.75 | 0.29 |
|  |  | PC1_PC3 |  | 0.42 | 0.12 | -0.12 | 0.77 | 0.12 |
|  |  | PC1_PC4 |  | 0.19 | 0.50 | -0.36 | 0.77 | 0.56 |
|  |  | PC1_PC5 |  | 0.36 | 0.19 | -0.17 | 0.78 | 0.18 |
|  |  | PC2_PC3 |  | 0.37 | 0.17 | -0.19 | 0.80 | 0.19 |
|  |  | PC2_PC4 |  | 0.19 | 0.49 | -0.39 | 0.67 | 0.49 |
|  |  | PC2_PC5 |  | 0.45 | 0.09 | -0.12 | 0.85 | 0.12 |
|  |  | PC3_PC4 |  | 0.20 | 0.47 | -0.36 | 0.67 | 0.46 |
|  |  | PC3_PC5 |  | 0.58 | 0.02 | 0.05 | 0.87 | 0.03 |
|  |  | PC4_PC5 |  | 0.16 | 0.56 | -0.38 | 0.69 | 0.57 |
|  |  | PC1_PC2 | PC2_clin_ | -0.29 | 0.30 | -0.80 | 0.43 | 0.40 |
|  |  | PC1_PC3 |  | -0.25 | 0.36 | -0.70 | 0.30 | 0.37 |
|  |  | PC1_PC4 |  | -0.22 | 0.43 | -0.69 | 0.35 | 0.45 |
|  |  | PC1_PC5 |  | -0.27 | 0.32 | -0.78 | 0.40 | 0.41 |
|  |  | PC2_PC3 |  | -0.21 | 0.45 | -0.67 | 0.35 | 0.45 |
|  |  | PC2_PC4 |  | -0.35 | 0.21 | -0.72 | 0.18 | 0.19 |
|  |  | PC2_PC5 |  | -0.25 | 0.36 | -0.73 | 0.35 | 0.42 |
|  |  | PC3_PC4 |  | -0.24 | 0.38 | -0.63 | 0.27 | 0.35 |
|  |  | PC3_PC5 |  | -0.29 | 0.30 | -0.71 | 0.25 | 0.28 |
|  |  | PC4_PC5 |  | -0.31 | 0.26 | -0.67 | 0.20 | 0.23 |
|  |  | PC1_PC2 | PC12_clin_ | -0.03 | 0.92 | -0.65 | 0.52 | 0.92 |
|  |  | PC1_PC3 |  | -0.18 | 0.52 | -0.69 | 0.40 | 0.54 |
|  |  | PC1_PC4 |  | -0.17 | 0.54 | -0.73 | 0.45 | 0.60 |
|  |  | PC1_PC5 |  | -0.21 | 0.44 | -0.79 | 0.42 | 0.51 |
|  |  | PC2_PC3 |  | -0.37 | 0.17 | -0.80 | 0.23 | 0.20 |
|  |  | PC2_PC4 |  | -0.37 | 0.17 | -0.82 | 0.27 | 0.23 |
|  |  | PC2_PC5 |  | -0.37 | 0.17 | -0.81 | 0.23 | 0.21 |
|  |  | PC3_PC4 |  | -0.31 | 0.27 | -0.77 | 0.40 | 0.35 |
|  |  | PC3_PC5 |  | -0.31 | 0.27 | -0.74 | 0.31 | 0.29 |
|  |  | PC4_PC5 |  | -0.30 | 0.28 | -0.79 | 0.42 | 0.38 |
|  | Age matched MD | PC1_PC2 | PC1_clin_ | 0.69 | 0.00 | 0.31 | 0.88 | 0.00 |
|  |  | PC1_PC3 |  | 0.62 | 0.01 | 0.06 | 0.93 | 0.03 |
|  |  | PC1_PC4 |  | 0.48 | 0.07 | -0.05 | 0.84 | 0.08 |
|  |  | PC1_PC5 |  | 0.63 | 0.01 | 0.16 | 0.91 | 0.01 |
|  |  | PC2_PC3 |  | 0.19 | 0.49 | -0.36 | 0.68 | 0.48 |
|  |  | PC2_PC4 |  | 0.26 | 0.35 | -0.30 | 0.73 | 0.36 |
|  |  | PC2_PC5 |  | 0.29 | 0.29 | -0.24 | 0.71 | 0.28 |
|  |  | PC3_PC4 |  | 0.41 | 0.12 | -0.18 | 0.78 | 0.15 |
|  |  | PC3_PC5 |  | 0.50 | 0.06 | -0.07 | 0.84 | 0.08 |
|  |  | PC4_PC5 |  | 0.23 | 0.41 | -0.33 | 0.71 | 0.43 |
|  |  | PC1_PC2 | PC2_clin_ | -0.13 | 0.64 | -0.73 | 0.52 | 0.70 |
|  |  | PC1_PC3 |  | -0.14 | 0.61 | -0.64 | 0.44 | 0.64 |
|  |  | PC1_PC4 |  | -0.18 | 0.53 | -0.65 | 0.40 | 0.55 |
|  |  | PC1_PC5 |  | -0.27 | 0.33 | -0.77 | 0.34 | 0.38 |
|  |  | PC2_PC3 |  | -0.28 | 0.31 | -0.80 | 0.35 | 0.38 |
|  |  | PC2_PC4 |  | -0.34 | 0.22 | -0.69 | 0.19 | 0.19 |
|  |  | PC2_PC5 |  | -0.15 | 0.58 | -0.76 | 0.46 | 0.65 |
|  |  | PC3_PC4 |  | -0.28 | 0.31 | -0.71 | 0.24 | 0.27 |
|  |  | PC3_PC5 |  | -0.42 | 0.12 | -0.83 | 0.15 | 0.14 |
|  |  | PC4_PC5 |  | -0.41 | 0.12 | -0.72 | 0.08 | 0.09 |
|  |  | PC1_PC2 | PC12_clin_ | 0.06 | 0.83 | -0.58 | 0.67 | 0.89 |
|  |  | PC1_PC3 |  | -0.10 | 0.71 | -0.63 | 0.45 | 0.70 |
|  |  | PC1_PC4 |  | -0.16 | 0.56 | -0.69 | 0.47 | 0.59 |
|  |  | PC1_PC5 |  | -0.16 | 0.58 | -0.70 | 0.45 | 0.60 |
|  |  | PC2_PC3 |  | -0.47 | 0.08 | -0.86 | 0.08 | 0.09 |
|  |  | PC2_PC4 |  | -0.39 | 0.15 | -0.83 | 0.26 | 0.21 |
|  |  | PC2_PC5 |  | -0.21 | 0.45 | -0.70 | 0.45 | 0.49 |
|  |  | PC3_PC4 |  | -0.46 | 0.08 | -0.80 | 0.08 | 0.09 |
|  |  | PC3_PC5 |  | -0.38 | 0.16 | -0.82 | 0.18 | 0.17 |
|  |  | PC4_PC5 |  | -0.35 | 0.21 | -0.79 | 0.33 | 0.28 |
| Relative Power | Global MD | PC1_PC2 | PC1_clin_ | 0.16 | 0.56 | -0.39 | 0.73 | 0.58 |
|  |  | PC1_PC3 |  | 0.46 | 0.09 | -0.06 | 0.76 | 0.08 |
|  |  | PC1_PC4 |  | -0.11 | 0.70 | -0.59 | 0.46 | 0.70 |
|  |  | PC1_PC5 |  | 0.12 | 0.67 | -0.51 | 0.62 | 0.71 |
|  |  | PC2_PC3 |  | 0.70 | 0.00 | 0.32 | 0.86 | 0.00 |
|  |  | PC2_PC4 |  | 0.10 | 0.71 | -0.49 | 0.64 | 0.74 |
|  |  | PC2_PC5 |  | 0.35 | 0.21 | -0.22 | 0.75 | 0.21 |
|  |  | PC3_PC4 |  | 0.54 | 0.04 | 0.04 | 0.85 | 0.04 |
|  |  | PC3_PC5 |  | 0.67 | 0.01 | 0.24 | 0.88 | 0.01 |
|  |  | PC4_PC5 |  | 0.14 | 0.63 | -0.40 | 0.62 | 0.62 |
|  |  | PC1_PC2 | PC2_clin_ | 0.25 | 0.36 | -0.32 | 0.71 | 0.39 |
|  |  | PC1_PC3 |  | -0.15 | 0.59 | -0.63 | 0.44 | 0.61 |
|  |  | PC1_PC4 |  | 0.39 | 0.16 | -0.16 | 0.78 | 0.16 |
|  |  | PC1_PC5 |  | -0.16 | 0.56 | -0.67 | 0.43 | 0.57 |
|  |  | PC2_PC3 |  | 0.03 | 0.91 | -0.56 | 0.56 | 0.88 |
|  |  | PC2_PC4 |  | 0.04 | 0.89 | -0.50 | 0.57 | 0.89 |
|  |  | PC2_PC5 |  | 0.04 | 0.88 | -0.58 | 0.62 | 0.88 |
|  |  | PC3_PC4 |  | 0.06 | 0.83 | -0.52 | 0.58 | 0.82 |
|  |  | PC3_PC5 |  | 0.08 | 0.79 | -0.48 | 0.56 | 0.76 |
|  |  | PC4_PC5 |  | 0.13 | 0.66 | -0.47 | 0.64 | 0.68 |
|  |  | PC1_PC2 | PC12_clin_ | 0.49 | 0.06 | -0.07 | 0.83 | 0.09 |
|  |  | PC1_PC3 |  | -0.08 | 0.77 | -0.62 | 0.50 | 0.79 |
|  |  | PC1_PC4 |  | 0.45 | 0.09 | -0.11 | 0.80 | 0.10 |
|  |  | PC1_PC5 |  | 0.00 | 0.99 | -0.53 | 0.56 | 1.00 |
|  |  | PC2_PC3 |  | 0.00 | 0.99 | -0.58 | 0.56 | 0.97 |
|  |  | PC2_PC4 |  | 0.51 | 0.05 | -0.01 | 0.84 | 0.06 |
|  |  | PC2_PC5 |  | 0.33 | 0.23 | -0.25 | 0.78 | 0.26 |
|  |  | PC3_PC4 |  | -0.01 | 0.97 | -0.57 | 0.57 | 0.96 |
|  |  | PC3_PC5 |  | -0.23 | 0.41 | -0.76 | 0.40 | 0.45 |
|  |  | PC4_PC5 |  | 0.37 | 0.18 | -0.19 | 0.80 | 0.19 |
|  | Age matched MD | PC1_PC2 | PC1_clin_ | 0.13 | 0.64 | -0.43 | 0.64 | 0.65 |
|  |  | PC1_PC3 |  | 0.31 | 0.26 | -0.26 | 0.74 | 0.27 |
|  |  | PC1_PC4 |  | 0.12 | 0.67 | -0.45 | 0.63 | 0.71 |
|  |  | PC1_PC5 |  | -0.13 | 0.66 | -0.66 | 0.44 | 0.66 |
|  |  | PC2_PC3 |  | 0.51 | 0.05 | 0.01 | 0.82 | 0.04 |
|  |  | PC2_PC4 |  | 0.31 | 0.27 | -0.29 | 0.76 | 0.31 |
|  |  | PC2_PC5 |  | 0.24 | 0.40 | -0.33 | 0.70 | 0.42 |
|  |  | PC3_PC4 |  | 0.36 | 0.19 | -0.18 | 0.75 | 0.19 |
|  |  | PC3_PC5 |  | 0.34 | 0.22 | -0.22 | 0.82 | 0.23 |
|  |  | PC4_PC5 |  | 0.16 | 0.56 | -0.36 | 0.70 | 0.61 |
|  |  | PC1_PC2 | PC2_clin_ | 0.10 | 0.72 | -0.50 | 0.63 | 0.76 |
|  |  | PC1_PC3 |  | 0.24 | 0.38 | -0.33 | 0.65 | 0.37 |
|  |  | PC1_PC4 |  | 0.23 | 0.41 | -0.28 | 0.69 | 0.40 |
|  |  | PC1_PC5 |  | -0.25 | 0.38 | -0.70 | 0.38 | 0.42 |
|  |  | PC2_PC3 |  | 0.14 | 0.61 | -0.44 | 0.60 | 0.59 |
|  |  | PC2_PC4 |  | -0.14 | 0.63 | -0.64 | 0.43 | 0.64 |
|  |  | PC2_PC5 |  | 0.25 | 0.37 | -0.35 | 0.75 | 0.41 |
|  |  | PC3_PC4 |  | 0.15 | 0.60 | -0.41 | 0.62 | 0.61 |
|  |  | PC3_PC5 |  | 0.12 | 0.68 | -0.50 | 0.54 | 0.66 |
|  |  | PC4_PC5 |  | 0.03 | 0.92 | -0.53 | 0.55 | 0.91 |
|  |  | PC1_PC2 | PC12_clin_ | 0.42 | 0.12 | -0.18 | 0.85 | 0.16 |
|  |  | PC1_PC3 |  | 0.00 | 0.99 | -0.54 | 0.57 | 1.00 |
|  |  | PC1_PC4 |  | 0.27 | 0.33 | -0.34 | 0.74 | 0.37 |
|  |  | PC1_PC5 |  | -0.30 | 0.28 | -0.78 | 0.33 | 0.34 |
|  |  | PC2_PC3 |  | -0.05 | 0.85 | -0.61 | 0.57 | 0.83 |
|  |  | PC2_PC4 |  | 0.35 | 0.20 | -0.20 | 0.75 | 0.20 |
|  |  | PC2_PC5 |  | 0.14 | 0.62 | -0.48 | 0.70 | 0.68 |
|  |  | PC3_PC4 |  | 0.16 | 0.56 | -0.39 | 0.67 | 0.57 |
|  |  | PC3_PC5 |  | -0.19 | 0.49 | -0.69 | 0.42 | 0.51 |
|  |  | PC4_PC5 |  | 0.30 | 0.28 | -0.25 | 0.72 | 0.29 |
| DFA | Global MD | PC1_PC2 | PC1_clin_ | 0.08 | 0.78 | -0.55 | 0.57 | 0.77 |
|  |  | PC1_PC3 |  | 0.20 | 0.47 | -0.45 | 0.65 | 0.50 |
|  |  | PC1_PC4 |  | 0.27 | 0.32 | -0.28 | 0.70 | 0.32 |
|  |  | PC1_PC5 |  | 0.17 | 0.54 | -0.43 | 0.69 | 0.58 |
|  |  | PC2_PC3 |  | 0.04 | 0.90 | -0.61 | 0.62 | 0.89 |
|  |  | PC2_PC4 |  | 0.47 | 0.08 | -0.15 | 0.87 | 0.12 |
|  |  | PC2_PC5 |  | 0.37 | 0.17 | -0.22 | 0.88 | 0.22 |
|  |  | PC3_PC4 |  | 0.52 | 0.04 | -0.05 | 0.86 | 0.07 |
|  |  | PC3_PC5 |  | 0.59 | 0.02 | 0.08 | 0.88 | 0.03 |
|  |  | PC4_PC5 |  | 0.56 | 0.03 | -0.02 | 0.89 | 0.06 |
|  |  | PC1_PC2 | PC2_clin_ | -0.73 | 0.00 | -0.92 | -0.34 | 0.00 |
|  |  | PC1_PC3 |  | -0.75 | 0.00 | -0.90 | -0.41 | 0.00 |
|  |  | PC1_PC4 |  | -0.56 | 0.03 | -0.86 | -0.03 | 0.04 |
|  |  | PC1_PC5 |  | -0.49 | 0.06 | -0.80 | -0.01 | 0.05 |
|  |  | PC2_PC3 |  | -0.14 | 0.61 | -0.66 | 0.44 | 0.64 |
|  |  | PC2_PC4 |  | -0.30 | 0.28 | -0.77 | 0.30 | 0.33 |
|  |  | PC2_PC5 |  | 0.17 | 0.55 | -0.38 | 0.65 | 0.57 |
|  |  | PC3_PC4 |  | -0.17 | 0.54 | -0.69 | 0.42 | 0.57 |
|  |  | PC3_PC5 |  | -0.07 | 0.80 | -0.65 | 0.52 | 0.81 |
|  |  | PC4_PC5 |  | -0.14 | 0.62 | -0.69 | 0.50 | 0.67 |
|  |  | PC1_PC2 | PC12_clin_ | 0.18 | 0.53 | -0.41 | 0.74 | 0.56 |
|  |  | PC1_PC3 |  | 0.19 | 0.49 | -0.41 | 0.70 | 0.50 |
|  |  | PC1_PC4 |  | 0.17 | 0.55 | -0.39 | 0.67 | 0.56 |
|  |  | PC1_PC5 |  | 0.27 | 0.33 | -0.32 | 0.76 | 0.36 |
|  |  | PC2_PC3 |  | -0.07 | 0.80 | -0.60 | 0.55 | 0.79 |
|  |  | PC2_PC4 |  | -0.10 | 0.72 | -0.64 | 0.51 | 0.71 |
|  |  | PC2_PC5 |  | 0.10 | 0.71 | -0.43 | 0.62 | 0.72 |
|  |  | PC3_PC4 |  | 0.09 | 0.74 | -0.50 | 0.61 | 0.78 |
|  |  | PC3_PC5 |  | 0.00 | 0.99 | -0.58 | 0.57 | 0.99 |
|  |  | PC4_PC5 |  | 0.13 | 0.64 | -0.48 | 0.65 | 0.70 |
|  | Age matched MD | PC1_PC2 | PC1_clin_ | 0.19 | 0.50 | -0.40 | 0.67 | 0.53 |
|  |  | PC1_PC3 |  | 0.20 | 0.48 | -0.47 | 0.71 | 0.55 |
|  |  | PC1_PC4 |  | 0.27 | 0.33 | -0.35 | 0.75 | 0.40 |
|  |  | PC1_PC5 |  | 0.54 | 0.04 | -0.02 | 0.90 | 0.06 |
|  |  | PC2_PC3 |  | -0.13 | 0.66 | -0.63 | 0.39 | 0.65 |
|  |  | PC2_PC4 |  | 0.50 | 0.06 | -0.11 | 0.92 | 0.10 |
|  |  | PC2_PC5 |  | 0.14 | 0.61 | -0.49 | 0.71 | 0.68 |
|  |  | PC3_PC4 |  | 0.53 | 0.04 | -0.05 | 0.90 | 0.07 |
|  |  | PC3_PC5 |  | 0.59 | 0.02 | 0.07 | 0.87 | 0.03 |
|  |  | PC4_PC5 |  | 0.58 | 0.02 | 0.04 | 0.88 | 0.04 |
|  |  | PC1_PC2 | PC2_clin_ | -0.87 | 0.00 | -0.97 | -0.60 | 0.00 |
|  |  | PC1_PC3 |  | -0.72 | 0.00 | -0.88 | -0.36 | 0.00 |
|  |  | PC1_PC4 |  | -0.50 | 0.06 | -0.76 | -0.06 | 0.03 |
|  |  | PC1_PC5 |  | -0.48 | 0.07 | -0.79 | 0.00 | 0.05 |
|  |  | PC2_PC3 |  | -0.02 | 0.94 | -0.55 | 0.52 | 0.94 |
|  |  | PC2_PC4 |  | -0.26 | 0.34 | -0.70 | 0.30 | 0.34 |
|  |  | PC2_PC5 |  | 0.17 | 0.55 | -0.40 | 0.70 | 0.59 |
|  |  | PC3_PC4 |  | -0.19 | 0.49 | -0.64 | 0.32 | 0.45 |
|  |  | PC3_PC5 |  | 0.03 | 0.92 | -0.49 | 0.58 | 0.95 |
|  |  | PC4_PC5 |  | -0.04 | 0.89 | -0.56 | 0.52 | 0.92 |
|  |  | PC1_PC2 | PC12_clin_ | 0.05 | 0.87 | -0.54 | 0.61 | 0.88 |
|  |  | PC1_PC3 |  | 0.07 | 0.80 | -0.61 | 0.65 | 0.84 |
|  |  | PC1_PC4 |  | 0.14 | 0.62 | -0.42 | 0.65 | 0.64 |
|  |  | PC1_PC5 |  | 0.25 | 0.37 | -0.37 | 0.71 | 0.40 |
|  |  | PC2_PC3 |  | 0.25 | 0.38 | -0.41 | 0.71 | 0.43 |
|  |  | PC2_PC4 |  | -0.15 | 0.59 | -0.66 | 0.47 | 0.60 |
|  |  | PC2_PC5 |  | 0.18 | 0.52 | -0.40 | 0.68 | 0.55 |
|  |  | PC3_PC4 |  | -0.01 | 0.98 | -0.52 | 0.54 | 0.97 |
|  |  | PC3_PC5 |  | 0.12 | 0.67 | -0.45 | 0.69 | 0.72 |
|  |  | PC4_PC5 |  | -0.10 | 0.73 | -0.58 | 0.47 | 0.71 |

| ID | Group | Biomarker | **PC_Pair** | **MD_Type** | **MD_Distance** | **PC1_cli** | **PC2_cli** |
| --- | --- | --- | --- | --- | --- | --- | --- |
| zsaP1 | STXBP1 | AP | PC1_PC2 | age | 1.91 | 0.94 | 1.21 |
|  |  |  | PC1_PC2 | global | 1.10 | 0.94 | 1.21 |
|  |  |  | PC1_PC3 | age | 1.76 | 0.94 | 1.21 |
|  |  |  | PC1_PC3 | global | 1.31 | 0.94 | 1.21 |
|  |  |  | PC1_PC4 | age | 2.06 | 0.94 | 1.21 |
|  |  |  | PC1_PC4 | global | 2.18 | 0.94 | 1.21 |
|  |  |  | PC1_PC5 | age | 1.47 | 0.94 | 1.21 |
|  |  |  | PC1_PC5 | global | 0.45 | 0.94 | 1.21 |
|  |  |  | PC2_PC3 | age | 0.85 | 0.94 | 1.21 |
|  |  |  | PC2_PC3 | global | 0.95 | 0.94 | 1.21 |
|  |  |  | PC2_PC4 | age | 1.36 | 0.94 | 1.21 |
|  |  |  | PC2_PC4 | global | 1.82 | 0.94 | 1.21 |
|  |  |  | PC2_PC5 | age | 1.06 | 0.94 | 1.21 |
|  |  |  | PC2_PC5 | global | 0.99 | 0.94 | 1.21 |
|  |  |  | PC3_PC4 | age | 1.50 | 0.94 | 1.21 |
|  |  |  | PC3_PC4 | global | 2.02 | 0.94 | 1.21 |
|  |  |  | PC3_PC5 | age | 0.93 | 0.94 | 1.21 |
|  |  |  | PC3_PC5 | global | 1.08 | 0.94 | 1.21 |
|  |  |  | PC4_PC5 | age | 1.49 | 0.94 | 1.21 |
|  |  |  | PC4_PC5 | global | 2.12 | 0.94 | 1.21 |
|  |  | DFA | PC1_PC2 | age | 0.49 | 0.94 | 1.21 |
|  |  |  | PC1_PC2 | global | 0.79 | 0.94 | 1.21 |
|  |  |  | PC1_PC3 | age | 1.21 | 0.94 | 1.21 |
|  |  |  | PC1_PC3 | global | 1.54 | 0.94 | 1.21 |
|  |  |  | PC1_PC4 | age | 2.23 | 0.94 | 1.21 |
|  |  |  | PC1_PC4 | global | 4.11 | 0.94 | 1.21 |
|  |  |  | PC1_PC5 | age | 2.21 | 0.94 | 1.21 |
|  |  |  | PC1_PC5 | global | 2.10 | 0.94 | 1.21 |
|  |  |  | PC2_PC3 | age | 1.23 | 0.94 | 1.21 |
|  |  |  | PC2_PC3 | global | 1.32 | 0.94 | 1.21 |
|  |  |  | PC2_PC4 | age | 3.36 | 0.94 | 1.21 |
|  |  |  | PC2_PC4 | global | 4.72 | 0.94 | 1.21 |
|  |  |  | PC2_PC5 | age | 1.88 | 0.94 | 1.21 |
|  |  |  | PC2_PC5 | global | 1.71 | 0.94 | 1.21 |
|  |  |  | PC3_PC4 | age | 3.48 | 0.94 | 1.21 |
|  |  |  | PC3_PC4 | global | 4.91 | 0.94 | 1.21 |
|  |  |  | PC3_PC5 | age | 1.76 | 0.94 | 1.21 |
|  |  |  | PC3_PC5 | global | 1.71 | 0.94 | 1.21 |
|  |  |  | PC4_PC5 | age | 3.33 | 0.94 | 1.21 |
|  |  |  | PC4_PC5 | global | 5.56 | 0.94 | 1.21 |
|  |  | RP | PC1_PC2 | age | 3.74 | 0.94 | 1.21 |
|  |  |  | PC1_PC2 | global | 3.45 | 0.94 | 1.21 |
|  |  |  | PC1_PC3 | age | 2.28 | 0.94 | 1.21 |
|  |  |  | PC1_PC3 | global | 1.24 | 0.94 | 1.21 |
|  |  |  | PC1_PC4 | age | 7.55 | 0.94 | 1.21 |
|  |  |  | PC1_PC4 | global | 8.42 | 0.94 | 1.21 |
|  |  |  | PC1_PC5 | age | 0.85 | 0.94 | 1.21 |
|  |  |  | PC1_PC5 | global | 0.37 | 0.94 | 1.21 |
|  |  |  | PC2_PC3 | age | 3.83 | 0.94 | 1.21 |
|  |  |  | PC2_PC3 | global | 3.22 | 0.94 | 1.21 |
|  |  |  | PC2_PC4 | age | 7.51 | 0.94 | 1.21 |
|  |  |  | PC2_PC4 | global | 8.87 | 0.94 | 1.21 |
|  |  |  | PC2_PC5 | age | 4.61 | 0.94 | 1.21 |
|  |  |  | PC2_PC5 | global | 4.56 | 0.94 | 1.21 |
|  |  |  | PC3_PC4 | age | 7.51 | 0.94 | 1.21 |
|  |  |  | PC3_PC4 | global | 5.97 | 0.94 | 1.21 |
|  |  |  | PC3_PC5 | age | 1.61 | 0.94 | 1.21 |
|  |  |  | PC3_PC5 | global | 1.27 | 0.94 | 1.21 |
|  |  |  | PC4_PC5 | age | 7.95 | 0.94 | 1.21 |
|  |  |  | PC4_PC5 | global | 8.11 | 0.94 | 1.21 |
| P10 | *STXBP1* | AP | PC1_PC2 | age | 0.72 | -2.38 | -0.97 |
|  |  |  | PC1_PC2 | global | 1.44 | -2.38 | -0.97 |
|  |  |  | PC1_PC3 | age | 0.54 | -2.38 | -0.97 |
|  |  |  | PC1_PC3 | global | 1.79 | -2.38 | -0.97 |
|  |  |  | PC1_PC4 | age | 1.53 | -2.38 | -0.97 |
|  |  |  | PC1_PC4 | global | 1.97 | -2.38 | -0.97 |
|  |  |  | PC1_PC5 | age | 0.88 | -2.38 | -0.97 |
|  |  |  | PC1_PC5 | global | 1.20 | -2.38 | -0.97 |
|  |  |  | PC2_PC3 | age | 0.72 | -2.38 | -0.97 |
|  |  |  | PC2_PC3 | global | 0.84 | -2.38 | -0.97 |
|  |  |  | PC2_PC4 | age | 1.94 | -2.38 | -0.97 |
|  |  |  | PC2_PC4 | global | 2.85 | -2.38 | -0.97 |
|  |  |  | PC2_PC5 | age | 0.87 | -2.38 | -0.97 |
|  |  |  | PC2_PC5 | global | 0.80 | -2.38 | -0.97 |
|  |  |  | PC3_PC4 | age | 1.64 | -2.38 | -0.97 |
|  |  |  | PC3_PC4 | global | 3.07 | -2.38 | -0.97 |
|  |  |  | PC3_PC5 | age | 0.83 | -2.38 | -0.97 |
|  |  |  | PC3_PC5 | global | 0.86 | -2.38 | -0.97 |
|  |  |  | PC4_PC5 | age | 1.98 | -2.38 | -0.97 |
|  |  |  | PC4_PC5 | global | 2.58 | -2.38 | -0.97 |
|  |  | DFA | PC1_PC2 | age | 2.69 | -2.38 | -0.97 |
|  |  |  | PC1_PC2 | global | 3.22 | -2.38 | -0.97 |
|  |  |  | PC1_PC3 | age | 3.53 | -2.38 | -0.97 |
|  |  |  | PC1_PC3 | global | 4.09 | -2.38 | -0.97 |
|  |  |  | PC1_PC4 | age | 2.68 | -2.38 | -0.97 |
|  |  |  | PC1_PC4 | global | 3.65 | -2.38 | -0.97 |
|  |  |  | PC1_PC5 | age | 3.60 | -2.38 | -0.97 |
|  |  |  | PC1_PC5 | global | 4.23 | -2.38 | -0.97 |
|  |  |  | PC2_PC3 | age | 1.05 | -2.38 | -0.97 |
|  |  |  | PC2_PC3 | global | 0.87 | -2.38 | -0.97 |
|  |  |  | PC2_PC4 | age | 0.90 | -2.38 | -0.97 |
|  |  |  | PC2_PC4 | global | 1.68 | -2.38 | -0.97 |
|  |  |  | PC2_PC5 | age | 1.20 | -2.38 | -0.97 |
|  |  |  | PC2_PC5 | global | 1.08 | -2.38 | -0.97 |
|  |  |  | PC3_PC4 | age | 1.52 | -2.38 | -0.97 |
|  |  |  | PC3_PC4 | global | 1.97 | -2.38 | -0.97 |
|  |  |  | PC3_PC5 | age | 1.07 | -2.38 | -0.97 |
|  |  |  | PC3_PC5 | global | 1.01 | -2.38 | -0.97 |
|  |  |  | PC4_PC5 | age | 1.52 | -2.38 | -0.97 |
|  |  |  | PC4_PC5 | global | 2.47 | -2.38 | -0.97 |
|  |  | RP | PC1_PC2 | age | 1.06 | -2.38 | -0.97 |
|  |  |  | PC1_PC2 | global | 1.25 | -2.38 | -0.97 |
|  |  |  | PC1_PC3 | age | 1.44 | -2.38 | -0.97 |
|  |  |  | PC1_PC3 | global | 1.37 | -2.38 | -0.97 |
|  |  |  | PC1_PC4 | age | 1.23 | -2.38 | -0.97 |
|  |  |  | PC1_PC4 | global | 1.56 | -2.38 | -0.97 |
|  |  |  | PC1_PC5 | age | 0.92 | -2.38 | -0.97 |
|  |  |  | PC1_PC5 | global | 1.11 | -2.38 | -0.97 |
|  |  |  | PC2_PC3 | age | 0.82 | -2.38 | -0.97 |
|  |  |  | PC2_PC3 | global | 1.05 | -2.38 | -0.97 |
|  |  |  | PC2_PC4 | age | 1.14 | -2.38 | -0.97 |
|  |  |  | PC2_PC4 | global | 1.60 | -2.38 | -0.97 |
|  |  |  | PC2_PC5 | age | 0.67 | -2.38 | -0.97 |
|  |  |  | PC2_PC5 | global | 1.03 | -2.38 | -0.97 |
|  |  |  | PC3_PC4 | age | 1.29 | -2.38 | -0.97 |
|  |  |  | PC3_PC4 | global | 1.56 | -2.38 | -0.97 |
|  |  |  | PC3_PC5 | age | 0.60 | -2.38 | -0.97 |
|  |  |  | PC3_PC5 | global | 0.75 | -2.38 | -0.97 |
|  |  |  | PC4_PC5 | age | 1.15 | -2.38 | -0.97 |
|  |  |  | PC4_PC5 | global | 1.58 | -2.38 | -0.97 |
| P11 | *SYT1* | AP | PC1_PC2 | age | 1.84 | -2.66 | 0.47 |
|  |  |  | PC1_PC2 | global | 2.34 | -2.66 | 0.47 |
|  |  |  | PC1_PC3 | age | 0.29 | -2.66 | 0.47 |
|  |  |  | PC1_PC3 | global | 1.40 | -2.66 | 0.47 |
|  |  |  | PC1_PC4 | age | 1.60 | -2.66 | 0.47 |
|  |  |  | PC1_PC4 | global | 1.76 | -2.66 | 0.47 |
|  |  |  | PC1_PC5 | age | 0.50 | -2.66 | 0.47 |
|  |  |  | PC1_PC5 | global | 1.41 | -2.66 | 0.47 |
|  |  |  | PC2_PC3 | age | 2.16 | -2.66 | 0.47 |
|  |  |  | PC2_PC3 | global | 2.30 | -2.66 | 0.47 |
|  |  |  | PC2_PC4 | age | 1.96 | -2.66 | 0.47 |
|  |  |  | PC2_PC4 | global | 2.32 | -2.66 | 0.47 |
|  |  |  | PC2_PC5 | age | 1.97 | -2.66 | 0.47 |
|  |  |  | PC2_PC5 | global | 2.58 | -2.66 | 0.47 |
|  |  |  | PC3_PC4 | age | 1.75 | -2.66 | 0.47 |
|  |  |  | PC3_PC4 | global | 1.73 | -2.66 | 0.47 |
|  |  |  | PC3_PC5 | age | 0.66 | -2.66 | 0.47 |
|  |  |  | PC3_PC5 | global | 0.80 | -2.66 | 0.47 |
|  |  |  | PC4_PC5 | age | 1.74 | -2.66 | 0.47 |
|  |  |  | PC4_PC5 | global | 1.83 | -2.66 | 0.47 |
|  |  | DFA | PC1_PC2 | age | 1.03 | -2.66 | 0.47 |
|  |  |  | PC1_PC2 | global | 2.13 | -2.66 | 0.47 |
|  |  |  | PC1_PC3 | age | 1.32 | -2.66 | 0.47 |
|  |  |  | PC1_PC3 | global | 1.25 | -2.66 | 0.47 |
|  |  |  | PC1_PC4 | age | 0.97 | -2.66 | 0.47 |
|  |  |  | PC1_PC4 | global | 1.36 | -2.66 | 0.47 |
|  |  |  | PC1_PC5 | age | 1.98 | -2.66 | 0.47 |
|  |  |  | PC1_PC5 | global | 1.38 | -2.66 | 0.47 |
|  |  |  | PC2_PC3 | age | 1.29 | -2.66 | 0.47 |
|  |  |  | PC2_PC3 | global | 3.32 | -2.66 | 0.47 |
|  |  |  | PC2_PC4 | age | 0.65 | -2.66 | 0.47 |
|  |  |  | PC2_PC4 | global | 2.03 | -2.66 | 0.47 |
|  |  |  | PC2_PC5 | age | 2.13 | -2.66 | 0.47 |
|  |  |  | PC2_PC5 | global | 2.44 | -2.66 | 0.47 |
|  |  |  | PC3_PC4 | age | 0.87 | -2.66 | 0.47 |
|  |  |  | PC3_PC4 | global | 0.67 | -2.66 | 0.47 |
|  |  |  | PC3_PC5 | age | 2.14 | -2.66 | 0.47 |
|  |  |  | PC3_PC5 | global | 1.06 | -2.66 | 0.47 |
|  |  |  | PC4_PC5 | age | 1.98 | -2.66 | 0.47 |
|  |  |  | PC4_PC5 | global | 1.27 | -2.66 | 0.47 |
|  |  | RP | PC1_PC2 | age | 2.00 | -2.66 | 0.47 |
|  |  |  | PC1_PC2 | global | 3.05 | -2.66 | 0.47 |
|  |  |  | PC1_PC3 | age | 2.18 | -2.66 | 0.47 |
|  |  |  | PC1_PC3 | global | 2.55 | -2.66 | 0.47 |
|  |  |  | PC1_PC4 | age | 1.54 | -2.66 | 0.47 |
|  |  |  | PC1_PC4 | global | 2.81 | -2.66 | 0.47 |
|  |  |  | PC1_PC5 | age | 1.49 | -2.66 | 0.47 |
|  |  |  | PC1_PC5 | global | 2.31 | -2.66 | 0.47 |
|  |  |  | PC2_PC3 | age | 1.80 | -2.66 | 0.47 |
|  |  |  | PC2_PC3 | global | 0.51 | -2.66 | 0.47 |
|  |  |  | PC2_PC4 | age | 1.51 | -2.66 | 0.47 |
|  |  |  | PC2_PC4 | global | 2.80 | -2.66 | 0.47 |
|  |  |  | PC2_PC5 | age | 1.20 | -2.66 | 0.47 |
|  |  |  | PC2_PC5 | global | 0.87 | -2.66 | 0.47 |
|  |  |  | PC3_PC4 | age | 1.80 | -2.66 | 0.47 |
|  |  |  | PC3_PC4 | global | 1.52 | -2.66 | 0.47 |
|  |  |  | PC3_PC5 | age | 1.66 | -2.66 | 0.47 |
|  |  |  | PC3_PC5 | global | 0.54 | -2.66 | 0.47 |
|  |  |  | PC4_PC5 | age | 1.01 | -2.66 | 0.47 |
|  |  |  | PC4_PC5 | global | 2.16 | -2.66 | 0.47 |
| P12 | *SYT1* | AP | PC1_PC2 | age | 1.06 | -1.95 | -0.74 |
|  |  |  | PC1_PC2 | global | 1.62 | -1.95 | -0.74 |
|  |  |  | PC1_PC3 | age | 0.42 | -1.95 | -0.74 |
|  |  |  | PC1_PC3 | global | 1.10 | -1.95 | -0.74 |
|  |  |  | PC1_PC4 | age | 1.31 | -1.95 | -0.74 |
|  |  |  | PC1_PC4 | global | 1.24 | -1.95 | -0.74 |
|  |  |  | PC1_PC5 | age | 1.20 | -1.95 | -0.74 |
|  |  |  | PC1_PC5 | global | 1.26 | -1.95 | -0.74 |
|  |  |  | PC2_PC3 | age | 1.06 | -1.95 | -0.74 |
|  |  |  | PC2_PC3 | global | 1.13 | -1.95 | -0.74 |
|  |  |  | PC2_PC4 | age | 1.50 | -1.95 | -0.74 |
|  |  |  | PC2_PC4 | global | 1.25 | -1.95 | -0.74 |
|  |  |  | PC2_PC5 | age | 1.30 | -1.95 | -0.74 |
|  |  |  | PC2_PC5 | global | 1.22 | -1.95 | -0.74 |
|  |  |  | PC3_PC4 | age | 1.35 | -1.95 | -0.74 |
|  |  |  | PC3_PC4 | global | 0.98 | -1.95 | -0.74 |
|  |  |  | PC3_PC5 | age | 1.21 | -1.95 | -0.74 |
|  |  |  | PC3_PC5 | global | 0.72 | -1.95 | -0.74 |
|  |  |  | PC4_PC5 | age | 1.57 | -1.95 | -0.74 |
|  |  |  | PC4_PC5 | global | 1.10 | -1.95 | -0.74 |
|  |  | DFA | PC1_PC2 | age | 2.37 | -1.95 | -0.74 |
|  |  |  | PC1_PC2 | global | 3.11 | -1.95 | -0.74 |
|  |  |  | PC1_PC3 | age | 2.42 | -1.95 | -0.74 |
|  |  |  | PC1_PC3 | global | 4.07 | -1.95 | -0.74 |
|  |  |  | PC1_PC4 | age | 2.18 | -1.95 | -0.74 |
|  |  |  | PC1_PC4 | global | 3.86 | -1.95 | -0.74 |
|  |  |  | PC1_PC5 | age | 2.33 | -1.95 | -0.74 |
|  |  |  | PC1_PC5 | global | 3.43 | -1.95 | -0.74 |
|  |  |  | PC2_PC3 | age | 0.60 | -1.95 | -0.74 |
|  |  |  | PC2_PC3 | global | 1.43 | -1.95 | -0.74 |
|  |  |  | PC2_PC4 | age | 1.86 | -1.95 | -0.74 |
|  |  |  | PC2_PC4 | global | 3.21 | -1.95 | -0.74 |
|  |  |  | PC2_PC5 | age | 0.63 | -1.95 | -0.74 |
|  |  |  | PC2_PC5 | global | 0.78 | -1.95 | -0.74 |
|  |  |  | PC3_PC4 | age | 1.45 | -1.95 | -0.74 |
|  |  |  | PC3_PC4 | global | 2.89 | -1.95 | -0.74 |
|  |  |  | PC3_PC5 | age | 0.62 | -1.95 | -0.74 |
|  |  |  | PC3_PC5 | global | 1.50 | -1.95 | -0.74 |
|  |  |  | PC4_PC5 | age | 1.26 | -1.95 | -0.74 |
|  |  |  | PC4_PC5 | global | 3.13 | -1.95 | -0.74 |
|  |  | RP | PC1_PC2 | age | 1.00 | -1.95 | -0.74 |
|  |  |  | PC1_PC2 | global | 1.15 | -1.95 | -0.74 |
|  |  |  | PC1_PC3 | age | 1.00 | -1.95 | -0.74 |
|  |  |  | PC1_PC3 | global | 1.12 | -1.95 | -0.74 |
|  |  |  | PC1_PC4 | age | 1.08 | -1.95 | -0.74 |
|  |  |  | PC1_PC4 | global | 1.36 | -1.95 | -0.74 |
|  |  |  | PC1_PC5 | age | 1.00 | -1.95 | -0.74 |
|  |  |  | PC1_PC5 | global | 1.10 | -1.95 | -0.74 |
|  |  |  | PC2_PC3 | age | 0.55 | -1.95 | -0.74 |
|  |  |  | PC2_PC3 | global | 0.67 | -1.95 | -0.74 |
|  |  |  | PC2_PC4 | age | 1.09 | -1.95 | -0.74 |
|  |  |  | PC2_PC4 | global | 1.37 | -1.95 | -0.74 |
|  |  |  | PC2_PC5 | age | 0.17 | -1.95 | -0.74 |
|  |  |  | PC2_PC5 | global | 0.55 | -1.95 | -0.74 |
|  |  |  | PC3_PC4 | age | 0.91 | -1.95 | -0.74 |
|  |  |  | PC3_PC4 | global | 1.30 | -1.95 | -0.74 |
|  |  |  | PC3_PC5 | age | 0.52 | -1.95 | -0.74 |
|  |  |  | PC3_PC5 | global | 0.29 | -1.95 | -0.74 |
|  |  |  | PC4_PC5 | age | 0.93 | -1.95 | -0.74 |
|  |  |  | PC4_PC5 | global | 1.30 | -1.95 | -0.74 |
| P13 | *SYT1* | AP | PC1_PC2 | age | 1.27 | -2.77 | 0.45 |
|  |  |  | PC1_PC2 | global | 1.57 | -2.77 | 0.45 |
|  |  |  | PC1_PC3 | age | 1.26 | -2.77 | 0.45 |
|  |  |  | PC1_PC3 | global | 1.31 | -2.77 | 0.45 |
|  |  |  | PC1_PC4 | age | 1.62 | -2.77 | 0.45 |
|  |  |  | PC1_PC4 | global | 2.06 | -2.77 | 0.45 |
|  |  |  | PC1_PC5 | age | 1.37 | -2.77 | 0.45 |
|  |  |  | PC1_PC5 | global | 1.33 | -2.77 | 0.45 |
|  |  |  | PC2_PC3 | age | 1.16 | -2.77 | 0.45 |
|  |  |  | PC2_PC3 | global | 0.63 | -2.77 | 0.45 |
|  |  |  | PC2_PC4 | age | 0.92 | -2.77 | 0.45 |
|  |  |  | PC2_PC4 | global | 0.85 | -2.77 | 0.45 |
|  |  |  | PC2_PC5 | age | 1.45 | -2.77 | 0.45 |
|  |  |  | PC2_PC5 | global | 0.90 | -2.77 | 0.45 |
|  |  |  | PC3_PC4 | age | 1.35 | -2.77 | 0.45 |
|  |  |  | PC3_PC4 | global | 1.37 | -2.77 | 0.45 |
|  |  |  | PC3_PC5 | age | 1.06 | -2.77 | 0.45 |
|  |  |  | PC3_PC5 | global | 0.65 | -2.77 | 0.45 |
|  |  |  | PC4_PC5 | age | 1.69 | -2.77 | 0.45 |
|  |  |  | PC4_PC5 | global | 1.32 | -2.77 | 0.45 |
|  |  | DFA | PC1_PC2 | age | 2.03 | -2.77 | 0.45 |
|  |  |  | PC1_PC2 | global | 2.27 | -2.77 | 0.45 |
|  |  |  | PC1_PC3 | age | 1.28 | -2.77 | 0.45 |
|  |  |  | PC1_PC3 | global | 2.62 | -2.77 | 0.45 |
|  |  |  | PC1_PC4 | age | 2.54 | -2.77 | 0.45 |
|  |  |  | PC1_PC4 | global | 3.25 | -2.77 | 0.45 |
|  |  |  | PC1_PC5 | age | 1.32 | -2.77 | 0.45 |
|  |  |  | PC1_PC5 | global | 2.69 | -2.77 | 0.45 |
|  |  |  | PC2_PC3 | age | 1.52 | -2.77 | 0.45 |
|  |  |  | PC2_PC3 | global | 0.86 | -2.77 | 0.45 |
|  |  |  | PC2_PC4 | age | 1.76 | -2.77 | 0.45 |
|  |  |  | PC2_PC4 | global | 0.93 | -2.77 | 0.45 |
|  |  |  | PC2_PC5 | age | 1.52 | -2.77 | 0.45 |
|  |  |  | PC2_PC5 | global | 0.90 | -2.77 | 0.45 |
|  |  |  | PC3_PC4 | age | 1.34 | -2.77 | 0.45 |
|  |  |  | PC3_PC4 | global | 0.80 | -2.77 | 0.45 |
|  |  |  | PC3_PC5 | age | 0.34 | -2.77 | 0.45 |
|  |  |  | PC3_PC5 | global | 0.60 | -2.77 | 0.45 |
|  |  |  | PC4_PC5 | age | 1.54 | -2.77 | 0.45 |
|  |  |  | PC4_PC5 | global | 0.84 | -2.77 | 0.45 |
|  |  | RP | PC1_PC2 | age | 1.05 | -2.77 | 0.45 |
|  |  |  | PC1_PC2 | global | 0.91 | -2.77 | 0.45 |
|  |  |  | PC1_PC3 | age | 2.01 | -2.77 | 0.45 |
|  |  |  | PC1_PC3 | global | 1.21 | -2.77 | 0.45 |
|  |  |  | PC1_PC4 | age | 1.12 | -2.77 | 0.45 |
|  |  |  | PC1_PC4 | global | 0.93 | -2.77 | 0.45 |
|  |  |  | PC1_PC5 | age | 2.53 | -2.77 | 0.45 |
|  |  |  | PC1_PC5 | global | 1.31 | -2.77 | 0.45 |
|  |  |  | PC2_PC3 | age | 1.20 | -2.77 | 0.45 |
|  |  |  | PC2_PC3 | global | 1.18 | -2.77 | 0.45 |
|  |  |  | PC2_PC4 | age | 0.18 | -2.77 | 0.45 |
|  |  |  | PC2_PC4 | global | 0.67 | -2.77 | 0.45 |
|  |  |  | PC2_PC5 | age | 2.44 | -2.77 | 0.45 |
|  |  |  | PC2_PC5 | global | 1.52 | -2.77 | 0.45 |
|  |  |  | PC3_PC4 | age | 1.19 | -2.77 | 0.45 |
|  |  |  | PC3_PC4 | global | 1.12 | -2.77 | 0.45 |
|  |  |  | PC3_PC5 | age | 3.32 | -2.77 | 0.45 |
|  |  |  | PC3_PC5 | global | 1.49 | -2.77 | 0.45 |
|  |  |  | PC4_PC5 | age | 2.67 | -2.77 | 0.45 |
|  |  |  | PC4_PC5 | global | 1.09 | -2.77 | 0.45 |
| P14 | *SYT1* | AP | PC1_PC2 | age | 3.82 | 1.65 | -1.12 |
|  |  |  | PC1_PC2 | global | 3.48 | 1.65 | -1.12 |
|  |  |  | PC1_PC3 | age | 3.86 | 1.65 | -1.12 |
|  |  |  | PC1_PC3 | global | 3.37 | 1.65 | -1.12 |
|  |  |  | PC1_PC4 | age | 4.02 | 1.65 | -1.12 |
|  |  |  | PC1_PC4 | global | 4.03 | 1.65 | -1.12 |
|  |  |  | PC1_PC5 | age | 3.92 | 1.65 | -1.12 |
|  |  |  | PC1_PC5 | global | 3.49 | 1.65 | -1.12 |
|  |  |  | PC2_PC3 | age | 2.95 | 1.65 | -1.12 |
|  |  |  | PC2_PC3 | global | 2.45 | 1.65 | -1.12 |
|  |  |  | PC2_PC4 | age | 3.42 | 1.65 | -1.12 |
|  |  |  | PC2_PC4 | global | 3.62 | 1.65 | -1.12 |
|  |  |  | PC2_PC5 | age | 2.57 | 1.65 | -1.12 |
|  |  |  | PC2_PC5 | global | 2.97 | 1.65 | -1.12 |
|  |  |  | PC3_PC4 | age | 3.55 | 1.65 | -1.12 |
|  |  |  | PC3_PC4 | global | 3.90 | 1.65 | -1.12 |
|  |  |  | PC3_PC5 | age | 2.98 | 1.65 | -1.12 |
|  |  |  | PC3_PC5 | global | 2.82 | 1.65 | -1.12 |
|  |  |  | PC4_PC5 | age | 3.53 | 1.65 | -1.12 |
|  |  |  | PC4_PC5 | global | 3.95 | 1.65 | -1.12 |
|  |  | DFA | PC1_PC2 | age | 3.87 | 1.65 | -1.12 |
|  |  |  | PC1_PC2 | global | 3.87 | 1.65 | -1.12 |
|  |  |  | PC1_PC3 | age | 2.94 | 1.65 | -1.12 |
|  |  |  | PC1_PC3 | global | 5.11 | 1.65 | -1.12 |
|  |  |  | PC1_PC4 | age | 4.29 | 1.65 | -1.12 |
|  |  |  | PC1_PC4 | global | 6.16 | 1.65 | -1.12 |
|  |  |  | PC1_PC5 | age | 2.43 | 1.65 | -1.12 |
|  |  |  | PC1_PC5 | global | 2.61 | 1.65 | -1.12 |
|  |  |  | PC2_PC3 | age | 3.99 | 1.65 | -1.12 |
|  |  |  | PC2_PC3 | global | 4.04 | 1.65 | -1.12 |
|  |  |  | PC2_PC4 | age | 4.29 | 1.65 | -1.12 |
|  |  |  | PC2_PC4 | global | 5.90 | 1.65 | -1.12 |
|  |  |  | PC2_PC5 | age | 3.82 | 1.65 | -1.12 |
|  |  |  | PC2_PC5 | global | 3.63 | 1.65 | -1.12 |
|  |  |  | PC3_PC4 | age | 4.22 | 1.65 | -1.12 |
|  |  |  | PC3_PC4 | global | 5.95 | 1.65 | -1.12 |
|  |  |  | PC3_PC5 | age | 2.79 | 1.65 | -1.12 |
|  |  |  | PC3_PC5 | global | 4.33 | 1.65 | -1.12 |
|  |  |  | PC4_PC5 | age | 4.28 | 1.65 | -1.12 |
|  |  |  | PC4_PC5 | global | 6.26 | 1.65 | -1.12 |
|  |  | RP | PC1_PC2 | age | 2.39 | 1.65 | -1.12 |
|  |  |  | PC1_PC2 | global | 2.11 | 1.65 | -1.12 |
|  |  |  | PC1_PC3 | age | 1.42 | 1.65 | -1.12 |
|  |  |  | PC1_PC3 | global | 1.36 | 1.65 | -1.12 |
|  |  |  | PC1_PC4 | age | 2.54 | 1.65 | -1.12 |
|  |  |  | PC1_PC4 | global | 1.88 | 1.65 | -1.12 |
|  |  |  | PC1_PC5 | age | 1.79 | 1.65 | -1.12 |
|  |  |  | PC1_PC5 | global | 1.57 | 1.65 | -1.12 |
|  |  |  | PC2_PC3 | age | 2.55 | 1.65 | -1.12 |
|  |  |  | PC2_PC3 | global | 2.09 | 1.65 | -1.12 |
|  |  |  | PC2_PC4 | age | 2.59 | 1.65 | -1.12 |
|  |  |  | PC2_PC4 | global | 2.16 | 1.65 | -1.12 |
|  |  |  | PC2_PC5 | age | 2.28 | 1.65 | -1.12 |
|  |  |  | PC2_PC5 | global | 2.25 | 1.65 | -1.12 |
|  |  |  | PC3_PC4 | age | 2.39 | 1.65 | -1.12 |
|  |  |  | PC3_PC4 | global | 1.77 | 1.65 | -1.12 |
|  |  |  | PC3_PC5 | age | 1.39 | 1.65 | -1.12 |
|  |  |  | PC3_PC5 | global | 1.15 | 1.65 | -1.12 |
|  |  |  | PC4_PC5 | age | 2.40 | 1.65 | -1.12 |
|  |  |  | PC4_PC5 | global | 1.89 | 1.65 | -1.12 |
| P15 | SYT1 | AP | PC1_PC2 | age | 1.69 | 2.43 | 0.16 |
|  |  |  | PC1_PC2 | global | 2.47 | 2.43 | 0.16 |
|  |  |  | PC1_PC3 | age | 2.80 | 2.43 | 0.16 |
|  |  |  | PC1_PC3 | global | 2.52 | 2.43 | 0.16 |
|  |  |  | PC1_PC4 | age | 1.37 | 2.43 | 0.16 |
|  |  |  | PC1_PC4 | global | 1.00 | 2.43 | 0.16 |
|  |  |  | PC1_PC5 | age | 1.60 | 2.43 | 0.16 |
|  |  |  | PC1_PC5 | global | 1.25 | 2.43 | 0.16 |
|  |  |  | PC2_PC3 | age | 1.79 | 2.43 | 0.16 |
|  |  |  | PC2_PC3 | global | 2.03 | 2.43 | 0.16 |
|  |  |  | PC2_PC4 | age | 1.13 | 2.43 | 0.16 |
|  |  |  | PC2_PC4 | global | 1.99 | 2.43 | 0.16 |
|  |  |  | PC2_PC5 | age | 1.14 | 2.43 | 0.16 |
|  |  |  | PC2_PC5 | global | 2.19 | 2.43 | 0.16 |
|  |  |  | PC3_PC4 | age | 1.77 | 2.43 | 0.16 |
|  |  |  | PC3_PC4 | global | 1.67 | 2.43 | 0.16 |
|  |  |  | PC3_PC5 | age | 1.77 | 2.43 | 0.16 |
|  |  |  | PC3_PC5 | global | 1.80 | 2.43 | 0.16 |
|  |  |  | PC4_PC5 | age | 0.84 | 2.43 | 0.16 |
|  |  |  | PC4_PC5 | global | 1.01 | 2.43 | 0.16 |
|  |  | DFA | PC1_PC2 | age | 1.55 | 2.43 | 0.16 |
|  |  |  | PC1_PC2 | global | 1.74 | 2.43 | 0.16 |
|  |  |  | PC1_PC3 | age | 2.03 | 2.43 | 0.16 |
|  |  |  | PC1_PC3 | global | 2.10 | 2.43 | 0.16 |
|  |  |  | PC1_PC4 | age | 3.98 | 2.43 | 0.16 |
|  |  |  | PC1_PC4 | global | 3.39 | 2.43 | 0.16 |
|  |  |  | PC1_PC5 | age | 2.23 | 2.43 | 0.16 |
|  |  |  | PC1_PC5 | global | 1.59 | 2.43 | 0.16 |
|  |  |  | PC2_PC3 | age | 1.67 | 2.43 | 0.16 |
|  |  |  | PC2_PC3 | global | 1.72 | 2.43 | 0.16 |
|  |  |  | PC2_PC4 | age | 4.10 | 2.43 | 0.16 |
|  |  |  | PC2_PC4 | global | 3.19 | 2.43 | 0.16 |
|  |  |  | PC2_PC5 | age | 2.05 | 2.43 | 0.16 |
|  |  |  | PC2_PC5 | global | 1.82 | 2.43 | 0.16 |
|  |  |  | PC3_PC4 | age | 4.04 | 2.43 | 0.16 |
|  |  |  | PC3_PC4 | global | 3.17 | 2.43 | 0.16 |
|  |  |  | PC3_PC5 | age | 2.05 | 2.43 | 0.16 |
|  |  |  | PC3_PC5 | global | 1.65 | 2.43 | 0.16 |
|  |  |  | PC4_PC5 | age | 3.99 | 2.43 | 0.16 |
|  |  |  | PC4_PC5 | global | 3.30 | 2.43 | 0.16 |
|  |  | RP | PC1_PC2 | age | 1.36 | 2.43 | 0.16 |
|  |  |  | PC1_PC2 | global | 1.65 | 2.43 | 0.16 |
|  |  |  | PC1_PC3 | age | 2.34 | 2.43 | 0.16 |
|  |  |  | PC1_PC3 | global | 2.34 | 2.43 | 0.16 |
|  |  |  | PC1_PC4 | age | 2.20 | 2.43 | 0.16 |
|  |  |  | PC1_PC4 | global | 1.70 | 2.43 | 0.16 |
|  |  |  | PC1_PC5 | age | 1.64 | 2.43 | 0.16 |
|  |  |  | PC1_PC5 | global | 1.56 | 2.43 | 0.16 |
|  |  |  | PC2_PC3 | age | 2.07 | 2.43 | 0.16 |
|  |  |  | PC2_PC3 | global | 2.04 | 2.43 | 0.16 |
|  |  |  | PC2_PC4 | age | 2.23 | 2.43 | 0.16 |
|  |  |  | PC2_PC4 | global | 1.57 | 2.43 | 0.16 |
|  |  |  | PC2_PC5 | age | 1.38 | 2.43 | 0.16 |
|  |  |  | PC2_PC5 | global | 0.65 | 2.43 | 0.16 |
|  |  |  | PC3_PC4 | age | 3.00 | 2.43 | 0.16 |
|  |  |  | PC3_PC4 | global | 2.50 | 2.43 | 0.16 |
|  |  |  | PC3_PC5 | age | 1.75 | 2.43 | 0.16 |
|  |  |  | PC3_PC5 | global | 2.08 | 2.43 | 0.16 |
|  |  |  | PC4_PC5 | age | 2.20 | 2.43 | 0.16 |
|  |  |  | PC4_PC5 | global | 1.34 | 2.43 | 0.16 |
| P2 | *STXBP1* | AP | PC1_PC2 | age | 2.66 | 2.14 | -0.31 |
|  |  |  | PC1_PC2 | global | 2.33 | 2.14 | -0.31 |
|  |  |  | PC1_PC3 | age | 2.33 | 2.14 | -0.31 |
|  |  |  | PC1_PC3 | global | 2.48 | 2.14 | -0.31 |
|  |  |  | PC1_PC4 | age | 4.56 | 2.14 | -0.31 |
|  |  |  | PC1_PC4 | global | 3.60 | 2.14 | -0.31 |
|  |  |  | PC1_PC5 | age | 3.44 | 2.14 | -0.31 |
|  |  |  | PC1_PC5 | global | 3.25 | 2.14 | -0.31 |
|  |  |  | PC2_PC3 | age | 1.98 | 2.14 | -0.31 |
|  |  |  | PC2_PC3 | global | 2.31 | 2.14 | -0.31 |
|  |  |  | PC2_PC4 | age | 4.95 | 2.14 | -0.31 |
|  |  |  | PC2_PC4 | global | 3.68 | 2.14 | -0.31 |
|  |  |  | PC2_PC5 | age | 2.94 | 2.14 | -0.31 |
|  |  |  | PC2_PC5 | global | 3.24 | 2.14 | -0.31 |
|  |  |  | PC3_PC4 | age | 4.62 | 2.14 | -0.31 |
|  |  |  | PC3_PC4 | global | 4.02 | 2.14 | -0.31 |
|  |  |  | PC3_PC5 | age | 2.85 | 2.14 | -0.31 |
|  |  |  | PC3_PC5 | global | 3.23 | 2.14 | -0.31 |
|  |  |  | PC4_PC5 | age | 4.67 | 2.14 | -0.31 |
|  |  |  | PC4_PC5 | global | 4.19 | 2.14 | -0.31 |
|  |  | DFA | PC1_PC2 | age | 3.70 | 2.14 | -0.31 |
|  |  |  | PC1_PC2 | global | 4.17 | 2.14 | -0.31 |
|  |  |  | PC1_PC3 | age | 3.98 | 2.14 | -0.31 |
|  |  |  | PC1_PC3 | global | 5.10 | 2.14 | -0.31 |
|  |  |  | PC1_PC4 | age | 3.75 | 2.14 | -0.31 |
|  |  |  | PC1_PC4 | global | 4.94 | 2.14 | -0.31 |
|  |  |  | PC1_PC5 | age | 4.33 | 2.14 | -0.31 |
|  |  |  | PC1_PC5 | global | 4.99 | 2.14 | -0.31 |
|  |  |  | PC2_PC3 | age | 0.50 | 2.14 | -0.31 |
|  |  |  | PC2_PC3 | global | 0.91 | 2.14 | -0.31 |
|  |  |  | PC2_PC4 | age | 3.18 | 2.14 | -0.31 |
|  |  |  | PC2_PC4 | global | 4.08 | 2.14 | -0.31 |
|  |  |  | PC2_PC5 | age | 4.05 | 2.14 | -0.31 |
|  |  |  | PC2_PC5 | global | 3.68 | 2.14 | -0.31 |
|  |  |  | PC3_PC4 | age | 3.59 | 2.14 | -0.31 |
|  |  |  | PC3_PC4 | global | 3.62 | 2.14 | -0.31 |
|  |  |  | PC3_PC5 | age | 4.72 | 2.14 | -0.31 |
|  |  |  | PC3_PC5 | global | 4.27 | 2.14 | -0.31 |
|  |  |  | PC4_PC5 | age | 3.90 | 2.14 | -0.31 |
|  |  |  | PC4_PC5 | global | 4.18 | 2.14 | -0.31 |
|  |  | RP | PC1_PC2 | age | 0.82 | 2.14 | -0.31 |
|  |  |  | PC1_PC2 | global | 0.84 | 2.14 | -0.31 |
|  |  |  | PC1_PC3 | age | 1.23 | 2.14 | -0.31 |
|  |  |  | PC1_PC3 | global | 1.69 | 2.14 | -0.31 |
|  |  |  | PC1_PC4 | age | 0.02 | 2.14 | -0.31 |
|  |  |  | PC1_PC4 | global | 0.12 | 2.14 | -0.31 |
|  |  |  | PC1_PC5 | age | 2.34 | 2.14 | -0.31 |
|  |  |  | PC1_PC5 | global | 1.83 | 2.14 | -0.31 |
|  |  |  | PC2_PC3 | age | 1.95 | 2.14 | -0.31 |
|  |  |  | PC2_PC3 | global | 1.86 | 2.14 | -0.31 |
|  |  |  | PC2_PC4 | age | 0.89 | 2.14 | -0.31 |
|  |  |  | PC2_PC4 | global | 1.03 | 2.14 | -0.31 |
|  |  |  | PC2_PC5 | age | 2.57 | 2.14 | -0.31 |
|  |  |  | PC2_PC5 | global | 2.46 | 2.14 | -0.31 |
|  |  |  | PC3_PC4 | age | 1.22 | 2.14 | -0.31 |
|  |  |  | PC3_PC4 | global | 1.71 | 2.14 | -0.31 |
|  |  |  | PC3_PC5 | age | 2.83 | 2.14 | -0.31 |
|  |  |  | PC3_PC5 | global | 2.68 | 2.14 | -0.31 |
|  |  |  | PC4_PC5 | age | 2.31 | 2.14 | -0.31 |
|  |  |  | PC4_PC5 | global | 1.94 | 2.14 | -0.31 |
| P3 | *STXBP1* | AP | PC1_PC2 | age | 2.67 | 2.61 | -0.68 |
|  |  |  | PC1_PC2 | global | 1.44 | 2.61 | -0.68 |
|  |  |  | PC1_PC3 | age | 2.57 | 2.61 | -0.68 |
|  |  |  | PC1_PC3 | global | 1.63 | 2.61 | -0.68 |
|  |  |  | PC1_PC4 | age | 2.96 | 2.61 | -0.68 |
|  |  |  | PC1_PC4 | global | 1.48 | 2.61 | -0.68 |
|  |  |  | PC1_PC5 | age | 2.57 | 2.61 | -0.68 |
|  |  |  | PC1_PC5 | global | 1.75 | 2.61 | -0.68 |
|  |  |  | PC2_PC3 | age | 1.82 | 2.61 | -0.68 |
|  |  |  | PC2_PC3 | global | 1.46 | 2.61 | -0.68 |
|  |  |  | PC2_PC4 | age | 1.72 | 2.61 | -0.68 |
|  |  |  | PC2_PC4 | global | 1.38 | 2.61 | -0.68 |
|  |  |  | PC2_PC5 | age | 2.21 | 2.61 | -0.68 |
|  |  |  | PC2_PC5 | global | 2.04 | 2.61 | -0.68 |
|  |  |  | PC3_PC4 | age | 1.85 | 2.61 | -0.68 |
|  |  |  | PC3_PC4 | global | 1.67 | 2.61 | -0.68 |
|  |  |  | PC3_PC5 | age | 1.91 | 2.61 | -0.68 |
|  |  |  | PC3_PC5 | global | 1.98 | 2.61 | -0.68 |
|  |  |  | PC4_PC5 | age | 2.50 | 2.61 | -0.68 |
|  |  |  | PC4_PC5 | global | 1.73 | 2.61 | -0.68 |
|  |  | DFA | PC1_PC2 | age | 4.94 | 2.61 | -0.68 |
|  |  |  | PC1_PC2 | global | 3.47 | 2.61 | -0.68 |
|  |  |  | PC1_PC3 | age | 4.95 | 2.61 | -0.68 |
|  |  |  | PC1_PC3 | global | 4.38 | 2.61 | -0.68 |
|  |  |  | PC1_PC4 | age | 5.90 | 2.61 | -0.68 |
|  |  |  | PC1_PC4 | global | 4.61 | 2.61 | -0.68 |
|  |  |  | PC1_PC5 | age | 5.09 | 2.61 | -0.68 |
|  |  |  | PC1_PC5 | global | 3.98 | 2.61 | -0.68 |
|  |  |  | PC2_PC3 | age | 1.28 | 2.61 | -0.68 |
|  |  |  | PC2_PC3 | global | 1.07 | 2.61 | -0.68 |
|  |  |  | PC2_PC4 | age | 5.18 | 2.61 | -0.68 |
|  |  |  | PC2_PC4 | global | 4.17 | 2.61 | -0.68 |
|  |  |  | PC2_PC5 | age | 0.36 | 2.61 | -0.68 |
|  |  |  | PC2_PC5 | global | 0.86 | 2.61 | -0.68 |
|  |  |  | PC3_PC4 | age | 3.91 | 2.61 | -0.68 |
|  |  |  | PC3_PC4 | global | 3.94 | 2.61 | -0.68 |
|  |  |  | PC3_PC5 | age | 1.89 | 2.61 | -0.68 |
|  |  |  | PC3_PC5 | global | 1.38 | 2.61 | -0.68 |
|  |  |  | PC4_PC5 | age | 3.88 | 2.61 | -0.68 |
|  |  |  | PC4_PC5 | global | 4.51 | 2.61 | -0.68 |
|  |  | RP | PC1_PC2 | age | 2.04 | 2.61 | -0.68 |
|  |  |  | PC1_PC2 | global | 2.29 | 2.61 | -0.68 |
|  |  |  | PC1_PC3 | age | 3.52 | 2.61 | -0.68 |
|  |  |  | PC1_PC3 | global | 3.10 | 2.61 | -0.68 |
|  |  |  | PC1_PC4 | age | 1.90 | 2.61 | -0.68 |
|  |  |  | PC1_PC4 | global | 1.46 | 2.61 | -0.68 |
|  |  |  | PC1_PC5 | age | 1.13 | 2.61 | -0.68 |
|  |  |  | PC1_PC5 | global | 1.38 | 2.61 | -0.68 |
|  |  |  | PC2_PC3 | age | 3.48 | 2.61 | -0.68 |
|  |  |  | PC2_PC3 | global | 3.12 | 2.61 | -0.68 |
|  |  |  | PC2_PC4 | age | 2.54 | 2.61 | -0.68 |
|  |  |  | PC2_PC4 | global | 3.42 | 2.61 | -0.68 |
|  |  |  | PC2_PC5 | age | 0.99 | 2.61 | -0.68 |
|  |  |  | PC2_PC5 | global | 1.68 | 2.61 | -0.68 |
|  |  |  | PC3_PC4 | age | 4.56 | 2.61 | -0.68 |
|  |  |  | PC3_PC4 | global | 2.90 | 2.61 | -0.68 |
|  |  |  | PC3_PC5 | age | 3.48 | 2.61 | -0.68 |
|  |  |  | PC3_PC5 | global | 2.83 | 2.61 | -0.68 |
|  |  |  | PC4_PC5 | age | 2.25 | 2.61 | -0.68 |
|  |  |  | PC4_PC5 | global | 1.64 | 2.61 | -0.68 |
| P4 | *STXBP1* | AP | PC1_PC2 | age | 2.81 | 1.89 | 1.06 |
|  |  |  | PC1_PC2 | global | 2.78 | 1.89 | 1.06 |
|  |  |  | PC1_PC3 | age | 2.92 | 1.89 | 1.06 |
|  |  |  | PC1_PC3 | global | 2.98 | 1.89 | 1.06 |
|  |  |  | PC1_PC4 | age | 3.20 | 1.89 | 1.06 |
|  |  |  | PC1_PC4 | global | 3.01 | 1.89 | 1.06 |
|  |  |  | PC1_PC5 | age | 2.95 | 1.89 | 1.06 |
|  |  |  | PC1_PC5 | global | 2.81 | 1.89 | 1.06 |
|  |  |  | PC2_PC3 | age | 1.69 | 1.89 | 1.06 |
|  |  |  | PC2_PC3 | global | 2.05 | 1.89 | 1.06 |
|  |  |  | PC2_PC4 | age | 2.00 | 1.89 | 1.06 |
|  |  |  | PC2_PC4 | global | 2.27 | 1.89 | 1.06 |
|  |  |  | PC2_PC5 | age | 1.87 | 1.89 | 1.06 |
|  |  |  | PC2_PC5 | global | 2.01 | 1.89 | 1.06 |
|  |  |  | PC3_PC4 | age | 2.39 | 1.89 | 1.06 |
|  |  |  | PC3_PC4 | global | 2.70 | 1.89 | 1.06 |
|  |  |  | PC3_PC5 | age | 1.99 | 1.89 | 1.06 |
|  |  |  | PC3_PC5 | global | 2.41 | 1.89 | 1.06 |
|  |  |  | PC4_PC5 | age | 2.30 | 1.89 | 1.06 |
|  |  |  | PC4_PC5 | global | 2.57 | 1.89 | 1.06 |
|  |  | DFA | PC1_PC2 | age | 0.75 | 1.89 | 1.06 |
|  |  |  | PC1_PC2 | global | 1.63 | 1.89 | 1.06 |
|  |  |  | PC1_PC3 | age | 1.08 | 1.89 | 1.06 |
|  |  |  | PC1_PC3 | global | 2.00 | 1.89 | 1.06 |
|  |  |  | PC1_PC4 | age | 1.17 | 1.89 | 1.06 |
|  |  |  | PC1_PC4 | global | 3.12 | 1.89 | 1.06 |
|  |  |  | PC1_PC5 | age | 2.56 | 1.89 | 1.06 |
|  |  |  | PC1_PC5 | global | 2.87 | 1.89 | 1.06 |
|  |  |  | PC2_PC3 | age | 1.20 | 1.89 | 1.06 |
|  |  |  | PC2_PC3 | global | 1.22 | 1.89 | 1.06 |
|  |  |  | PC2_PC4 | age | 1.41 | 1.89 | 1.06 |
|  |  |  | PC2_PC4 | global | 3.06 | 1.89 | 1.06 |
|  |  |  | PC2_PC5 | age | 2.35 | 1.89 | 1.06 |
|  |  |  | PC2_PC5 | global | 2.20 | 1.89 | 1.06 |
|  |  |  | PC3_PC4 | age | 1.93 | 1.89 | 1.06 |
|  |  |  | PC3_PC4 | global | 3.19 | 1.89 | 1.06 |
|  |  |  | PC3_PC5 | age | 2.19 | 1.89 | 1.06 |
|  |  |  | PC3_PC5 | global | 2.29 | 1.89 | 1.06 |
|  |  |  | PC4_PC5 | age | 2.75 | 1.89 | 1.06 |
|  |  |  | PC4_PC5 | global | 4.71 | 1.89 | 1.06 |
|  |  | RP | PC1_PC2 | age | 1.62 | 1.89 | 1.06 |
|  |  |  | PC1_PC2 | global | 1.95 | 1.89 | 1.06 |
|  |  |  | PC1_PC3 | age | 3.00 | 1.89 | 1.06 |
|  |  |  | PC1_PC3 | global | 2.96 | 1.89 | 1.06 |
|  |  |  | PC1_PC4 | age | 1.57 | 1.89 | 1.06 |
|  |  |  | PC1_PC4 | global | 1.92 | 1.89 | 1.06 |
|  |  |  | PC1_PC5 | age | 0.75 | 1.89 | 1.06 |
|  |  |  | PC1_PC5 | global | 1.03 | 1.89 | 1.06 |
|  |  |  | PC2_PC3 | age | 3.03 | 1.89 | 1.06 |
|  |  |  | PC2_PC3 | global | 3.04 | 1.89 | 1.06 |
|  |  |  | PC2_PC4 | age | 1.63 | 1.89 | 1.06 |
|  |  |  | PC2_PC4 | global | 2.05 | 1.89 | 1.06 |
|  |  |  | PC2_PC5 | age | 1.67 | 1.89 | 1.06 |
|  |  |  | PC2_PC5 | global | 2.13 | 1.89 | 1.06 |
|  |  |  | PC3_PC4 | age | 3.16 | 1.89 | 1.06 |
|  |  |  | PC3_PC4 | global | 3.17 | 1.89 | 1.06 |
|  |  |  | PC3_PC5 | age | 2.79 | 1.89 | 1.06 |
|  |  |  | PC3_PC5 | global | 2.94 | 1.89 | 1.06 |
|  |  |  | PC4_PC5 | age | 1.36 | 1.89 | 1.06 |
|  |  |  | PC4_PC5 | global | 1.80 | 1.89 | 1.06 |
| P5 | *STXBP1* | AP | PC1_PC2 | age | 2.16 | 1.61 | 0.11 |
|  |  |  | PC1_PC2 | global | 2.01 | 1.61 | 0.11 |
|  |  |  | PC1_PC3 | age | 2.81 | 1.61 | 0.11 |
|  |  |  | PC1_PC3 | global | 3.84 | 1.61 | 0.11 |
|  |  |  | PC1_PC4 | age | 3.85 | 1.61 | 0.11 |
|  |  |  | PC1_PC4 | global | 3.83 | 1.61 | 0.11 |
|  |  |  | PC1_PC5 | age | 2.61 | 1.61 | 0.11 |
|  |  |  | PC1_PC5 | global | 2.46 | 1.61 | 0.11 |
|  |  |  | PC2_PC3 | age | 2.48 | 1.61 | 0.11 |
|  |  |  | PC2_PC3 | global | 3.44 | 1.61 | 0.11 |
|  |  |  | PC2_PC4 | age | 3.77 | 1.61 | 0.11 |
|  |  |  | PC2_PC4 | global | 4.00 | 1.61 | 0.11 |
|  |  |  | PC2_PC5 | age | 2.27 | 1.61 | 0.11 |
|  |  |  | PC2_PC5 | global | 2.32 | 1.61 | 0.11 |
|  |  |  | PC3_PC4 | age | 4.26 | 1.61 | 0.11 |
|  |  |  | PC3_PC4 | global | 4.59 | 1.61 | 0.11 |
|  |  |  | PC3_PC5 | age | 2.77 | 1.61 | 0.11 |
|  |  |  | PC3_PC5 | global | 3.75 | 1.61 | 0.11 |
|  |  |  | PC4_PC5 | age | 3.86 | 1.61 | 0.11 |
|  |  |  | PC4_PC5 | global | 4.21 | 1.61 | 0.11 |
|  |  | DFA | PC1_PC2 | age | 0.92 | 1.61 | 0.11 |
|  |  |  | PC1_PC2 | global | 1.27 | 1.61 | 0.11 |
|  |  |  | PC1_PC3 | age | 0.48 | 1.61 | 0.11 |
|  |  |  | PC1_PC3 | global | 1.27 | 1.61 | 0.11 |
|  |  |  | PC1_PC4 | age | 0.57 | 1.61 | 0.11 |
|  |  |  | PC1_PC4 | global | 1.69 | 1.61 | 0.11 |
|  |  |  | PC1_PC5 | age | 1.48 | 1.61 | 0.11 |
|  |  |  | PC1_PC5 | global | 1.78 | 1.61 | 0.11 |
|  |  |  | PC2_PC3 | age | 0.94 | 1.61 | 0.11 |
|  |  |  | PC2_PC3 | global | 1.40 | 1.61 | 0.11 |
|  |  |  | PC2_PC4 | age | 1.72 | 1.61 | 0.11 |
|  |  |  | PC2_PC4 | global | 2.49 | 1.61 | 0.11 |
|  |  |  | PC2_PC5 | age | 0.98 | 1.61 | 0.11 |
|  |  |  | PC2_PC5 | global | 1.15 | 1.61 | 0.11 |
|  |  |  | PC3_PC4 | age | 1.11 | 1.61 | 0.11 |
|  |  |  | PC3_PC4 | global | 1.64 | 1.61 | 0.11 |
|  |  |  | PC3_PC5 | age | 1.51 | 1.61 | 0.11 |
|  |  |  | PC3_PC5 | global | 1.54 | 1.61 | 0.11 |
|  |  |  | PC4_PC5 | age | 1.76 | 1.61 | 0.11 |
|  |  |  | PC4_PC5 | global | 2.69 | 1.61 | 0.11 |
|  |  | RP | PC1_PC2 | age | 0.72 | 1.61 | 0.11 |
|  |  |  | PC1_PC2 | global | 0.74 | 1.61 | 0.11 |
|  |  |  | PC1_PC3 | age | 5.55 | 1.61 | 0.11 |
|  |  |  | PC1_PC3 | global | 6.23 | 1.61 | 0.11 |
|  |  |  | PC1_PC4 | age | 0.30 | 1.61 | 0.11 |
|  |  |  | PC1_PC4 | global | 0.63 | 1.61 | 0.11 |
|  |  |  | PC1_PC5 | age | 0.87 | 1.61 | 0.11 |
|  |  |  | PC1_PC5 | global | 0.98 | 1.61 | 0.11 |
|  |  |  | PC2_PC3 | age | 5.25 | 1.61 | 0.11 |
|  |  |  | PC2_PC3 | global | 6.04 | 1.61 | 0.11 |
|  |  |  | PC2_PC4 | age | 1.06 | 1.61 | 0.11 |
|  |  |  | PC2_PC4 | global | 1.05 | 1.61 | 0.11 |
|  |  |  | PC2_PC5 | age | 0.76 | 1.61 | 0.11 |
|  |  |  | PC2_PC5 | global | 1.02 | 1.61 | 0.11 |
|  |  |  | PC3_PC4 | age | 5.03 | 1.61 | 0.11 |
|  |  |  | PC3_PC4 | global | 6.26 | 1.61 | 0.11 |
|  |  |  | PC3_PC5 | age | 5.35 | 1.61 | 0.11 |
|  |  |  | PC3_PC5 | global | 6.31 | 1.61 | 0.11 |
|  |  |  | PC4_PC5 | age | 0.94 | 1.61 | 0.11 |
|  |  |  | PC4_PC5 | global | 1.11 | 1.61 | 0.11 |
| P6 | *STXBP1* | AP | PC1_PC2 | age | 0.91 | -0.95 | 1.05 |
|  |  |  | PC1_PC2 | global | 0.95 | -0.95 | 1.05 |
|  |  |  | PC1_PC3 | age | 1.45 | -0.95 | 1.05 |
|  |  |  | PC1_PC3 | global | 1.72 | -0.95 | 1.05 |
|  |  |  | PC1_PC4 | age | 0.90 | -0.95 | 1.05 |
|  |  |  | PC1_PC4 | global | 1.00 | -0.95 | 1.05 |
|  |  |  | PC1_PC5 | age | 1.13 | -0.95 | 1.05 |
|  |  |  | PC1_PC5 | global | 1.29 | -0.95 | 1.05 |
|  |  |  | PC2_PC3 | age | 1.33 | -0.95 | 1.05 |
|  |  |  | PC2_PC3 | global | 1.21 | -0.95 | 1.05 |
|  |  |  | PC2_PC4 | age | 0.64 | -0.95 | 1.05 |
|  |  |  | PC2_PC4 | global | 0.68 | -0.95 | 1.05 |
|  |  |  | PC2_PC5 | age | 0.96 | -0.95 | 1.05 |
|  |  |  | PC2_PC5 | global | 1.04 | -0.95 | 1.05 |
|  |  |  | PC3_PC4 | age | 1.58 | -0.95 | 1.05 |
|  |  |  | PC3_PC4 | global | 1.32 | -0.95 | 1.05 |
|  |  |  | PC3_PC5 | age | 1.01 | -0.95 | 1.05 |
|  |  |  | PC3_PC5 | global | 0.63 | -0.95 | 1.05 |
|  |  |  | PC4_PC5 | age | 1.44 | -0.95 | 1.05 |
|  |  |  | PC4_PC5 | global | 0.91 | -0.95 | 1.05 |
|  |  | DFA | PC1_PC2 | age | 0.63 | -0.95 | 1.05 |
|  |  |  | PC1_PC2 | global | 0.58 | -0.95 | 1.05 |
|  |  |  | PC1_PC3 | age | 1.93 | -0.95 | 1.05 |
|  |  |  | PC1_PC3 | global | 2.54 | -0.95 | 1.05 |
|  |  |  | PC1_PC4 | age | 2.67 | -0.95 | 1.05 |
|  |  |  | PC1_PC4 | global | 2.68 | -0.95 | 1.05 |
|  |  |  | PC1_PC5 | age | 1.14 | -0.95 | 1.05 |
|  |  |  | PC1_PC5 | global | 1.07 | -0.95 | 1.05 |
|  |  |  | PC2_PC3 | age | 1.86 | -0.95 | 1.05 |
|  |  |  | PC2_PC3 | global | 2.31 | -0.95 | 1.05 |
|  |  |  | PC2_PC4 | age | 2.12 | -0.95 | 1.05 |
|  |  |  | PC2_PC4 | global | 2.35 | -0.95 | 1.05 |
|  |  |  | PC2_PC5 | age | 0.96 | -0.95 | 1.05 |
|  |  |  | PC2_PC5 | global | 1.01 | -0.95 | 1.05 |
|  |  |  | PC3_PC4 | age | 2.23 | -0.95 | 1.05 |
|  |  |  | PC3_PC4 | global | 2.51 | -0.95 | 1.05 |
|  |  |  | PC3_PC5 | age | 1.84 | -0.95 | 1.05 |
|  |  |  | PC3_PC5 | global | 2.33 | -0.95 | 1.05 |
|  |  |  | PC4_PC5 | age | 2.14 | -0.95 | 1.05 |
|  |  |  | PC4_PC5 | global | 2.34 | -0.95 | 1.05 |
|  |  | RP | PC1_PC2 | age | 1.33 | -0.95 | 1.05 |
|  |  |  | PC1_PC2 | global | 1.38 | -0.95 | 1.05 |
|  |  |  | PC1_PC3 | age | 1.05 | -0.95 | 1.05 |
|  |  |  | PC1_PC3 | global | 1.03 | -0.95 | 1.05 |
|  |  |  | PC1_PC4 | age | 1.46 | -0.95 | 1.05 |
|  |  |  | PC1_PC4 | global | 1.57 | -0.95 | 1.05 |
|  |  |  | PC1_PC5 | age | 1.07 | -0.95 | 1.05 |
|  |  |  | PC1_PC5 | global | 1.22 | -0.95 | 1.05 |
|  |  |  | PC2_PC3 | age | 1.50 | -0.95 | 1.05 |
|  |  |  | PC2_PC3 | global | 1.44 | -0.95 | 1.05 |
|  |  |  | PC2_PC4 | age | 1.38 | -0.95 | 1.05 |
|  |  |  | PC2_PC4 | global | 1.63 | -0.95 | 1.05 |
|  |  |  | PC2_PC5 | age | 1.06 | -0.95 | 1.05 |
|  |  |  | PC2_PC5 | global | 1.36 | -0.95 | 1.05 |
|  |  |  | PC3_PC4 | age | 1.51 | -0.95 | 1.05 |
|  |  |  | PC3_PC4 | global | 1.62 | -0.95 | 1.05 |
|  |  |  | PC3_PC5 | age | 0.90 | -0.95 | 1.05 |
|  |  |  | PC3_PC5 | global | 0.99 | -0.95 | 1.05 |
|  |  |  | PC4_PC5 | age | 1.34 | -0.95 | 1.05 |
|  |  |  | PC4_PC5 | global | 1.60 | -0.95 | 1.05 |
| P7 | *STXBP1* | AP | PC1_PC2 | age | 1.45 | -2.05 | -0.63 |
|  |  |  | PC1_PC2 | global | 1.92 | -2.05 | -0.63 |
|  |  |  | PC1_PC3 | age | 4.57 | -2.05 | -0.63 |
|  |  |  | PC1_PC3 | global | 3.77 | -2.05 | -0.63 |
|  |  |  | PC1_PC4 | age | 3.18 | -2.05 | -0.63 |
|  |  |  | PC1_PC4 | global | 3.37 | -2.05 | -0.63 |
|  |  |  | PC1_PC5 | age | 3.12 | -2.05 | -0.63 |
|  |  |  | PC1_PC5 | global | 2.37 | -2.05 | -0.63 |
|  |  |  | PC2_PC3 | age | 4.58 | -2.05 | -0.63 |
|  |  |  | PC2_PC3 | global | 3.08 | -2.05 | -0.63 |
|  |  |  | PC2_PC4 | age | 2.79 | -2.05 | -0.63 |
|  |  |  | PC2_PC4 | global | 3.28 | -2.05 | -0.63 |
|  |  |  | PC2_PC5 | age | 2.77 | -2.05 | -0.63 |
|  |  |  | PC2_PC5 | global | 2.27 | -2.05 | -0.63 |
|  |  |  | PC3_PC4 | age | 4.76 | -2.05 | -0.63 |
|  |  |  | PC3_PC4 | global | 3.96 | -2.05 | -0.63 |
|  |  |  | PC3_PC5 | age | 4.84 | -2.05 | -0.63 |
|  |  |  | PC3_PC5 | global | 3.42 | -2.05 | -0.63 |
|  |  |  | PC4_PC5 | age | 3.36 | -2.05 | -0.63 |
|  |  |  | PC4_PC5 | global | 3.54 | -2.05 | -0.63 |
|  |  | DFA | PC1_PC2 | age | 2.03 | -2.05 | -0.63 |
|  |  |  | PC1_PC2 | global | 2.55 | -2.05 | -0.63 |
|  |  |  | PC1_PC3 | age | 2.78 | -2.05 | -0.63 |
|  |  |  | PC1_PC3 | global | 3.30 | -2.05 | -0.63 |
|  |  |  | PC1_PC4 | age | 11.38 | -2.05 | -0.63 |
|  |  |  | PC1_PC4 | global | 5.29 | -2.05 | -0.63 |
|  |  |  | PC1_PC5 | age | 2.13 | -2.05 | -0.63 |
|  |  |  | PC1_PC5 | global | 2.76 | -2.05 | -0.63 |
|  |  |  | PC2_PC3 | age | 2.33 | -2.05 | -0.63 |
|  |  |  | PC2_PC3 | global | 1.59 | -2.05 | -0.63 |
|  |  |  | PC2_PC4 | age | 10.70 | -2.05 | -0.63 |
|  |  |  | PC2_PC4 | global | 6.14 | -2.05 | -0.63 |
|  |  |  | PC2_PC5 | age | 1.31 | -2.05 | -0.63 |
|  |  |  | PC2_PC5 | global | 0.66 | -2.05 | -0.63 |
|  |  |  | PC3_PC4 | age | 9.88 | -2.05 | -0.63 |
|  |  |  | PC3_PC4 | global | 5.26 | -2.05 | -0.63 |
|  |  |  | PC3_PC5 | age | 1.77 | -2.05 | -0.63 |
|  |  |  | PC3_PC5 | global | 1.21 | -2.05 | -0.63 |
|  |  |  | PC4_PC5 | age | 10.21 | -2.05 | -0.63 |
|  |  |  | PC4_PC5 | global | 5.68 | -2.05 | -0.63 |
|  |  | RP | PC1_PC2 | age | 1.91 | -2.05 | -0.63 |
|  |  |  | PC1_PC2 | global | 1.66 | -2.05 | -0.63 |
|  |  |  | PC1_PC3 | age | 2.85 | -2.05 | -0.63 |
|  |  |  | PC1_PC3 | global | 1.66 | -2.05 | -0.63 |
|  |  |  | PC1_PC4 | age | 12.73 | -2.05 | -0.63 |
|  |  |  | PC1_PC4 | global | 8.33 | -2.05 | -0.63 |
|  |  |  | PC1_PC5 | age | 1.95 | -2.05 | -0.63 |
|  |  |  | PC1_PC5 | global | 0.98 | -2.05 | -0.63 |
|  |  |  | PC2_PC3 | age | 4.34 | -2.05 | -0.63 |
|  |  |  | PC2_PC3 | global | 1.58 | -2.05 | -0.63 |
|  |  |  | PC2_PC4 | age | 14.14 | -2.05 | -0.63 |
|  |  |  | PC2_PC4 | global | 9.42 | -2.05 | -0.63 |
|  |  |  | PC2_PC5 | age | 1.27 | -2.05 | -0.63 |
|  |  |  | PC2_PC5 | global | 1.32 | -2.05 | -0.63 |
|  |  |  | PC3_PC4 | age | 22.24 | -2.05 | -0.63 |
|  |  |  | PC3_PC4 | global | 5.39 | -2.05 | -0.63 |
|  |  |  | PC3_PC5 | age | 3.45 | -2.05 | -0.63 |
|  |  |  | PC3_PC5 | global | 1.45 | -2.05 | -0.63 |
|  |  |  | PC4_PC5 | age | 13.57 | -2.05 | -0.63 |
|  |  |  | PC4_PC5 | global | 7.38 | -2.05 | -0.63 |
| P8 | *STXBP1* | AP | PC1_PC2 | age | 1.12 | -0.78 | 0.90 |
|  |  |  | PC1_PC2 | global | 1.23 | -0.78 | 0.90 |
|  |  |  | PC1_PC3 | age | 1.69 | -0.78 | 0.90 |
|  |  |  | PC1_PC3 | global | 1.36 | -0.78 | 0.90 |
|  |  |  | PC1_PC4 | age | 2.88 | -0.78 | 0.90 |
|  |  |  | PC1_PC4 | global | 2.83 | -0.78 | 0.90 |
|  |  |  | PC1_PC5 | age | 1.30 | -0.78 | 0.90 |
|  |  |  | PC1_PC5 | global | 1.40 | -0.78 | 0.90 |
|  |  |  | PC2_PC3 | age | 1.85 | -0.78 | 0.90 |
|  |  |  | PC2_PC3 | global | 0.97 | -0.78 | 0.90 |
|  |  |  | PC2_PC4 | age | 3.01 | -0.78 | 0.90 |
|  |  |  | PC2_PC4 | global | 3.07 | -0.78 | 0.90 |
|  |  |  | PC2_PC5 | age | 1.07 | -0.78 | 0.90 |
|  |  |  | PC2_PC5 | global | 1.18 | -0.78 | 0.90 |
|  |  |  | PC3_PC4 | age | 2.77 | -0.78 | 0.90 |
|  |  |  | PC3_PC4 | global | 3.22 | -0.78 | 0.90 |
|  |  |  | PC3_PC5 | age | 1.71 | -0.78 | 0.90 |
|  |  |  | PC3_PC5 | global | 1.28 | -0.78 | 0.90 |
|  |  |  | PC4_PC5 | age | 2.78 | -0.78 | 0.90 |
|  |  |  | PC4_PC5 | global | 3.09 | -0.78 | 0.90 |
|  |  | DFA | PC1_PC2 | age | 1.29 | -0.78 | 0.90 |
|  |  |  | PC1_PC2 | global | 1.30 | -0.78 | 0.90 |
|  |  |  | PC1_PC3 | age | 1.61 | -0.78 | 0.90 |
|  |  |  | PC1_PC3 | global | 1.65 | -0.78 | 0.90 |
|  |  |  | PC1_PC4 | age | 2.13 | -0.78 | 0.90 |
|  |  |  | PC1_PC4 | global | 3.17 | -0.78 | 0.90 |
|  |  |  | PC1_PC5 | age | 2.25 | -0.78 | 0.90 |
|  |  |  | PC1_PC5 | global | 1.58 | -0.78 | 0.90 |
|  |  |  | PC2_PC3 | age | 1.08 | -0.78 | 0.90 |
|  |  |  | PC2_PC3 | global | 1.03 | -0.78 | 0.90 |
|  |  |  | PC2_PC4 | age | 2.38 | -0.78 | 0.90 |
|  |  |  | PC2_PC4 | global | 3.19 | -0.78 | 0.90 |
|  |  |  | PC2_PC5 | age | 1.18 | -0.78 | 0.90 |
|  |  |  | PC2_PC5 | global | 0.92 | -0.78 | 0.90 |
|  |  |  | PC3_PC4 | age | 2.61 | -0.78 | 0.90 |
|  |  |  | PC3_PC4 | global | 3.32 | -0.78 | 0.90 |
|  |  |  | PC3_PC5 | age | 0.52 | -0.78 | 0.90 |
|  |  |  | PC3_PC5 | global | 0.46 | -0.78 | 0.90 |
|  |  |  | PC4_PC5 | age | 2.83 | -0.78 | 0.90 |
|  |  |  | PC4_PC5 | global | 3.74 | -0.78 | 0.90 |
|  |  | RP | PC1_PC2 | age | 1.94 | -0.78 | 0.90 |
|  |  |  | PC1_PC2 | global | 1.71 | -0.78 | 0.90 |
|  |  |  | PC1_PC3 | age | 1.78 | -0.78 | 0.90 |
|  |  |  | PC1_PC3 | global | 1.04 | -0.78 | 0.90 |
|  |  |  | PC1_PC4 | age | 3.06 | -0.78 | 0.90 |
|  |  |  | PC1_PC4 | global | 1.79 | -0.78 | 0.90 |
|  |  |  | PC1_PC5 | age | 1.67 | -0.78 | 0.90 |
|  |  |  | PC1_PC5 | global | 1.18 | -0.78 | 0.90 |
|  |  |  | PC2_PC3 | age | 0.90 | -0.78 | 0.90 |
|  |  |  | PC2_PC3 | global | 0.94 | -0.78 | 0.90 |
|  |  |  | PC2_PC4 | age | 0.80 | -0.78 | 0.90 |
|  |  |  | PC2_PC4 | global | 0.80 | -0.78 | 0.90 |
|  |  |  | PC2_PC5 | age | 0.83 | -0.78 | 0.90 |
|  |  |  | PC2_PC5 | global | 0.87 | -0.78 | 0.90 |
|  |  |  | PC3_PC4 | age | 0.41 | -0.78 | 0.90 |
|  |  |  | PC3_PC4 | global | 0.96 | -0.78 | 0.90 |
|  |  |  | PC3_PC5 | age | 0.79 | -0.78 | 0.90 |
|  |  |  | PC3_PC5 | global | 0.90 | -0.78 | 0.90 |
|  |  |  | PC4_PC5 | age | 0.73 | -0.78 | 0.90 |
|  |  |  | PC4_PC5 | global | 0.82 | -0.78 | 0.90 |
| P9 | *STXBP1* | AP | PC1_PC2 | age | 2.33 | 0.27 | -0.97 |
|  |  |  | PC1_PC2 | global | 2.15 | 0.27 | -0.97 |
|  |  |  | PC1_PC3 | age | 2.25 | 0.27 | -0.97 |
|  |  |  | PC1_PC3 | global | 2.32 | 0.27 | -0.97 |
|  |  |  | PC1_PC4 | age | 2.56 | 0.27 | -0.97 |
|  |  |  | PC1_PC4 | global | 2.85 | 0.27 | -0.97 |
|  |  |  | PC1_PC5 | age | 2.37 | 0.27 | -0.97 |
|  |  |  | PC1_PC5 | global | 2.25 | 0.27 | -0.97 |
|  |  |  | PC2_PC3 | age | 1.93 | 0.27 | -0.97 |
|  |  |  | PC2_PC3 | global | 1.64 | 0.27 | -0.97 |
|  |  |  | PC2_PC4 | age | 2.62 | 0.27 | -0.97 |
|  |  |  | PC2_PC4 | global | 2.67 | 0.27 | -0.97 |
|  |  |  | PC2_PC5 | age | 0.90 | 0.27 | -0.97 |
|  |  |  | PC2_PC5 | global | 1.32 | 0.27 | -0.97 |
|  |  |  | PC3_PC4 | age | 2.48 | 0.27 | -0.97 |
|  |  |  | PC3_PC4 | global | 2.93 | 0.27 | -0.97 |
|  |  |  | PC3_PC5 | age | 2.16 | 0.27 | -0.97 |
|  |  |  | PC3_PC5 | global | 1.80 | 0.27 | -0.97 |
|  |  |  | PC4_PC5 | age | 2.71 | 0.27 | -0.97 |
|  |  |  | PC4_PC5 | global | 2.97 | 0.27 | -0.97 |
|  |  | DFA | PC1_PC2 | age | 2.41 | 0.27 | -0.97 |
|  |  |  | PC1_PC2 | global | 2.10 | 0.27 | -0.97 |
|  |  |  | PC1_PC3 | age | 2.41 | 0.27 | -0.97 |
|  |  |  | PC1_PC3 | global | 3.27 | 0.27 | -0.97 |
|  |  |  | PC1_PC4 | age | 2.58 | 0.27 | -0.97 |
|  |  |  | PC1_PC4 | global | 3.45 | 0.27 | -0.97 |
|  |  |  | PC1_PC5 | age | 2.42 | 0.27 | -0.97 |
|  |  |  | PC1_PC5 | global | 2.29 | 0.27 | -0.97 |
|  |  |  | PC2_PC3 | age | 1.28 | 0.27 | -0.97 |
|  |  |  | PC2_PC3 | global | 1.92 | 0.27 | -0.97 |
|  |  |  | PC2_PC4 | age | 2.38 | 0.27 | -0.97 |
|  |  |  | PC2_PC4 | global | 3.42 | 0.27 | -0.97 |
|  |  |  | PC2_PC5 | age | 1.07 | 0.27 | -0.97 |
|  |  |  | PC2_PC5 | global | 0.93 | 0.27 | -0.97 |
|  |  |  | PC3_PC4 | age | 2.19 | 0.27 | -0.97 |
|  |  |  | PC3_PC4 | global | 3.18 | 0.27 | -0.97 |
|  |  |  | PC3_PC5 | age | 1.23 | 0.27 | -0.97 |
|  |  |  | PC3_PC5 | global | 2.04 | 0.27 | -0.97 |
|  |  |  | PC4_PC5 | age | 2.19 | 0.27 | -0.97 |
|  |  |  | PC4_PC5 | global | 3.41 | 0.27 | -0.97 |
|  |  | RP | PC1_PC2 | age | 1.63 | 0.27 | -0.97 |
|  |  |  | PC1_PC2 | global | 1.48 | 0.27 | -0.97 |
|  |  |  | PC1_PC3 | age | 1.80 | 0.27 | -0.97 |
|  |  |  | PC1_PC3 | global | 2.43 | 0.27 | -0.97 |
|  |  |  | PC1_PC4 | age | 1.29 | 0.27 | -0.97 |
|  |  |  | PC1_PC4 | global | 1.27 | 0.27 | -0.97 |
|  |  |  | PC1_PC5 | age | 1.08 | 0.27 | -0.97 |
|  |  |  | PC1_PC5 | global | 1.07 | 0.27 | -0.97 |
|  |  |  | PC2_PC3 | age | 1.69 | 0.27 | -0.97 |
|  |  |  | PC2_PC3 | global | 2.18 | 0.27 | -0.97 |
|  |  |  | PC2_PC4 | age | 1.68 | 0.27 | -0.97 |
|  |  |  | PC2_PC4 | global | 1.38 | 0.27 | -0.97 |
|  |  |  | PC2_PC5 | age | 1.65 | 0.27 | -0.97 |
|  |  |  | PC2_PC5 | global | 1.68 | 0.27 | -0.97 |
|  |  |  | PC3_PC4 | age | 1.50 | 0.27 | -0.97 |
|  |  |  | PC3_PC4 | global | 2.27 | 0.27 | -0.97 |
|  |  |  | PC3_PC5 | age | 1.00 | 0.27 | -0.97 |
|  |  |  | PC3_PC5 | global | 2.07 | 0.27 | -0.97 |
|  |  |  | PC4_PC5 | age | 1.20 | 0.27 | -0.97 |
|  |  |  | PC4_PC5 | global | 1.14 | 0.27 | -0.97 |
