# Supplementary figures and images for: Dimensionality reduction of quantitative EEG and clinical profiles uncover associations with monogenic neurodevelopmental phenotypes in SNAREopathies
Source: Front Neurosci. 2026 Jan 27;19:1725623. doi: 10.3389/fnins.2025.1725623 (PMC12886349; doi:10.3389/fnins.2025.1725623)

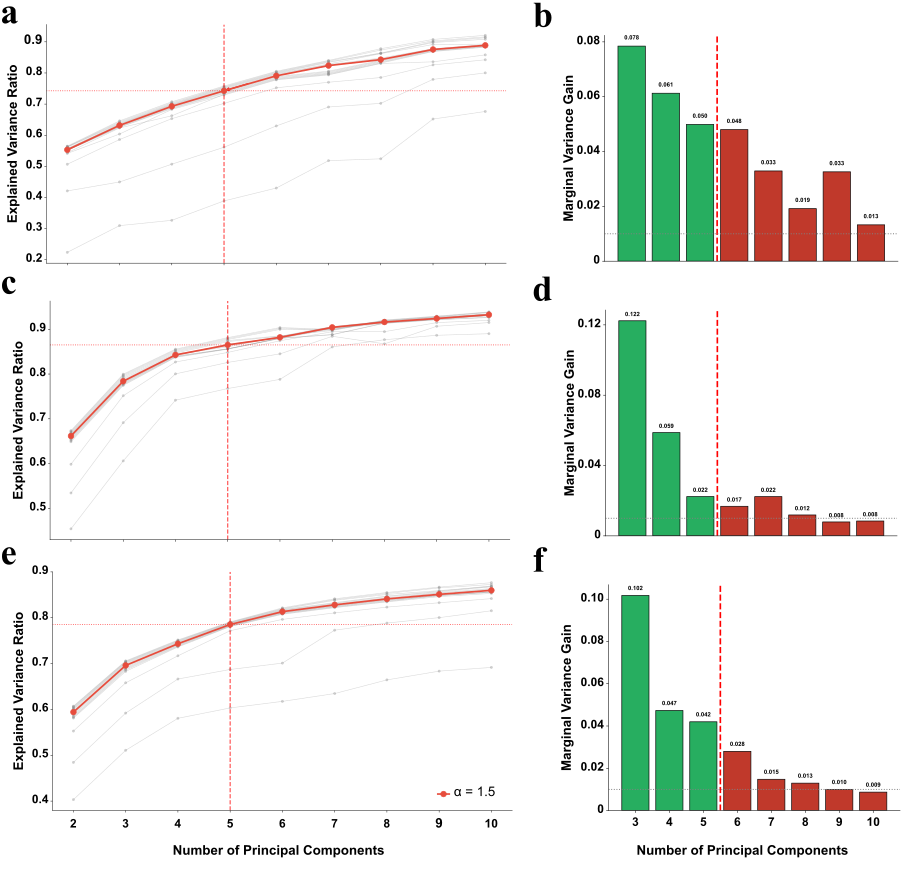

Supplement: Supplementary file 3 [file Image_1.tiff]

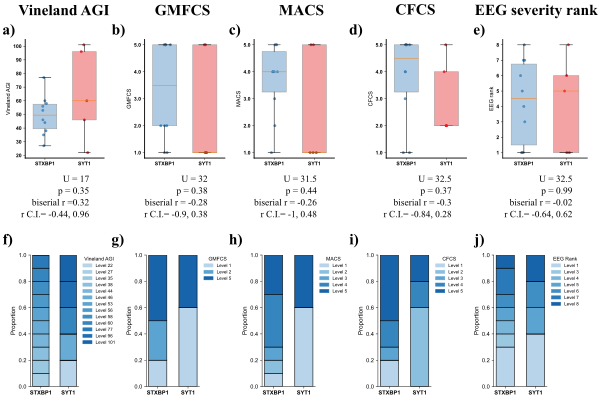

Supplement: Supplementary file 4 [file Image_2.tiff]

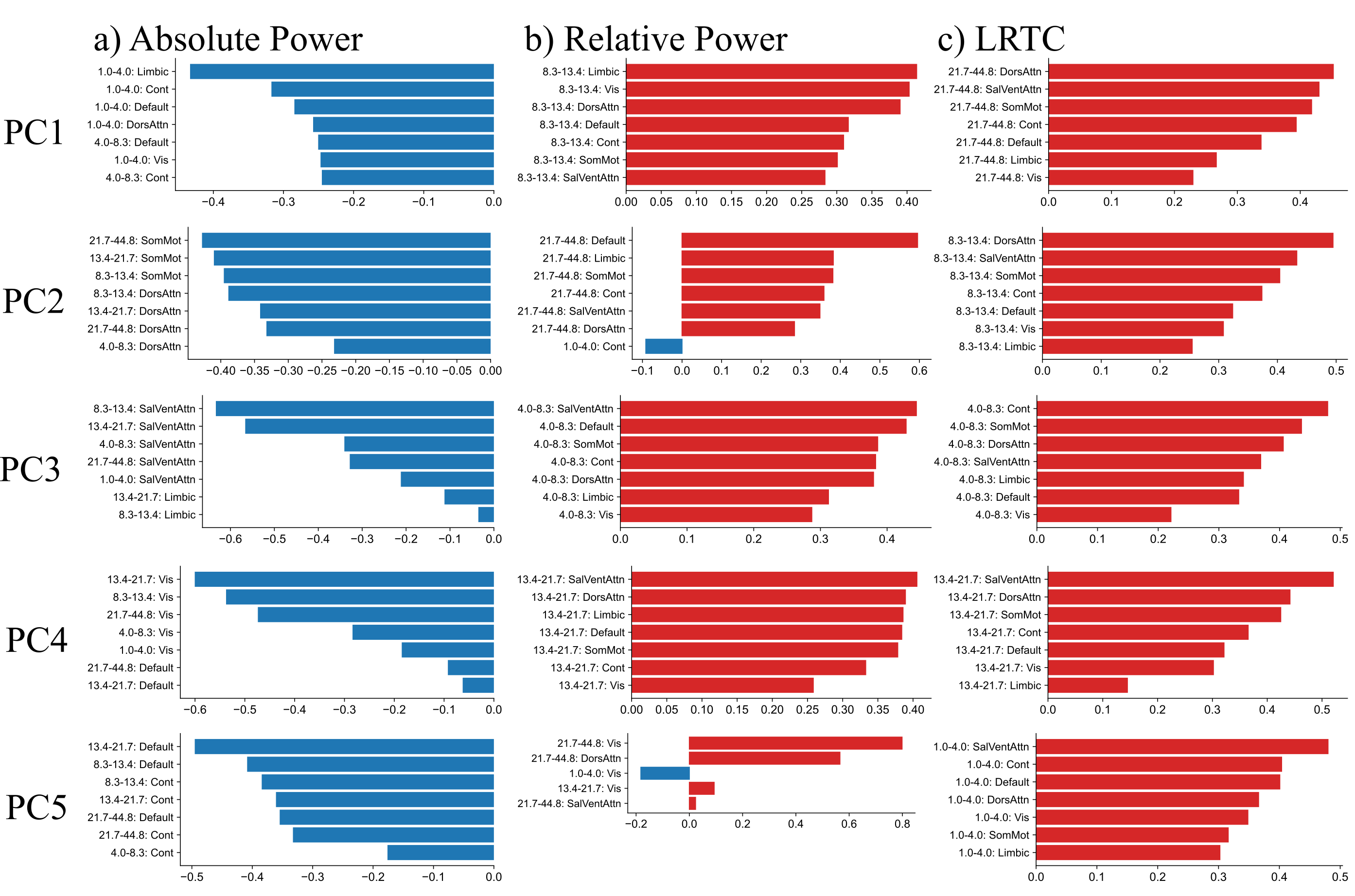

Supplement: Supplementary file 5 [file Image_3.tiff]

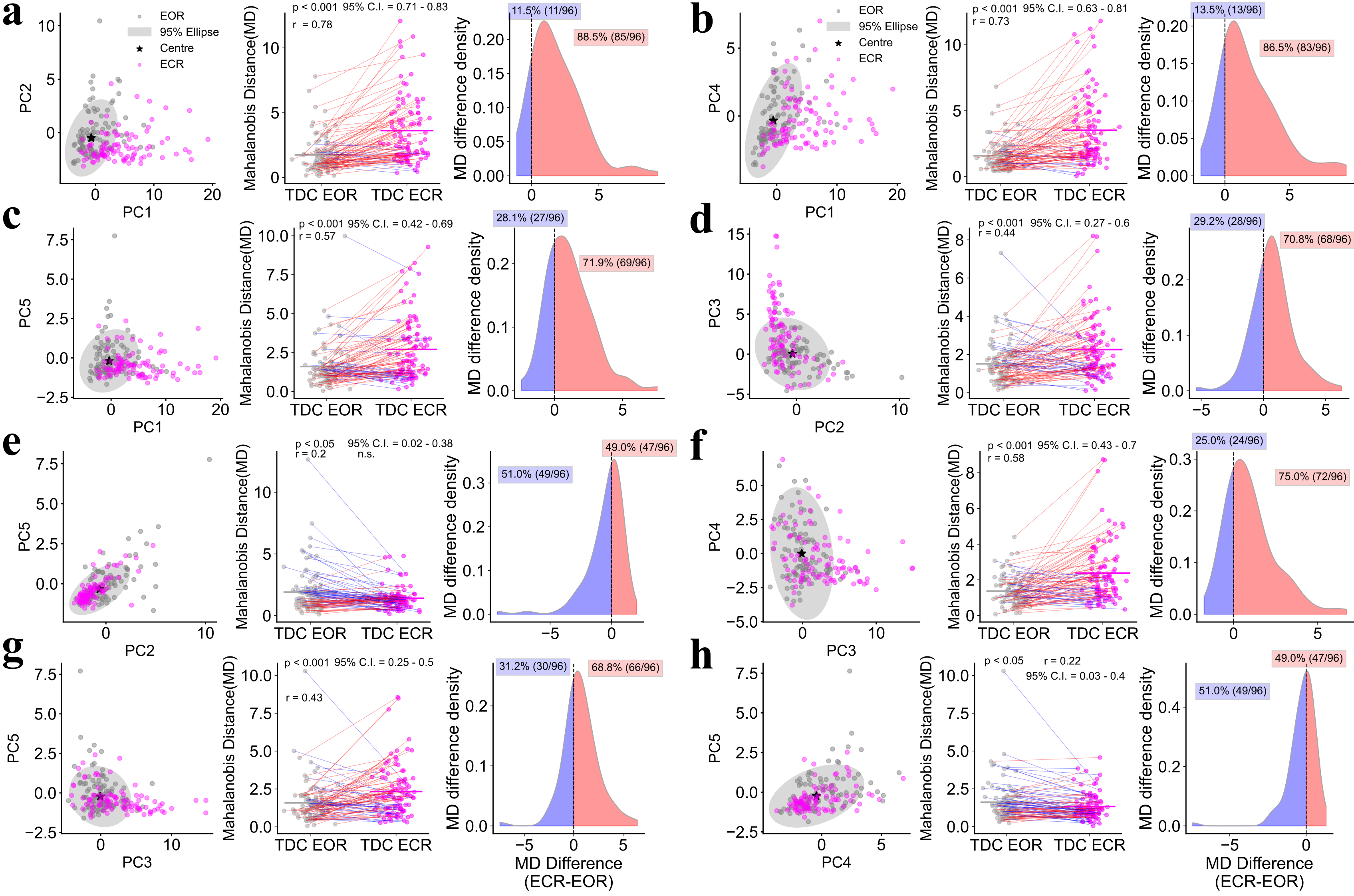

Supplement: Supplementary file 6 [file Image_4.tiff]

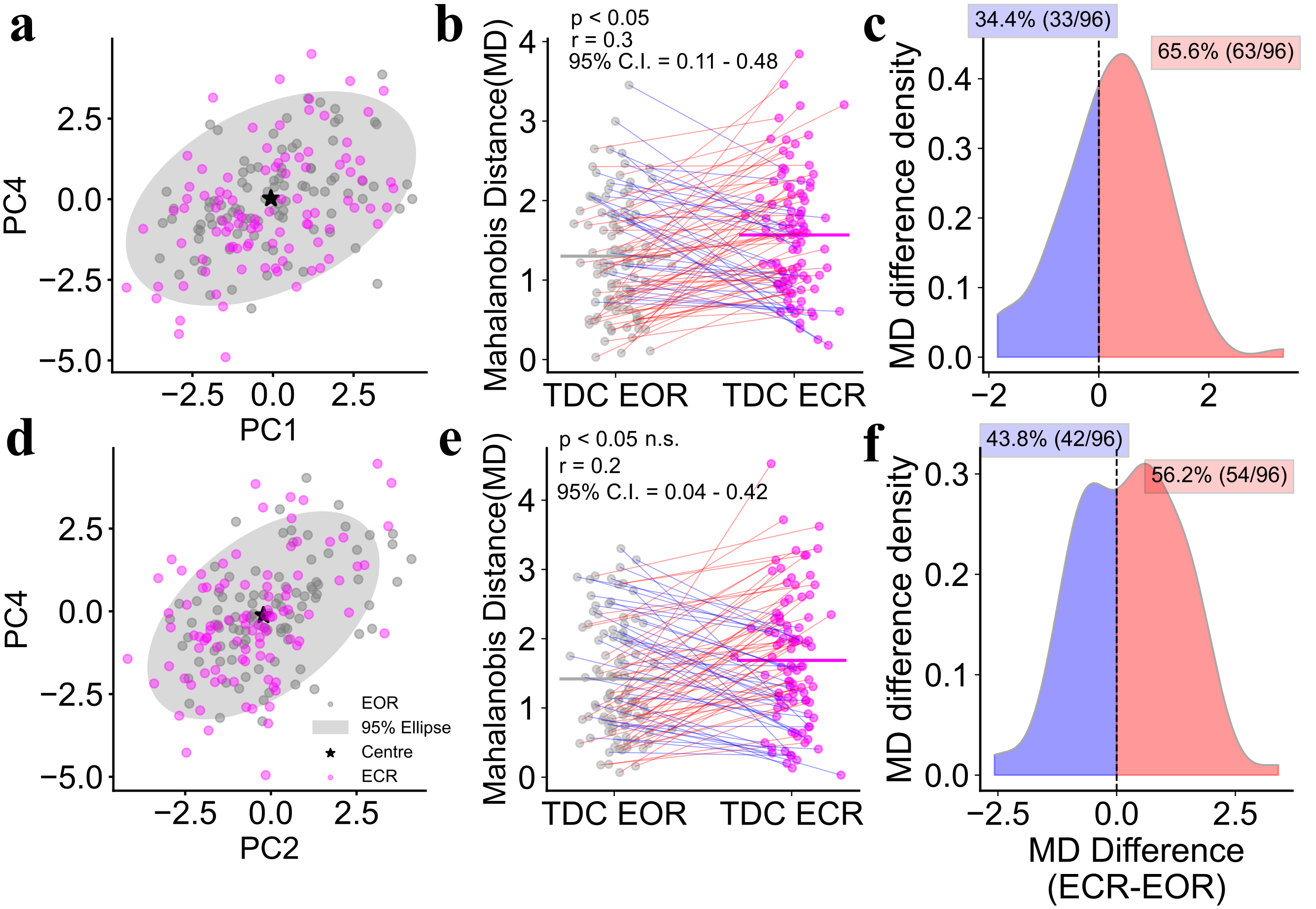

Supplement: Supplementary file 7 [file Image_5.tiff]
